# Transcription-coupled nucleotide excision repair protects against genomic instability and cell death induced by the liver toxin methyleugenol

**DOI:** 10.1038/s41419-026-08853-4

**Published:** 2026-05-19

**Authors:** Caroline Quarz, Riccarda S. Walter, Lydia E. Hens, Max J. Carlsson, Anastasia S. Vollmer, Diana A. Llerena Schiffmacher, Nina Pätzold, Gabriel Ackermann, Daniel Heylmann, Simone Stegmüller, Mohammed Meabed, Alexander T. Cartus, Ivano Amelio, Elke Richling, Wim Vermeulen, Alex Pines, Andriy Khobta, Jörg Fahrer

**Affiliations:** 1 Division of Food Chemistry and Toxicology, Department of Chemistry, Rheinland-Pfälzische Technische Universität (RPTU) Kaiserslautern-Landau, Kaiserslautern, Germany; 2 Rudolf-Buchheim-Institute of Pharmacology, Biomedical Research Center, Justus Liebig University Giessen, Giessen, Germany; 3 Department of Dermatology, University Medical Center, Heidelberg, Germany; 4 Department of Molecular Genetics, Erasmus MC Cancer Institute, Erasmus University Medical Center, Rotterdam, Netherlands; 5 Institute of Nutritional Sciences, Friedrich Schiller University Jena, Jena, Germany; 6 Chair for Systems Toxicology, University of Konstanz, Konstanz, Germany

**Keywords:** Apoptosis, Nucleotide excision repair, Transcription, Gastrointestinal cancer

## Abstract

Methyleugenol (ME) is a hepatotoxic phenylpropene naturally present in various herbs and spices. Following dietary exposure, ME undergoes metabolic activation in the liver, resulting in the formation of DNA adducts and liver damage. Although ME is a suspected human liver carcinogen, it is still unknown which DNA repair pathway removes the ME-induced DNA adducts. Here, we studied the relevance of nucleotide excision repair (NER) using various genetically engineered cell models. Our data show a crucial role for transcription-coupled (TC)-NER rather than global genome (GG)-NER, revealing that ME-induced DNA damage triggers detrimental transcription stress. Mechanistically, ME-derived DNA adducts stall RNA polymerase II (RNAPII), resulting in the chromatin release and cytoplasmic export of its active subunit RPB1, followed by proteasomal degradation to allow for repair and transcription recovery. Blocking of RNAPII by ME-derived DNA lesions promotes CSB immobilization and recruitment of CSA and UVSSA. The triggered canonical TC-NER pathway removes the ME-induced DNA lesions, preserves genome integrity and promotes cell survival. At high DNA adduct levels or in cells with deficient TC-NER, persistent transcription stress provokes genomic instability, induces apoptotic cell death and strongly reduces long-term cell survival. In contrast to that, GG-NER-compromised cells are not sensitized to ME-triggered cytotoxicity. Taken together, the canonical TC-NER pathway is crucial for the repair of DNA adducts induced by ME and likely also structurally related phenylpropenes. These findings are particularly important for Cockayne syndrome patients with defective TC-NER.

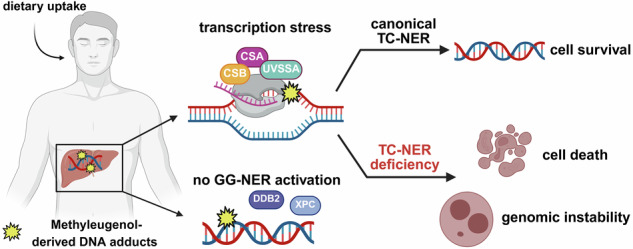

## Introduction

Various DNA repair mechanisms have evolved to protect the genome, which is constantly exposed to DNA-damaging agents both from internal cellular processes and exogenous sources, such as the diet and the environment [1, 2]. The polycyclic aromatic hydrocarbon benzo(a)pyrene (BaP) and the arylamine acetylaminofluorene (AAF) are environmental carcinogens, which cause bulky DNA adducts [3]. These DNA lesions result in a distortion of the DNA helix, which is sensed by the nucleotide excision repair (NER) pathway [4]. NER is subdivided into global genome (GG)-NER, which is active throughout the whole genome, and transcription-coupled (TC)-NER, which is triggered by RNA polymerase II (RNAPII)-stalling DNA lesions on the transcribed strand [4, 5]. Inherited defects in NER genes give rise to human disorders such as Xeroderma Pigmentosum (XP) or Cockayne syndrome (CS). While XP is associated with an extreme sensitivity to sunlight and skin cancer, CS is characterized by premature ageing features, progressive neurodegeneration, as well as frequent liver and kidney dysfunction [5, 6].

Methyleugenol (ME) belongs to the class of phenylpropenes, which also comprises structurally related compounds such as estragole (ES), and is a secondary plant constituent [7]. ME is present in the essential oil of various herbs and spices, such as basil, tarragon, pimento and nutmeg [8, 9]. Food is thus the main source of human exposure to ME, which is absorbed in a rapid and complete manner after oral intake [8, 10]. In the liver, ME is converted to the proximate carcinogen 1’-hydroxymethyleugenol (OH-ME) during phase I metabolism primarily catalyzed by cytochrome P450 1A2 (CYP1A2) [11]. The phase I metabolite OH-ME is subsequently sulfonated to 1’-sulfoxymethyleugenol, which is mediated by sulfotransferase 1A1 (SULT1A1) and SULT1C2 [12]. The phase II metabolite spontaneously decomposes to a highly reactive carbocation, which can then attack DNA bases. This leads to the formation of two main adducts *N*^*2*^-(trans-methylisoeugenol-3’-yl)-2’-deoxyguanosine (*N*^2^-MIE-dG) and *N*^*6*^-(trans-methylisoeugenol-3’-yl)-2’-deoxyadenosine (*N*^6^-MIE-dA) [13]. SULT expression and activity were demonstrated to be crucial for the generation of ME-derived DNA adducts both in vitro and in vivo [12, 14, 15]. Importantly, ME-derived DNA adducts were detected in human liver biopsies as well as in human pulmonary tissue [16, 17]. Long-term rodent studies showed the development of hepatocellular adenoma and carcinoma, hepatoblastoma and neuroendocrine tumours of the glandular stomach after oral administration of ME [18]. Based on these findings, ME was classified by the International Agency for Research on Cancer (IARC) as probably carcinogenic to humans (group 2 A) [19].

We previously showed that ME-triggered DNA adducts cause DNA replication stress and induce apoptosis in liver cells via the p53-bax mitochondrial pathway at high DNA adduct levels [15]. So far, it is unclear whether the ME-derived DNA adducts (*N*^2^-MIE-dG and *N*^6^-MIE-dA) are removed by DNA repair. Due to their chemical structure, these DNA lesions are putative substrates for the NER pathway. This hypothesis is supported by a previous study on ES-induced DNA adducts, which were found at higher levels in hamster fibroblasts with defective NER as compared to wild-type (WT) cells [20].

The aim of our present work was therefore to dissect the presumed role of NER in the removal of *N*^2^-MIE-dG and *N*^6^-MIE-dA adducts and to analyze its impact on cell survival and genomic stability upon OH-ME exposure. Using UHPLC-MS/MS, we demonstrate that a significant fraction of both *N*^2^-MIE-dG and *N*^6^-MIE-dA adducts persists in cells. We further show, using different isogenic human cell lines and primary mouse hepatocytes, that genetic abrogation of the essential NER factor XPA causes hypersensitivity to OH-ME and triggers a robust DNA damage signaling. Cellular toxicity and the observed DNA damage response (DDR) correlate with OH-ME-induced transcription stress, reflected by RNAPII stalling and shutdown of de novo RNA synthesis. Importantly, removal of these DNA lesions depends mainly on TC-NER, whereas GG-NER appears to ignore these DNA lesions. Finally, we show R-loop formation and clastogenicity by ME-triggered DNA adducts, which are increased in TC-NER-deficient cells.

## Results

### OH-ME triggered DNA adduct formation and persistence

**Fig. 1:**
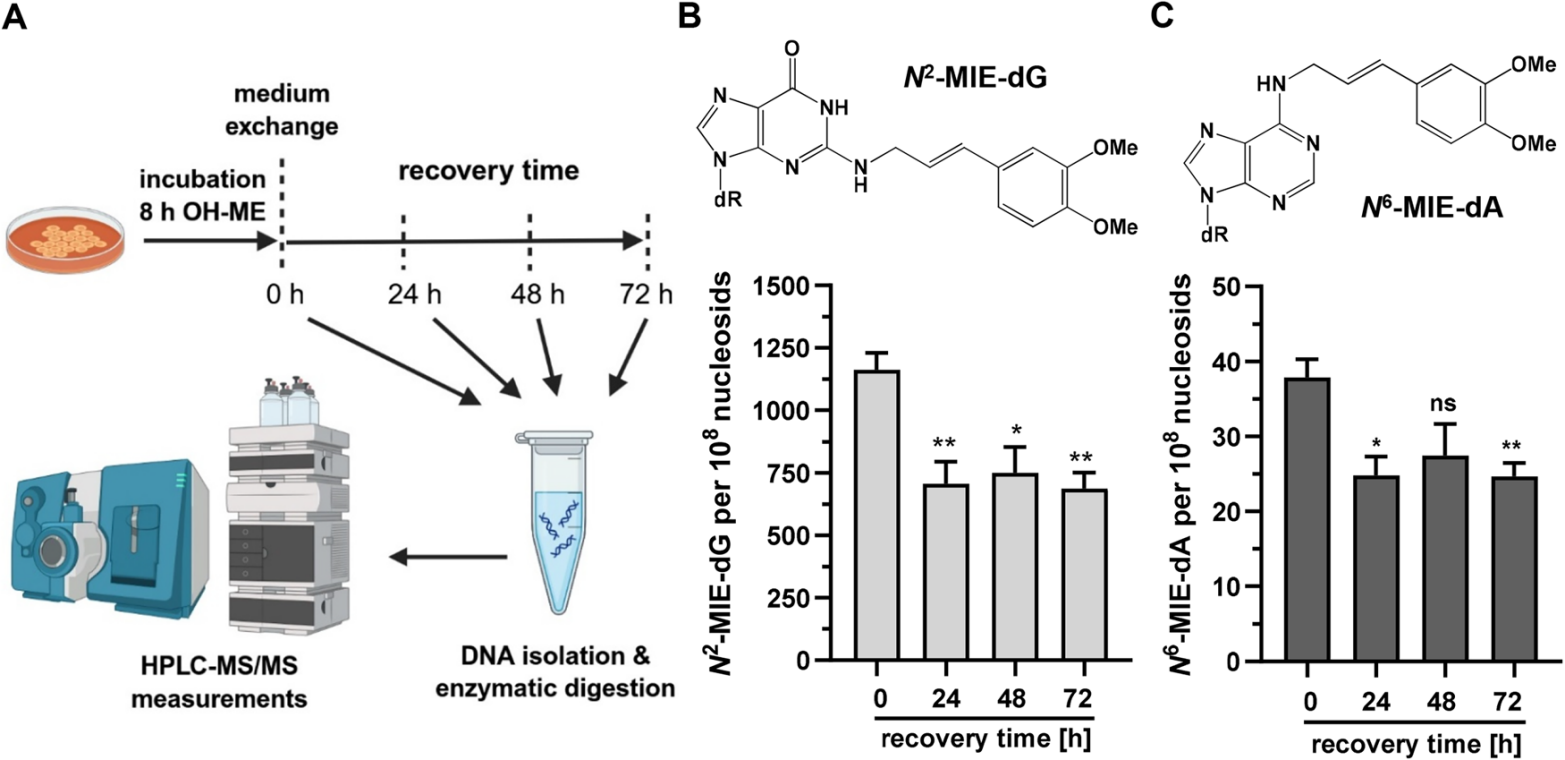
DNA adduct formation and persistence in HepG2 cells after OH-ME treatment. **A** Experimental setup to monitor DNA adduct formation and removal in a recovery assay. Cartoon created in BioRender. Fahrer, J. (2026) https://BioRender.com/nil8ips. **B**, **C** Detection of *N*^2^-MIE-dG and *N*^6^-MIE-dA adducts in human HepG2 cells. The cells were pulse-treated with 25 µM OH-ME for 8 h. After medium exchange, the cells were further cultivated for up to 72 h in fresh medium with reduced FCS content. Genomic DNA was isolated, digested to nucleosides, and adduct levels were measured using stable isotope dilution analysis and UHPLC-MS/MS in the MRM mode (*n* = 4). All data given as mean + SEM (ns *p* > 0.05, *p < 0.05, **p < 0.01).

Initially, HepG2 cells were incubated with OH-ME for up to 24 h to analyze the kinetics of DNA adduct formation. UHPLC-MS/MS measurement showed a time-dependent accumulation of the two main adducts *N*^2^-MIE-dG and *N*^6^-MIE-dA (Fig. S1A, B), which were both readily detectable after 4 h and then increased over time. This finding correlated well with our previous study on OH-ME-triggered DDR, with an early ATR-CHK1 signaling followed by ATM-CHK2 signaling and p53 stabilization [15]. To study not only the formation, but also the possible repair of these DNA adducts, the treatment schedule was modified. To this end, the cells were treated for 8 h with OH-ME, the medium was then replaced by fresh medium with a reduced FCS content, and the cells were cultivated for a recovery period up to 72 h (Fig. 1A). The two main adducts *N*^2^-MIE-dG and *N*^6^-MIE-dA were analyzed (Fig. 1B, C), revealing the highest adduct level directly after the end of treatment without recovery time (0 h). At a concentration of 25 µM OH-ME, 1162 *N*^2^-MIE-dG adducts per 10^8^ nucleosides (ncs) and 38 *N*^6^-MIE-dA adducts/10^8^ ncs were detected (Fig. 1B, C). Both DNA adduct levels then decreased in a comparable manner by 35–40% after a recovery time of 24 h, but persisted until the end of the experiment after 72 h. Here, still 686 *N*^2^-MIE-dG adducts/10^8^ ncs and 27 *N*^6^-MIE-dA adducts/10^8^ ncs were measured. Throughout the entire experiment, the *N*^2^-MIE-dG levels were present in 30-fold excess versus the *N*^6^-MIE-dA adducts. A similar trend was observed for treatment with 2.5 µM OH-ME, but generally lower adduct levels were observed (Fig. S2A, B). Furthermore, the *N*^2^-MIE-dG levels showed a more pronounced decrease by 48% after 24 h of recovery (187 vs. 89 *N*^2^-MIE-dG/10^8^ ncs). The *N*^6^-MIE-dA levels formed upon 2.5 µM OH-ME treatment were very low and declined after 24 h as well (Fig. S2A, B). Notably, neither *N*^2^-MIE-dG nor *N*^6^-MIE-dA was detected in solvent-treated control cells. In summary, the results of the DNA adduct recovery assay revealed only partial repair of the ME-derived adducts, which persisted over 72 h. This striking observation suggests that the majority of OH-ME-induced adducts remain undetected and/or unrepaired by any of the multiple cellular DNA repair systems.

### Impact of XPA on DNA damage response and cytotoxicity induced by OH-ME

**Fig. 2:**
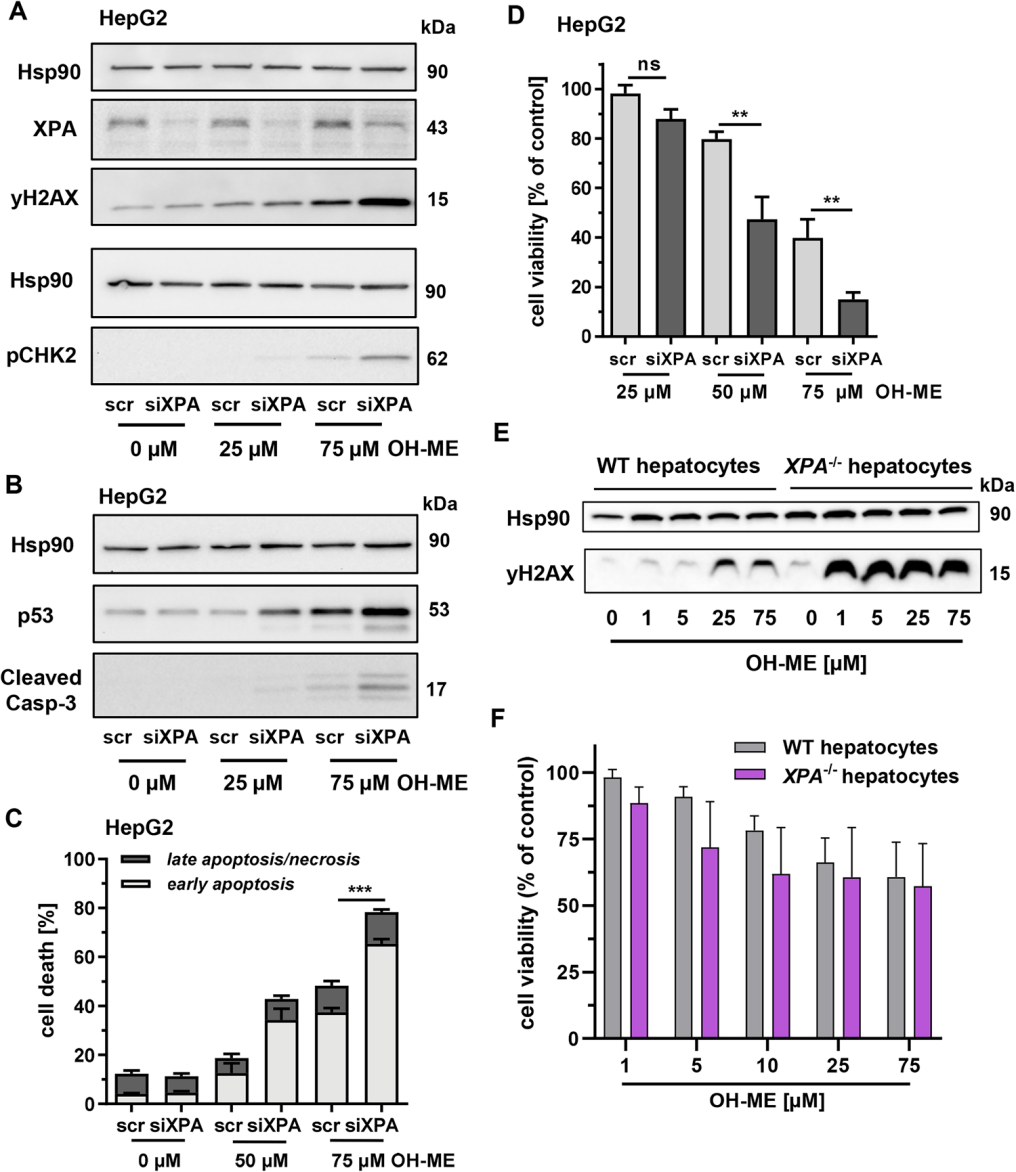
Role of XPA-mediated NER in DNA damage response and cytotoxicity triggered by OH-ME. Transient knockdown of *XPA* in HepG2. Cells were transfected with *XPA* siRNA or scrambled (scr) RNA followed by OH-ME treatment. Representative western blot experiments in HepG2 cells with *XPA* knockdown and subsequent OH-ME treatment (0-75 µM) for 24 h. The samples were analyzed by SDS-PAGE and western blot detection of XPA, γH2AX, pCHK2 (**A**), p53 and cleaved caspase-3 (**B**). Hsp90 served as loading control. **C** Cell death induction after *XPA* knockdown and OH-ME treatment (0-75 µM) for 72 h in HepG2 cells. Apoptotic and necrotic cell death was determined by Annexin V-FITC/PI staining and flow cytometry (*n* = 3, except for 50 µM, *n* = 2). **D** Impact of siRNA-mediated *XPA* knockdown and OH-ME treatment on viability in HepG2 cells. Cells were exposed to increasing concentrations of OH-ME (0-75 µM) for 72 h. Viability was then measured by MTS assay (*n* = 4). **E** OH-ME triggered DNA damage response in primary murine hepatocytes (PMH) proficient or deficient for *XPA*. PMH were exposed to increasing concentrations of OH-ME (0–75 µM) for 24 h and subjected to western blot analysis of γH2AX. Hsp90 served as a loading control. A representative western blot is shown (*n* = 3). **F** Viability of WT and *XPA*^–/–^ PMHs after OH-ME treatment (0–75 µM) for 24 h. Cell viability was determined by the resazurin reduction assay (*n* = 6 for WT, *n* = 3 for *XPA*^–/–^). All data given as mean + SEM (ns *p* > 0.05, **p < 0.01, ***p < 0.001).

Due to the chemical structure of the formed ME-derived DNA adducts and previous evidence from the related phenylpropene ES [20], we hypothesized that the NER pathway is engaged by ME-derived DNA adducts. To test this, the essential NER gene *XPA* was transiently downregulated by siRNA in HepG2 cells. The OH-ME-triggered DDR was then analyzed upon *XPA* knockdown, which revealed a substantial reduction of XPA levels (Figs. 2A and S3A). After incubation with OH-ME for 24 h, the DDR markers γH2AX and pCHK2 strongly increased upon *XPA* knockdown in HepG2 cells treated with 75 µM OH-ME (Figs. 2A and S3B, C). Similar effects were observed for the genotoxic stress marker p53 and the apoptosis marker cleaved caspase-3, which were both elevated in *XPA* knockdown HepG2 cells upon OH-ME treatment (Figs. 2B and S3D, E). In order to corroborate the observed increase in the apoptosis marker, cell death induction was investigated using Annexin V-FITC/PI staining upon *XPA* knockdown. In line with the western blot results, treatment with 75 µM OH-ME for 72 h caused a strong increase in apoptotic cells after *XPA* knockdown (65% vs. 37% in scrRNA control cells) (Figs. 2C and S3F). The higher sensitivity of the *XPA* knockdown cells towards OH-ME was confirmed by cell viability measurements, which were also performed after 72 h of incubation (Fig. 2D). Western blot analysis showed the efficient and long-lasting siRNA-mediated *XPA* downregulation as compared to control cells transfected with scrRNA (Fig. S3G). To validate these findings, we used primary murine hepatocytes (PMH) obtained from WT and *XPA* knockout (k.o.) mice that were exposed to OH-ME for 24 h. In WT-PMH, OH-ME caused a concentration-dependent formation of the DDR marker γH2AX. In contrast to that, massively increased γH2AX levels were observed already at the lowest OH-ME concentration in *XPA*^*–/–*^ PMH (Figs. 2E and S4A). Western blot analysis of different tissue samples revealed comparable SULT levels between WT and *XPA*^*–/–*^ hepatocytes (Fig. S4B), which is important in view of the crucial role of SULTs for the metabolic activation of OH-ME and DNA adduct formation. Finally, we performed viability measurements in WT vs. *XPA*^*–/–*^ hepatocytes exposed to OH-ME, revealing a moderately elevated cytotoxicity in *XPA*^–/–^ cells (Fig. 2F), which was also visible using light microscopy (Fig. S4C). In summary, our results in genetically modified liver cell models with *XPA* knockdown or *XPA* k.o. clearly establish the involvement of NER in the removal of ME-derived DNA adducts.

### Relevance of GG-NER and TC-NER for removal of ME-derived DNA adducts

To analyze the contribution of the NER subpathways, we used *DDB2*^*–/–*^ (compromised GG-NER) and *ERCC8*/*CSA*^*–/–*^ (deficient TC-NER) HeLa cell models. Initially, we re-authenticated the k.o. cell models by western blot analysis, confirming the lack of CSA and DDB2, respectively (Fig. S5A). Furthermore, the genetically engineered isogenic cell lines were characterized with regard to their UV sensitivity, which was strongly increased in *CSA*^–/–^ cells (Fig. S5B). *DDB2*^–/–^ cells displayed a similar UV sensitivity as WT cells (Fig. S5B), which is in line with the reported resistance of *DDB2*^–/–^ cells to UV-induced apoptosis [21]. Treatment with OH-ME for 48 h induced a concentration-dependent decrease in viability in all three cell lines. However, *CSA*^*–/–*^ cells were by far the most sensitive, whereas *DDB2*^–/–^ cells were similar to WT (Fig. S5C). In agreement with these findings, western blot analysis revealed elevated levels of γH2AX and cleaved caspase-3 upon OH-ME treatment (100 and 500 µM) only in *CSA* k.o. cells (Fig. S5D). Notably, higher OH-ME concentrations were used in HeLa cells due to their lower SULT expression as compared to HepG2 cells.

**Fig. 3:**
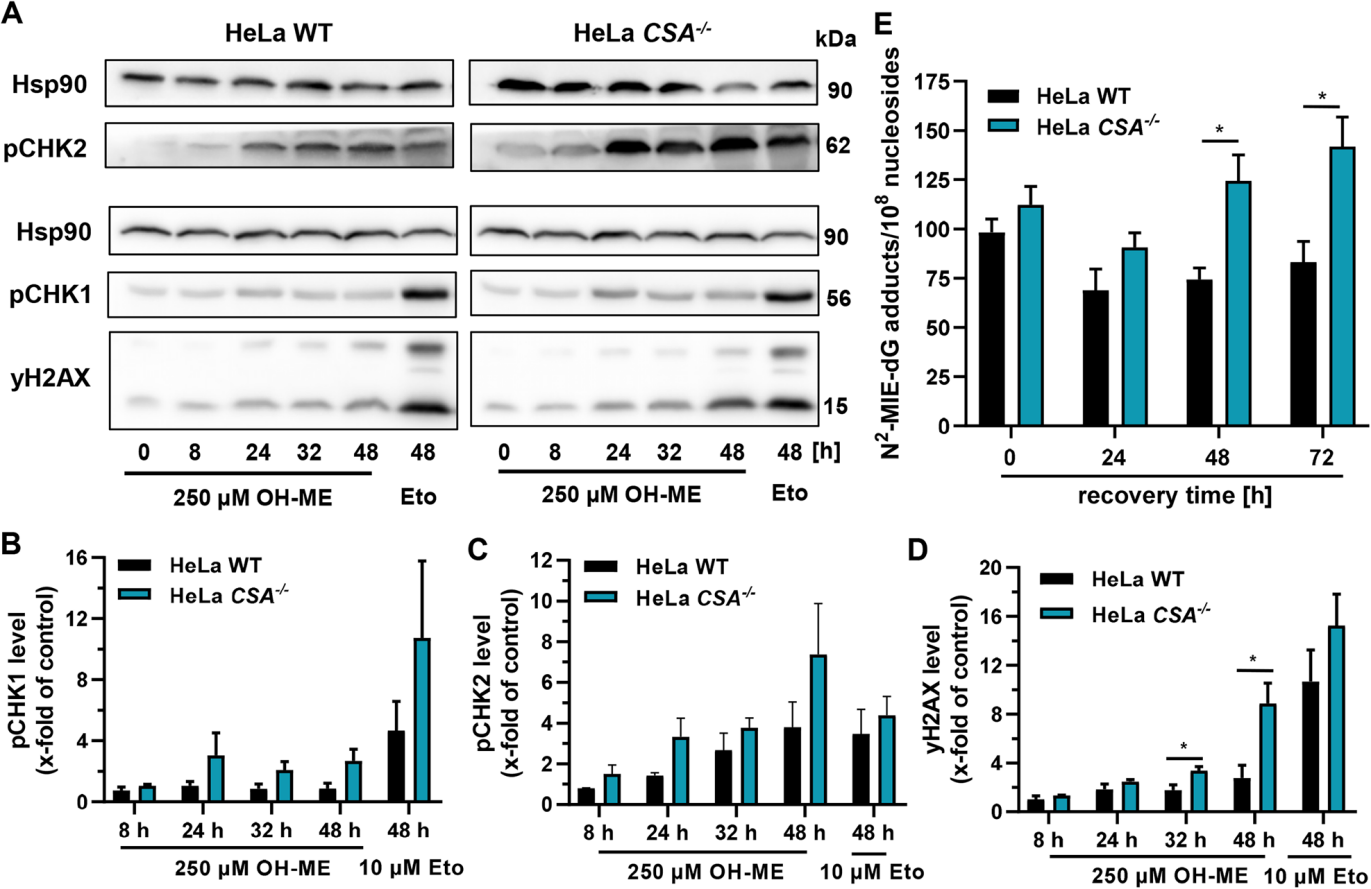
Influence of CSA-dependent TC-NER on DNA adduct formation and DNA damage response triggered by OH-ME. Time-dependent DNA damage response in HeLa WT and *CSA*^*–/–*^ cells upon exposure to 250 µM OH-ME for up to 48 h. Etoposide (Eto; 10 µM) was included as positive control. Representative western blots (**A**) and densitometric evaluation of pCHK1 (**B**), pCHK2 (**C**) and γH2AX (**D**) levels. Hsp90 served as loading control (*n* = 4). **E** Formation and persistence of *N*^2^-MIE-dG adducts in HeLa WT and HeLa *CSA*^–/–^. The cells were treated with 500 µM OH-ME for 8 h. After medium exchange, cells were further cultivated for up to 72 h in fresh medium with reduced FCS content. Genomic DNA was isolated, digested to nucleosides, and adduct levels were measured using stable isotope dilution analysis and UHPLC ESI-MS/MS in the MRM mode (*n* = 4). All data given as mean + SEM (*p < 0.05).

As a next step, the DDR triggered by OH-ME was analyzed in a time-dependent manner in HeLa cells proficient or deficient for *CSA*. A moderately elevated phosphorylation of CHK1 was observed in HeLa *CSA*^–/–^ cells as compared to WT cells after incubation for ≥24 h (Fig. 3A, B). Furthermore, a time-dependent increase in pCHK2 and γH2AX levels was detected in HeLa *CSA*^–/–^ cells, which culminated after 48 h (Fig. 3A, C, D). These effects were also found in WT cells, albeit at significantly lower levels. The anticancer drug and positive control etoposide exerted stronger DDR effects in the *CSA*-deficient cell line as compared to WT cells (Fig. 3B–D). We then detailed the role of TC-NER using a DNA adduct recovery assay. To this end, HeLa cells were treated with 500 µM OH-ME for 8 h, followed by medium exchange and incubation in fresh medium with a reduced FCS content for up to 72 h. In HeLa WT cells (Fig. 3E), a similar kinetic was observed as in HepG2 cells (Fig. 1B). Directly after treatment, the *N*^2^-MIE-dG adduct level amounted to 98 adducts/10^8^ ncs (Fig. 3E), which then declined to 69 adducts/10^8^ ncs after 24 h, followed by a slight increase to 83 adducts/10^8^ ncs after 72 h (Fig. 3E). In HeLa *CSA*^*–/–*^, a higher *N*^2^-MIE-dG adduct level with 112 adducts/10^8^ ncs was detected directly after the OH-ME treatment. After an initial decline to 91 adducts/10^8^ ncs after 24 h, the adduct levels rose again from 125 adducts/10^8^ ncs (48 h) up to 142 adducts /10^8^ ncs at the end of the recovery time (Fig. 3E). The further moderate rise of DNA adduct levels after OH-ME withdrawal might be attributable to a slower SULT-mediated phase II metabolism in HeLa cells than in HepG2 cells. For 100 µM OH-ME, the induced *N*^2^-MIE-dG adduct levels were in the range of 18–37 adducts/10^8^ ncs, whereby the levels were always higher in HeLa *CSA*^*–/–*^ as compared to HeLa WT (Fig. S6A). It should be mentioned that only the main adduct *N*^2^-MIE-dG could be analyzed in HeLa cells due to the generally lower adduct levels as compared to HepG2 cells. Collectively, our data showed an accumulation of ME-derived DNA adducts in *CSA*^–/–^ cells deficient for TC-NER, resulting in an increased DDR activation. In contrast to that, *DDB2*^–/–^ cells compromised in GG-NER were not sensitized towards OH-ME, suggesting that this NER subpathway is not activated by ME-derived DNA adducts.

### Influence of TC-NER deficiency on OH-ME-triggered cytotoxicity

**Fig. 4:**
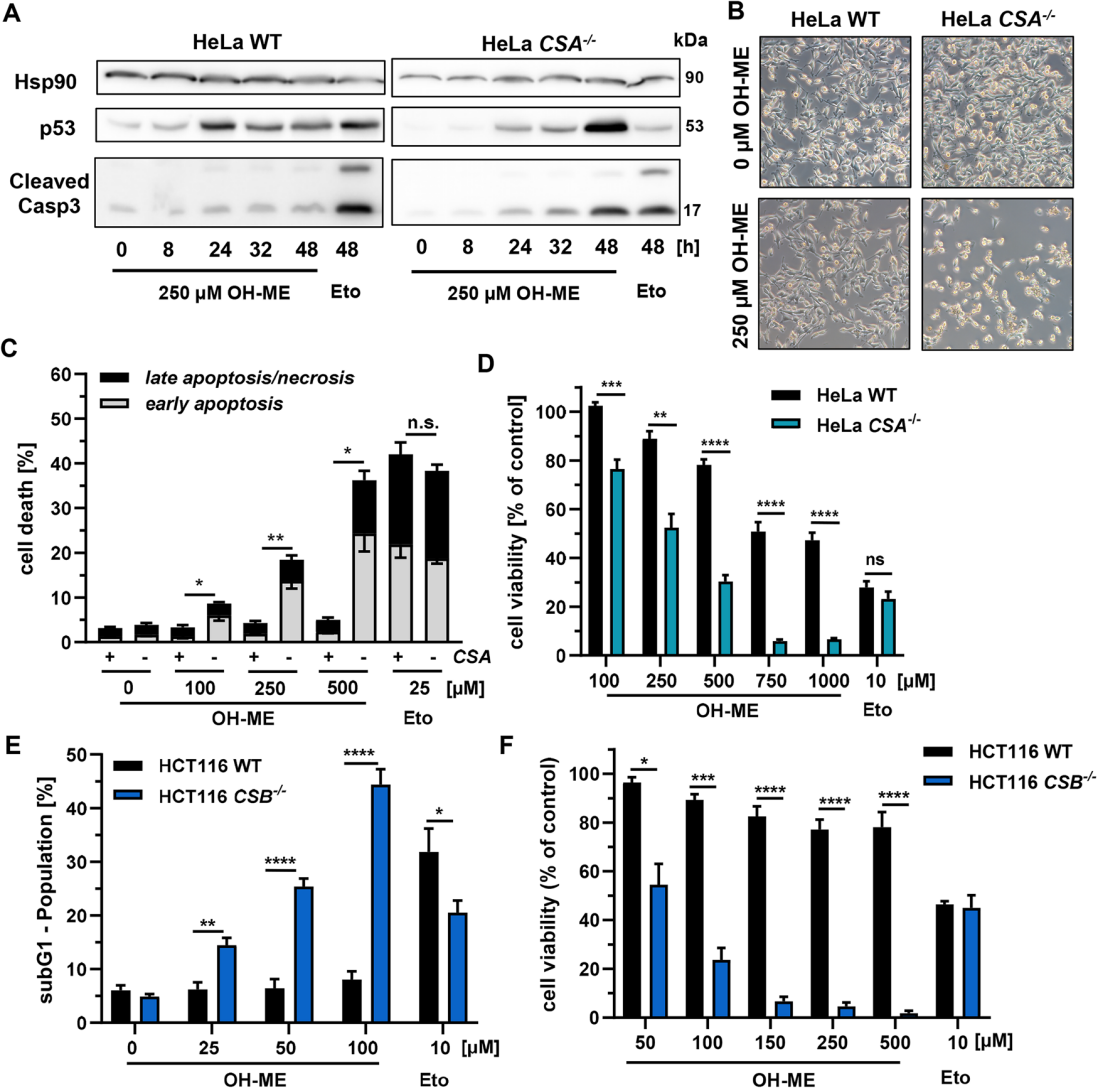
Cell death induction by OH-ME depending on the TC-NER status. **A** Time-dependent western blot analysis of cell death markers (cleaved caspase-3, p53) in HeLa WT and *CSA*^*–/–*^ incubated with 250 µM OH-ME for up to 48 h. Etoposide (Eto; 10 µM) was included as positive control. Representative western blots are shown. Hsp90 was detected as a loading control (*n* = 4). **B** Representative light microscopy images of HeLa WT and HeLa *CSA*^–/–^ cells treated with 250 µM OH-ME or solvent control (0 µM) for 48 h (10x magnification). **C** Cell death induction in HeLa WT and HeLa *CSA*^*–/–*^ upon OH-ME treatment. Cells were exposed to OH-ME (0–500 µM) for 48 h followed by Annexin V-FITC/PI staining and flow cytometry (*n* = 4). **D** Viability of HeLa WT and HeLa *CSA*^*–/–*^ cells after treatment with increasing concentrations of OH-ME (0-1000 µM) for 48 h. Cell viability was assessed using resazurin reduction assay (*n* = 4). **E** Analysis of subG1 population in HCT116 WT and HCT116 *CSB*^*–/–*^ exposed to OH-ME (0-100 µM) for 48 h. SubG1 population indicative of cell death was assessed by PI staining and flow cytometry (*n* = 4). **F** Viability of HCT116 WT and HCT116 *CSB*^*–/–*^ cells upon treatment with increasing concentrations of OH-ME (0–1000 µM) for 48 h. Cell viability was determined by the resazurin reduction assay (*n* = 4). All data are given as mean + SEM. ns *p* > 0.05 *p < 0.05, **p < 0.01, ***p < 0.001, ****p < 0.0001.

We then studied the cytotoxic potential of OH-ME with regard to the TC-NER status. To this end, HeLa WT and HeLa *CSA*^*–/–*^ cells were treated with OH-ME for up to 48 h and subjected to western blot analysis of p53 and cleaved caspase-3. Interestingly, p53 stabilization occurred in a time-dependent manner in both cell models (Figs. 4A and S6B). However, the p53 levels after OH-ME exposure increased much stronger in *CSA* k.o. cells from the basal level as compared to the WT after 48 h (Figs. 4A and S6B). Cleaved caspase-3 also accumulated in a time-dependent manner but showed the strongest signal in *CSA* k.o. cells after 48 h, similar to the effects on p53 (Figs. 4A and S6C). These findings indicate cell death induction at the later time points, which was supported by microscopy images, revealing a strong reduction in cell density and morphological changes in HeLa *CSA*^*–/–*^ treated with OH-ME (Fig. 4B). Little morphological differences were observed between WT and *CSA* k.o. cells challenged with etoposide (Fig. S6D). Subsequently, cell death analysis was performed by AnnexinV-FITC/PI staining and flow cytometry. These experiments showed a concentration-dependent increase in both apoptotic and necrotic cells in HeLa *CSA*^*–/–*^ exposed to OH-ME, whereas almost no cytotoxic effect was detected in HeLa WT cells (Figs. 4C and S6E). It should be noted that the positive control etoposide caused similar cell death induction independent of the *CSA* status (Figs. 4C and S6E), which is in line with the notion that etoposide acts in a rather transcription-independent manner at low concentrations [22]. In accordance with the AnnexinV-FITC/PI measurements, significant differences between the cell lines treated with OH-ME were also seen in viability experiments. While at the highest OH-ME concentration (1000 µM) the viability in WT cells dropped only by around 50%, a much stronger concentration-dependent decrease was detected in HeLa *CSA*^*–/–*^, with only 7% viability at 1000 µM (Fig. 4D). With increasing incubation time, the differences between the TC-NER proficient and deficient cell lines became even more evident (Fig. S7A, B), which was substantiated by the calculated EC_50_ values (Table S3).

To confirm the results in another cell model, the colorectal cancer cell line HCT116 was included, which was genetically engineered for either *CSA* or *CSB* k.o. [23] and re-authenticated by western blot detection (Fig. S8A). CSB acts upstream of CSA and is the first factor recruited to stalled RNAPII in order to initiate TC-NER [24]. Cell death induction was investigated using subG1 analysis, which revealed almost no increase in the subG1 population in HCT116 WT cells (Figs. 4E and S8D). In contrast to that, TC-NER deficient *CSB*^–/–^ HCT116 cells showed a concentration-dependent increase in the subG1 population, with up to 45% at 100 µM OH-ME (Figs. 4E and S8D). The positive control etoposide induced higher subG1 population in WT as compared to *CSB*^–/–^ cells (32% vs. 21%) (Fig. 4E). The strongly increased sensitivity of *CSB*-deficient cells towards OH-ME was also reflected in the cell viability assays, in which 150 µM OH-ME caused little effects in WT cells, but strongly reduced viability below 10% in HCT116 *CSB*^–/–^ cells (Fig. 4F). Similar effects were observed in HCT116 *CSA*^*–/–*^ cells, as attested by subG1 measurements and viability assay (Fig. S8B, C). These findings were substantiated by calculation of the EC_50_ values in the different cell models (Table S3). In summary, our results demonstrate that TC-NER mediated by CSA and CSB is crucial for the protection of cells against the cytotoxic effects of ME-derived DNA damage.

### Transcription stress triggered by OH-ME and the role of CSA

**Fig. 5:**
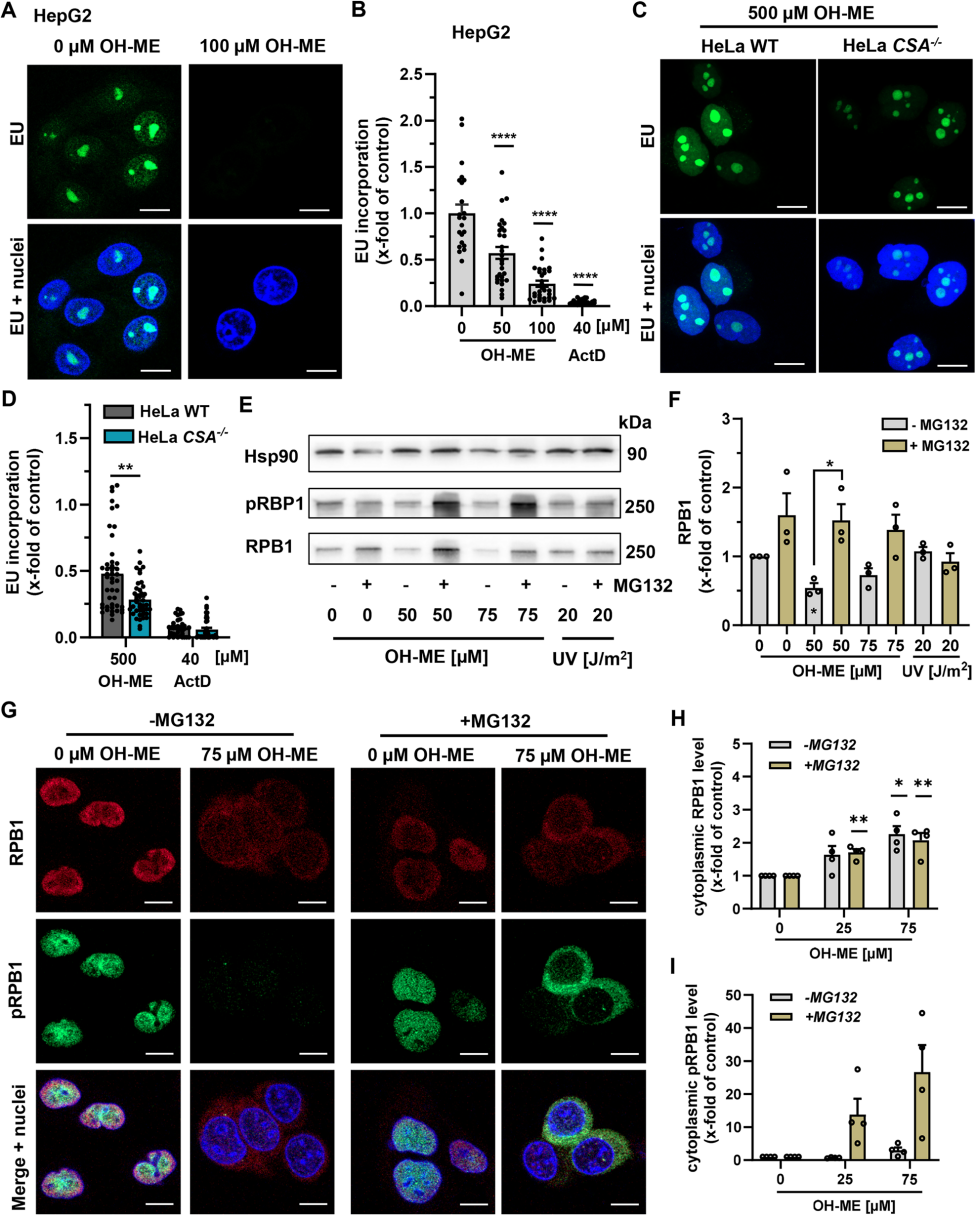
Transcription stress triggered by OH-ME and role of CSA. Assessment of de novo RNA synthesis upon OH-ME treatment for 24 h in HepG2 (**A, B**) and HeLa WT versus HeLa *CSA*^*–/–*^ cells (**C, D**). Actinomycin D (ActD) was included as positive control. EU incorporation was analyzed by click chemistry with FAM-Azide (green), while nuclei were visualized by DAPI. Images were acquired by confocal microscopy and processed by Zen software. Representative images (**A, C**, scale bar: 10 µm) and the quantitative evaluation (**B, D**) are shown (*n* = 3–5). **E**, **F** Western blot detection of RPB1 and phospho-Ser2/Ser5 RPB1 (pRPB1) in HepG2 cells upon OH-ME treatment for 24 h in the absence or presence of the proteasome inhibitor MG132 (10 µM, added 6 h before cell harvest). In addition, HepG2 cells were exposed to UV-C (20 J/m^2^) followed by 2 h incubation with or without MG132. Representative western blots (**E**) and densitometric evaluation of RPB1 levels (**F**) are shown. Hsp90 served as loading control (*n* = 3). **G**–**I** Subcellular localization of RPB1 and pRPB1 in HepG2 cells 24 h after OH-ME treatment with or without proteasome inhibition by MG132 (10 µM, added 6 h prior to cell harvest). Cells were stained for RPB1 (red) and pRPB1 (green), while the nuclei were visualized by DAPI (blue). Images were acquired by confocal microscopy and processed by Zen software. Representative images (**G**, scale bar: 10 µm) and the quantitative evaluation of cytoplasmic RPB1 (**H**) and pRPB1 (**I**) levels are shown (*n* = 4). All data are given as mean + SEM. *p < 0.05, **p < 0.01, ****p < 0.0001.

Our previous results indicated that ME-derived DNA adducts block transcription and thereby trigger CSA/CSB-dependent TC-NER. To investigate this in more detail, a 5-ethynyl-uridine (EU) incorporation assay was used to monitor de novo RNA synthesis. Treatment of HepG2 cells with OH-ME decreased the EU incorporation in a concentration-dependent manner, with a 75% reduction to the level in control cells after 100 µM OH-ME (Fig. 5A, B). Treatment with the positive control actinomycin D, which intercalates in DNA and thereby blocks RNA polymerases, led to an almost complete abrogation of RNA synthesis in HepG2 cells (Figs. 5B and S9A). In both HeLa WT and *CSA*^–/–^ cells, OH-ME caused a concentration-dependent suppression of EU incorporation (Figs. 5C, D and S9B). While no differences between the HeLa WT and *CSA*^–/–^ cells were seen after treatment with 250 µM OH-ME (Fig. S9C), the higher OH-ME concentration (500 µM) revealed a stronger inhibition of de novo RNA synthesis in HeLa cells with *CSA* deficiency (Fig. 5C, D). Next, we measured the levels of RPB1, the active subunit of RNAPII, as well as its phosphorylation on Ser-2/Ser-5 (pRPB1) in whole cell lysates. Our experiments showed that both RPB1 and pRPB1 decreased upon OH-ME treatment, whereas no effect on (p)RPB1 levels was observed upon UV irradiation (Fig. 5E, F). Interestingly, the proteasomal inhibitor MG132 blocked the OH-ME-triggered degradation of RPB1 and pRPB1 (Figs. 5E, F and S10A). These findings were corroborated by confocal microscopy, which revealed nuclear depletion and cytoplasmic export of (p)RPB1 following OH-ME treatment (Figs. 5G–I and S10B). In the presence of MG132, nuclear levels of (p)RPB1 increased again (Fig. S10C, D). Furthermore, the cytoplasmic levels of pRPB1 were strongly augmented by MG132, indicating preferential proteasomal degradation of pRPB1 in response to OH-ME (Fig. 5G–I). Taken together, the OH-ME-derived DNA damage caused stalling of RNAPII, resulting in proteasomal degradation of its subunit RPB1 and a shutdown of de novo transcription in HepG2 cells.

### ME-derived DNA adducts trigger the canonical TC-NER pathway to restore transcription

**Fig. 6:**
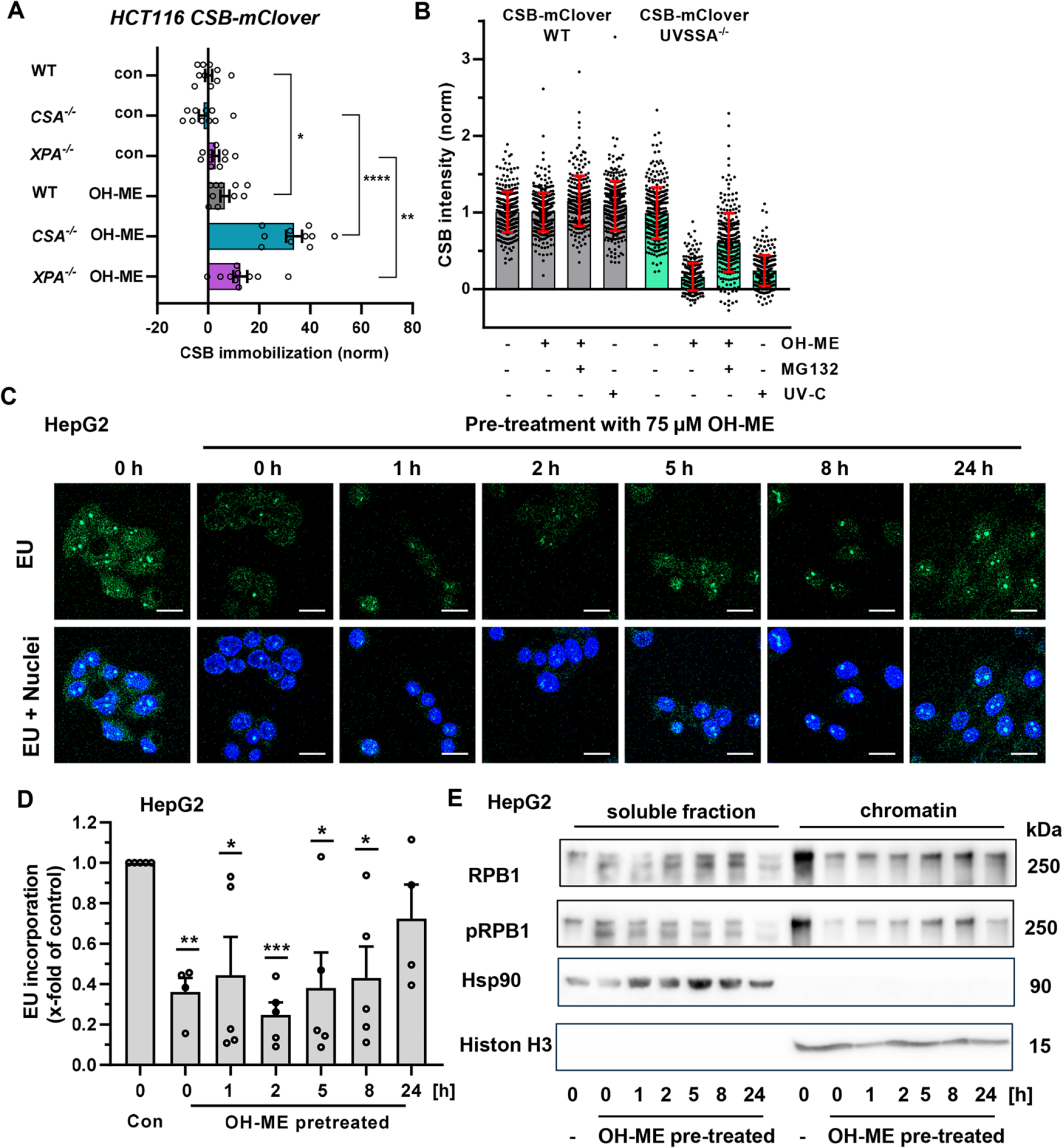
Regulation of CSB in response to OH-ME triggered transcription-blocking lesions and recovery of transcription. **A** FRAP analysis of CSB-mClover in HCT116-WT, *CSA*^*–/–*^ and *XPA*^*–/–*^ cells exposed to 25 µM OH-ME for 16 h. The percentage of CSB-mClover immobile fraction was determined by FRAP analysis (*n* = 3). **B** Endogenous CSB-mClover fluorescence in HCT116 CSB-mClover WT and *UVSSA*^–/–^. Cells were mock-treated or exposed to 25 μM OH-ME with or without 2 μM MG132 for 16 h, or UV-C irradiated (4 J/m^2^) followed by 16 h recovery prior to fixation and DAPI staining. Fluorescence was background-corrected using non-fluorescent HCT116 WT cells and normalized to non-irradiated condition (*n* = 3). **C**, **D** Evaluation of the de novo RNA synthesis after OH-ME treatment and recovery time in HepG2. Cells were incubated with 75 µM OH-ME for 16 h. After a recovery period of 0-24 h the cells were fixed, processed and the EU incorporation (green) analyzed as described. Images were captured by confocal microscopy and processed by Zen software. Representative images (**C**, scale bar: 10 µm) and the quantitative evaluation of EU incorporation (**D**) are shown (*n* = 5). **E** Analysis of soluble and chromatin-bound (p)RPB1 in HepG2 cells. Cells were treated with 75 µM OH-ME for 16 h and then harvested after 0-24 h of recovery time, followed by cell fractionation. The cytosolic marker Hsp90 and the chromatin marker Histone H3 served as loading controls. Representative western blot images are shown (*n* = 5). All data are given as mean + SEM, except for panel B (mean ± SD). *p < 0.05, **p < 0.01, ***p < 0.001, ****p < 0.0001.

To further analyze the transcription-blocking potential of ME-derived DNA adducts, we monitored the immobilization of endogenously tagged CSB on chromatin using live cell imaging by performing FRAP experiments. We have previously shown that upon UV-C irradiation, known to block RNAPII, fluorescently tagged (here GFP derivative mClover) CSB gets immobilized on UV-damaged chromatin, reflecting its engagement in TC-NER [23]. We observed a moderate immobilization of CSB following treatment of HCT116 CSB-mClover WT cells with 25 µM OH-ME (Figs. 6A and S11A). Strikingly, the levels of immobilized CSB in response to OH-ME were strongly potentiated in the *CSA*^*–/–*^ background (Fig. 6A). This is most likely explained by the fact that—in the absence of CSA—the binding of CSB to stalled RNAPII is not changed, but its dissociation is impaired, illustrating the activation of the canonical CSA- and CSB-dependent TC-NER pathway. Increased immobilization of CSB was also observed in *XPA*^*–/–*^ cells (Fig. 6A), but not in cells with downregulated XPC (Fig. S11B, C), further suggesting that GG-NER is not involved in the removal of ME-derived DNA adducts. These findings were supported by western blot analysis of the DDR markers p53 and γH2AX, which moderately increased in HCT116 CSB-mClover WT cells in a time-dependent manner (Fig. S12A–C, left panel). Both p53 and γH2AX levels were further elevated upon OH-ME treatment in *CSA*^*–/–*^ cells, which was particularly evident after 8 h and 24 h (Fig. S12A–C, middle panel). Similar, but slightly attenuated effects were detected in *XPA*^*–/–*^ cells after OH-ME exposure (Fig. S12A–C, right panel). As expected, UV irradiation resulted in earlier DDR signaling, which was also potentiated, particularly in *CSA*^–/–^ but also in *XPA*^–/–^ cells, compared to HCT116 CSB-mClover WT cells (Fig. S12A–C).

Subsequently, the proteasomal degradation of CSB following OH-ME treatment was analyzed in WT cells compared to cells deficient for *UVSSA*, which is a TC-NER factor known to counteract DNA damage-induced ubiquitination of CSB via the ubiquitin-specific protease USP7 [25]. While no CSB depletion was observed in WT cells treated with OH-ME, a strong decrease in CSB levels was detected in *UVSSA*^*–/–*^ cells (Fig. 6B), similar to UV irradiation. Importantly, CSB depletion in *UVSSA*^*–/–*^ cells was blocked by the proteasome inhibitor MG132, thus revealing that the UVSSA-USP7 complex counteracts the ubiquitin-mediated degradation of CSB after OH-ME treatment. No decrease in CSB was found in *CSA*^*–/–*^ cells exposed to OH-ME, whereas a moderate decrease was measured in *XPA*^*–/–*^ cells (Fig. S13A). These findings were independently confirmed in colony survival assays, in which *UVSSA*^–/–^ and *CSA*^–/–^ cells were highly sensitive towards OH-ME treatment, followed by *XPA*^–/–^ cells. In contrast to that, NER-proficient WT cells were resistant to OH-ME concentrations up to 100 µM (Fig. S13B).

As a next step, we measured the recovery of RNA synthesis to monitor TC-NER upon OH-ME-induced DNA damage. After treatment of HepG2 cells with 75 µM OH-ME, the medium was exchanged, and the cells were cultivated for a recovery time ranging from 0 to 24 h. EU incorporation was then measured by confocal microscopy as described above. We found a strongly reduced EU signal directly after pulse-treatment with 75 µM OH-ME (0 h), which further declined and reached a minimum after 2 h (Fig. 6C, D). Subsequently, the EU incorporation increased again and reached ~ 75% of that observed in control cells (Fig. 6C, D). Using the same experimental setup, the localization of the RNAPII subunit RPB1 and its phosphorylated form (pRPB1) was analyzed by cell fractionation and western blot detection. Directly after the treatment with OH-ME (0 h), little RPB1 was bound to chromatin as compared to solvent-treated control cells that displayed a strong RPB1 signal in the chromatin fraction (Figs. 6E and S13C). However, the chromatin-bound RPB1 level increased again with longer recovery time after OH-ME treatment (Figs. 6E and S13C). Concomitantly, RPB1 levels in the soluble fraction of OH-ME- treated cells initially declined and then increased even above basal levels in solvent- treated control cells (Figs. 6E and S13C). Similar effects were observed for pRPB1 levels in the soluble fraction and chromatin (Figs. 6E and S13D). Collectively, the data provided evidence that DNA adducts triggered by OH-ME block de novo RNA synthesis by stalling RNAPII, which then engages the canonical TC-NER pathway via CSB, CSA and UVSSA to restore DNA transcription.

### R-loop formation and chromosomal instability triggered by OH-ME

**Fig. 7:**
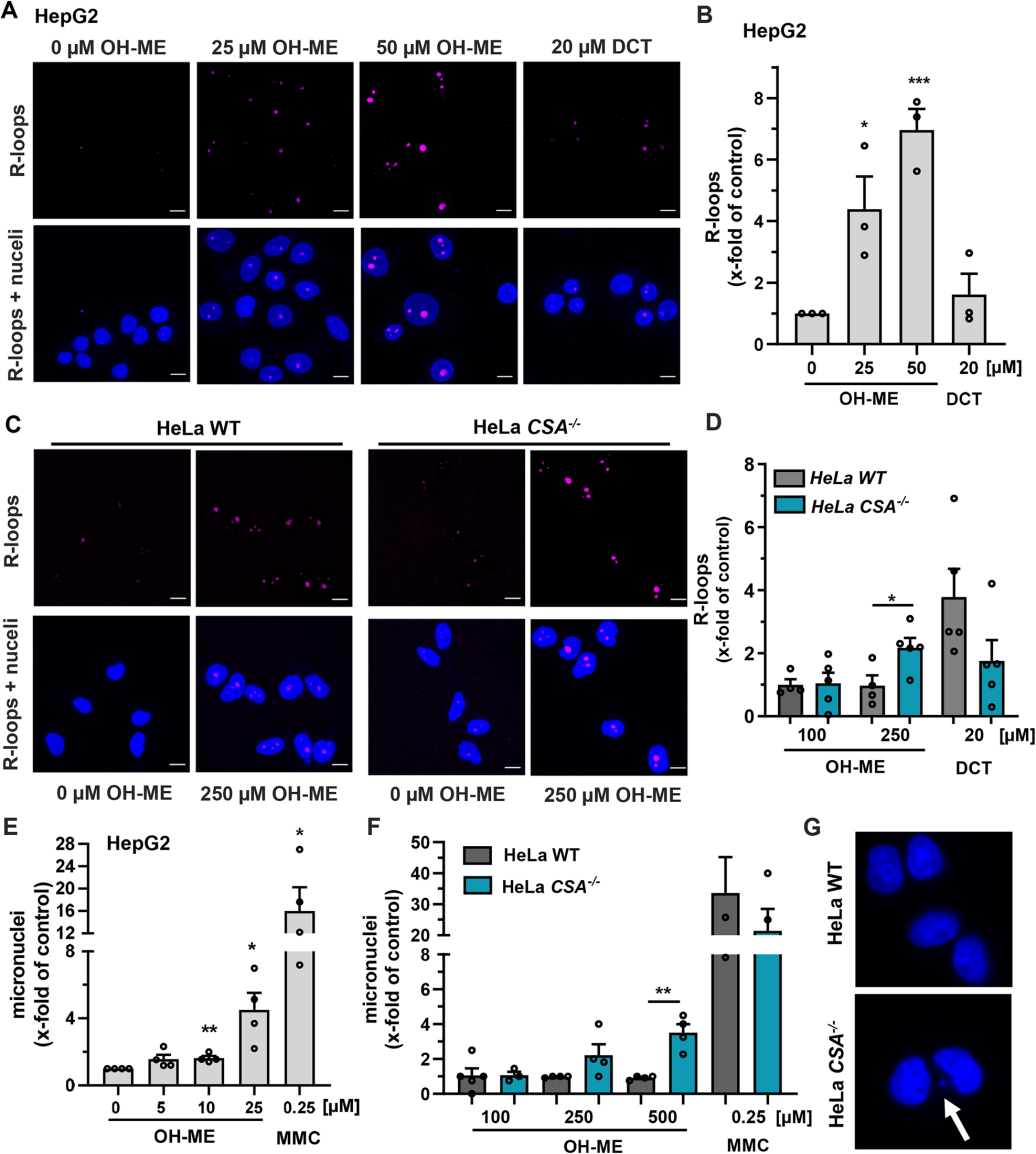
Impact of *CSA* deficiency on OH-ME-triggered genomic instability. Formation of R-Loops triggered by OH-ME in HepG2 (**A, B**) and HeLa WT as well as HeLa *CSA*^*–/–*^ cells (**C, D**). Cells were incubated with increasing concentrations of OH-ME or decitabine (DCT) as positive control for 48 h, processed and stained for R-Loop formation (pink). The nuclei were visualized by DAPI. Images were acquired by confocal microscopy and processed by Zen software. Representative images (**A, C**, scale bar: 10 µm) and the quantitative evaluation (**B, D**) are shown (*n* ≥ 4). Assessment of MN induction in HepG2 (**E**) and HeLa WT vs. HeLa *CSA*^*–/–*^ cells (**F**, **G**) after treatment with OH-ME over 24 h with a 72 h (HepG2) or 24 h (HeLa) recovery time (*n* = 4). Mitomycin C (MMC) served as a positive control, while DMSO was used as a solvent control. **G** Representative microscopic images. The white arrow indicates a micronucleus in a binucleated cell. All data are given as mean + SEM. *p < 0.05, **p < 0.01, ***p < 0.001.

Based on these findings, we speculated that the ME-derived DNA adducts may promote the formation of R-loops, as was previously shown with UV-induced DNA damage [26]. These are DNA: RNA hybrids formed between the nascent mRNA and the template strand in the upstream transcription bubble during transcription, which have regulatory functions, but are also associated with genome instability [27]. To this end, HepG2 cells were treated with OH-ME for 48 h and R-loop formation was assessed by confocal immunofluorescence microscopy using the R-loop-specific antibody S9.6 [28]. We observed a concentration-dependent increase in the R-loop level upon OH-ME exposure, with 50 µM OH-ME resulting in a 7-fold increase vs. control cells (Fig. 7A, B). The positive control decitabine, which is a cytosine analogue and DNA methylation inhibitor [29], caused a moderate 1.6-fold increase (Fig. 7A, B). To investigate the role of TC-NER in this process, HeLa WT and HeLa *CSA*^*–/–*^ were used and challenged with OH-ME. While almost no effect was detected in WT cells challenged with up to 250 µM OH-ME, an increased R-loop formation was observed in *CSA*^–/–^ cells treated with 250 µM OH-ME (Fig. 7C, D). As R-loop formation was linked to micronucleus (MN) formation and genomic instability [28], the clastogenicity of OH-ME was analyzed in HepG2 cells using the flow cytometry-based MN assay (Fig. S14A). Treatment with OH-ME for 24 h followed by medium exchange and subsequent recovery time of 72 h resulted in a concentration-dependent increase in MN formation, with a 4.5-fold induction at 25 µM OH-ME compared to the solvent control (Figs. 7E and S14B). The positive control MMC, which is a potent DNA crosslinking agent, increased MN levels by 16-fold. Cell viability at the test concentrations investigated for MN formation was always above 60% (Fig. S14C), excluding overt cytotoxicity. The same set of experiments was repeated in HeLa WT and *CSA*^–/–^ cells, however with a reduced recovery time of 24 h due to the faster proliferation in HeLa cells (Fig. S14A). No effect was seen in HeLa WT cells challenged with up to 500 µM OH-ME, whereas OH-ME induced a concentration-dependent formation of MN in HeLa *CSA*^*–/–*^ cells (Figs. 7F and S14D). As expected, treatment with the positive control MMC resulted in a strong increase both in HeLa WT and in HeLa *CSA*^–/–^ cells (Fig. 7F and Fig. S14D). Cell viability was above 70% in all treatment groups (Fig. S14E). These findings were corroborated using the cytokinesis-block MN assay, in which only MN formed in binucleated cells are measured. Importantly, no MN formation was detected in HeLa WT cells, whereas a concentration-dependent increase in MN was found in HeLa *CSA* k.o. cells (Fig. 7G and Fig. S14F), which is consistent with the flow cytometry-based MN determination. Collectively, these findings showed that ME-derived DNA adducts promote both R-loop and MN formation, which is augmented in cells deficient for TC-NER.

**Fig. 8:**
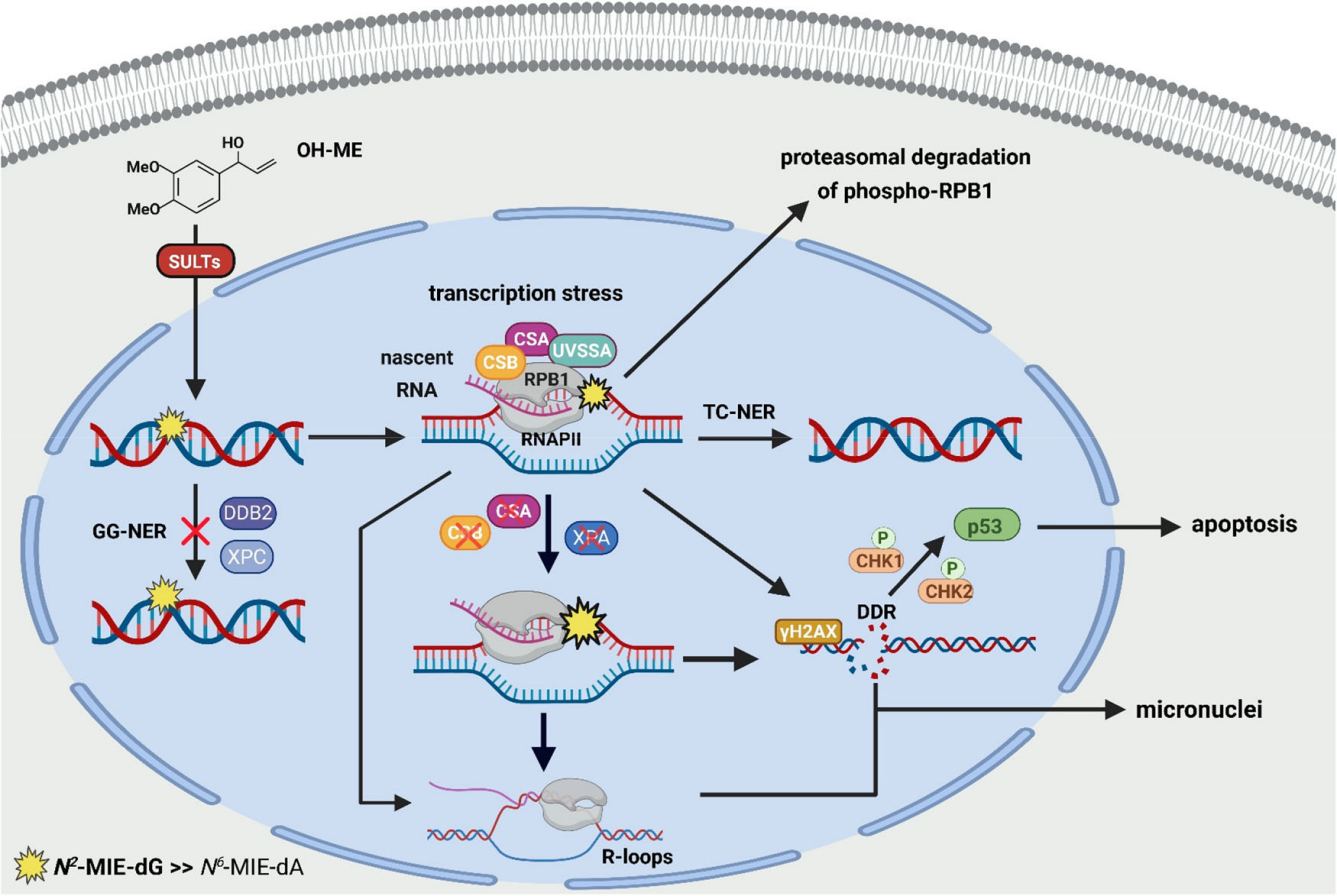
ME-derived DNA adducts, transcription stress and activation of canonical TC-NER to prevent genomic instability and cell death. OH-ME as main phase I metabolite of ME causes DNA adducts (*N*^2^-MIE-dG » *N*^6^-MIE-dA) upon metabolic activation by SULT enzymes. ME-derived DNA adducts are not subjected to GG-NER, but persist in the global genome. Adducts in the transcribed region of the genome stall RNA polymerase II (RNAPII), which results in immobilization of CSB followed by the recruitment of CSA and UVSSA. CSA promotes the ubiquitination and proteasomal degradation of CSB upon OH-ME treatment, whereas UVSSA counteracts this process via USP7. The RPB1 subunit of stalled RNAPII is released from chromatin and exported into the cytoplasm for proteasomal degradation. At low DNA adduct levels, the triggered canonical TC-NER pathway removes the lesions, preserves genome integrity and promotes cell survival. At high DNA adduct levels, persistent transcription stress provokes genomic instability (formation of R-loops and micronuclei) and induces apoptotic cell death. Cells deficient in TC-NER (*CSA*^*–/–*^ or *CSB*^–/–^) or in both NER subpathways (*XPA*^–/–^) are hypersensitive to ME-derived DNA adducts, resulting in a hyperactivated DNA damage response (DDR), increased cell death induction and genomic instability. Created in BioRender. Fahrer, J. (2026) https://BioRender.com/c09atmy.

Altogether, our data demonstrate that ME-derived DNA adducts trigger the canonical TC-NER pathway by stalling RNAPII, which results in adduct removal and cellular resistance at low DNA adduct levels. In turn, high adduct levels cause severe transcription stress, promoting genomic instability and cell death. In the absence of the essential TC-NER factors CSA and CSB, transcription stress by ME-derived DNA adducts is potentiated, culminating in genomic instability and cytotoxicity at much lower OH-ME concentrations. GG-NER, however, is not activated by ME-derived DNA adducts, thus facilitating DNA adduct persistence and accumulation in the global genome. The main findings are summarized in Fig. 8.

## Discussion

Using different cell models, our study showed that both ME-derived adducts, *N*^2^-MIE-dG and *N*^6^-MIE-dA, are only partially repaired and persist over time. This is in agreement with findings obtained for the structurally related compound ES in HepaRG cells and primary rat hepatocytes [20, 30], which can promote DNA adduct accumulation after repeated exposure [31]. The inefficient removal of *N*^2^-MIE-dG and *N*^6^-MIE-dA adducts observed in our study is very likely the explanation for the presence of the main adduct *N*^2^-MIE-dG in a vast majority of analyzed human liver biopsies [32]. Very recently, we have demonstrated that ES-derived *N*^2^-dG adducts also occur in a multitude of analyzed human liver samples at comparable levels [33]. To study the suggested role of NER in the removal of the ME-derived DNA adducts, the central NER factor XPA was downregulated in HepG2 cells by siRNA, which inactivates both GG-NER and TC-NER. Under these settings, OH-ME treatment strongly increased DDR markers, such as γH2AX, resulting in elevated cytotoxicity, which was also found in primary *XPA*^*–/–*^ hepatocytes and in HCT116 CSB-mClover cells deficient for *XPA*. These data strongly suggest that NER is involved in the removal of ME-derived DNA adducts. This is generally in line with the notion that bulky adducts caused by environmental carcinogens, such as BaP or 2-amino-1-methyl-6-phenylimidazo[4,5-b]pyridine (PhIP), are substrates of the NER pathway [4]. Notably, BaP causes mainly *N*^2^-dG adducts similar to OH-ME, whereas PhIP induces C8-dG adducts as major lesion [3, 34].

To detail the relevance of the NER subpathways, *i.e*., GG-NER and TC-NER, we used isogenic cell models proficient or deficient in DDB2 (GG-NER compromised) or CSA (TC-NER defective). DDB2 is together with DDB1 part of the UV-DDB complex that further comprises CUL4A/B and RBX1 [35]. This complex supports DNA damage recognition within the GG-NER subpathway, initially recognized as the complex required for sensing of the main UV-light-induced DNA damage, *i.e*., cyclobutane pyrimidine dimers (CPDs) [36]. CPDs only moderately affect base pairing and mildly destabilize the DNA helix and are therefore poor substrates for the main GG-NER initiating XPC-RAD23-CETN2 protein complex [37]. More recently, DDB2 was found to support GG-NER for other DNA lesions [38, 39] and DNA damage detection in the chromatin context together with XPC [40]. Knockout of *DDB2*, however, had no impact on OH-ME-triggered DDR, cell death induction and cell viability, thereby indicating that DDB2-mediated GG-NER is not involved. Strikingly, the highly similar CRL4^CSA^ complex, in which the substrate receptor is swapped for the essential TC-NER factor together with the ubiquitin ligase modulating protein DDA1 [41], was shown to be required for the resistance to OH-ME. *CSA*^–/–^ strongly increased the sensitivity of cells towards OH-ME in terms of DNA adduct accumulation, DDR activation and cell death induction. CSA is recruited via a CSA-binding motif located in the TC-NER initiating protein CSB [42], which is the first protein to bind to lesion-stalled RNAPII [24]. Intriguingly, also *CSB*^–/–^ cells were hypersensitive to OH-ME, as highlighted by the much lower EC_50_ values determined in *CSB*^–/–^ as compared to WT cells. Together with the only partial reduction of ME-derived DNA adduct levels by 30–40% after 24 h, these findings strongly suggest that only adducts in the transcribed DNA strand are recognized due to RNAPII stalling and repaired by TC-NER, whereas ME-derived lesions in the global genome are not properly detected and removed. The latter assumption is supported by a molecular modeling study of ES-derived *N*^2^-dG DNA adducts in double-stranded DNA, which revealed little distortion of the DNA helix and thus inefficient recognition by NER [20]. This very likely also holds true for the structurally related *N*^2^-MIE-dG adduct analyzed herein. Interestingly, resistance to GG-NER was described previously for other *N*^2^-dG lesions, e.g., caused by BaP. Following metabolic activation to BaP-diol epoxide, ( + )-*trans*-anti-BaPDE-*N*^2^-dG is formed as a major adduct [3]. This lesion was shown to be repair-resistant, since the bulky BaP moiety is accommodated into the DNA minor groove due to the external conformation of the adduct, thus causing little DNA distortion [43]. In contrast to that, the minor adduct (+)-*cis*-anti-BaPDE-*N*^2^-dG adopts an intercalative conformation with displacement of the modified base, thereby causing much stronger DNA distortion and better repair efficiency [43].

The concept that ME-triggered DNA adducts block RNAPII and are thereby subject to TC-NER-mediated repair was further corroborated by a strong decrease in de novo RNA transcription as revealed by an EU incorporation assay. This effect was observed both in the nucleoplasm as well as in the nucleoli, reflecting stalling of both RNAPII and RNAPI. This phenomenon is already known for UV-induced DNA lesions like CPDs that also block RNAPI and thereby shut down the transcription of ribosomal DNA (rDNA) [44, 45]. Importantly, TC-NER is required to repair UV-induced lesions in rDNA in a CSA, CSB and UVSSA-dependent manner [44]. Furthermore, ( + )-*trans*-anti-BaPDE-*N*^2^-dG stalled RNAPII as shown by an in vitro transcription assay with human nuclear extracts [46]. The OH-ME-mediated block of transcription was further increased in the absence of *CSA*, which is likely attributable to the prolonged stalling of the RNAPII/CSB complex due to the lack of CRL4^CSA^ -mediated CSB and RPB1 ubiquitination. These are required to resolve lesion-stalled RNAPII/CSB complexes, which allows NER to continue [24]. Indeed, we demonstrated by FRAP analysis that CSB was moderately immobilized on chromatin in response to OH-ME treatment. In this regard, it is also noteworthy to mention that CSB promotes the forward translocation of RNAPII [47]. If this is hindered, e.g., by UV-induced DNA lesions, the interaction of CSB and RNAPII is stabilized, leading to the immobilization of CSB on chromatin [23, 48, 49], as observed here for OH-ME as well. Importantly, this CSB immobilization was strongly potentiated in *CSA*^–/–^ cells, which is perfectly in line with the strongly increased transcription inhibition upon OH-ME treatment in *CSA*^–/–^ cells as compared to WT cells.

In addition to that, we showed that *XPC* knockdown had no influence on CSB immobilization, further supporting the conclusion that GG-NER is not involved in DNA adduct removal upon OH-ME exposure. Furthermore, we analyzed the fate of CSB in the presence and absence of the proteasome inhibitor MG132 in WT cells and cells deficient for *UVSSA*, *CSA* and *XPA*. Upon UV CRL4^CSA^ ubiquitylates both CSB and RPB1, required for efficient TC-NER progression. Ubiquitination of CSB, and its consequent proteasomal degradation, is counteracted by the TC-NER factor UVSSA that is recruited to the stalled RNAPII/CSB/CSA complex. UVSSA partners with the broad deubiquitinating enzyme USP7, which removes ubiquitin moieties from CSB and thereby prevents its proteasomal degradation [25, 50]. Our experiments revealed that CSB is degraded by the proteasome in the absence of UVSSA, implying that UVSSA is crucial to counteract CRL4^CSA^-dependent CSB degradation in response to OH-ME-induced DNA adducts, similarly to UV-induced DNA lesions. As expected, no CSB degradation was observed in *CSA*^–/–^ cells, consistent with the notion that CRL4^CSA^ is required for ubiquitin-mediated CSB degradation. Furthermore, we showed that RNAPII is released from chromatin following OH-ME exposure, which then recovered over 24 h. This observation is in line with the recovery of RNA synthesis after 24 h, as shown by the EU incorporation assay. This is very likely explained by polyubiquitination of the RNAPII subunit RPB1, which also occurs upon UV-induced transcription blocks [51]. TC-NER-mediated ubiquitination of RPB1 enables either backtracking, removal or degradation of lesion-stalled RNAPII, which is required for exposing the transcription-blocking lesion and subsequent removal by the downstream NER machinery [5]. Intriguingly, we observed nuclear depletion of RPB1 as well as phospho-Ser2/Ser5-RPB1 following exposure to OH-ME, while at the same time, RPB1 appeared in the cytoplasm. Proteasome inhibition by MG132 blocked RPB1 degradation and caused accumulation of Ser2/5-phosphorylated RPB1 in the cytoplasm. This is consistent with the notion that, upon persistent stalling of RNAPII, its active subunit RPB1 (phosphorylated at both Ser2 and Ser5) undergoes ubiquitylation at Lys-1268 followed by its proteasomal degradation [52]. Altogether, these findings led to the conclusion that the transcription-blocking lesions induced by OH-ME trigger the canonical TC-NER pathway, requiring CSA, CSB and UVSSA.

Finally, we showed that OH-ME-triggered DNA adducts give rise to R-loops, which were potentiated in the absence of *CSA*, and cause MN formation as markers of genomic instability. The formed R-loops may then collide with replication forks, potentially giving rise to DNA double-strand breaks, which are clastogenic and cause MN, as demonstrated in HepG2 cells. In this context, it should be mentioned that OH-ME-triggered replication stress was demonstrated previously by our group [15], fostering this hypothesis. In HeLa WT cells, fewer adducts are generated than in HepG2 upon OH-ME exposure, thus causing only moderate RNAPII stalling and no or little R-loop formation. In contrast to that, higher levels of ME-derived DNA adducts arise in HeLa *CSA*^–/–^ cells due to the lack of TC-NER and thus provoke RNAPII stalling. This persistent transcription stress increased the R-loop formation, which results in DNA double-strand breaks upon replication and MN, as shown herein. These observations fit to the notion that stalled RNAPII promotes R-loop formation, which can result in genomic instability if not resolved [53]. Moreover, a recent study showed that BaP, which mainly induces *N*^2^-dG lesions (see above), also triggers R-loop formation and genomic instability [54], which nicely mirrors the effects of OH-ME-induced *N*^2^-dG adducts.

The findings of our study are important for CS patients due to the probable dietary exposure to ME and structurally related phenylpropenes like ES. Cells from CS patients are prone to undergo cell death already at low levels of transcription-blocking DNA lesions induced by UV irradiation [55]. In CS patients, transcription stress driven by various endogenous and exogenous sources of DNA damage can trigger cell death in different postmitotic tissues, thereby causing premature organ atrophy and functional decline [5]. Liver dysfunction with elevated transaminase levels was reported in the majority of CS patients in a cohort study [56]. This is substantiated by a very recent study in eight pediatric patients with CSA-related CS, all of whom display persistently elevated liver transaminase levels [57]. It is thus conceivable that chronic intake of dietary phenylpropenes, such as ME and ES, could exacerbate liver dysfunction in CS patients or might even cause acute liver injury. Given the persistence of ME-derived DNA adducts shown in our present study, repetitive exposure will very likely result in DNA adduct accumulation both in the non-transcribed and transcribed regions of the genome. In CS patients, ME-derived adducts are expected to accumulate much faster due to the lack of TC-NER and could then induce toxicity in the liver or other organs, such as the lung or kidney. Notably, *N*^2^-MIE-dG adducts have not only been detected in the liver, but also in other organs, such as the large intestine and the kidney, which was demonstrated in mice upon oral administration of ME [58]. In DNA repair proficient and proliferating cells, high ME- or ES-derived DNA adduct levels are required to trigger cytotoxicity as shown previously [15, 59]. This comparably low sensitivity is likely attributable to the efficient bypass of these adducts by DNA translesion synthesis, which was reported for *N*^2^-MIE-dG and the ES-derived adduct *N*^2^-IES-dG in vitro [60]. The study further showed that recombinant pol κ and pol η bypass the adducts in an error-free manner, which has been extended to the *N*^6^-MIE-dA adduct [61].

Collectively, our results demonstrate that TC-NER is crucial for the repair of ME-induced DNA adducts and protects cells against detrimental transcription stress, which is of particular relevance for CS patients with hereditary TC-NER defects.

## Material and methods

### Cell culture

HepG2 cells were obtained from DSMZ (Braunschweig, Germany). HeLa wildtype (WT) cells were also purchased at DMSZ and used to generate HeLa *CSA*^*–/–*^ as well as HeLa *DDB2*^*–/–*^ as described previously [62]. The gene knockouts were authenticated by PCR and western blot analysis. In the case of HeLa *CSA*^*–/–*^, TC-NER deficiency was independently confirmed by the host cell reactivation assay [62]. HCT116 WT, HCT116 *CSB*^*–/–*^ and HCT116 *CSA*^*–/–*^ were engineered as described and authentication was performed by immunoblotting or genomic PCR [41]. HCT116 CSB-mClover WT, HCT116 CSB-mClover *CSA*^–/–^, HCT116 CSB-mClover *XPA*^–/–^ and HCT116 CSB-mClover *UVSSA*^–/–^ cells were engineered and authenticated as described [23]. All cell lines were maintained in DMEM high glucose supplemented with 10% fetal calf serum (FCS) and antibiotics (100 U/mL penicillin and 100 µg/mL streptomycin). The cell lines were cultivated at 37 °C in a humidified atmosphere of 5% CO_2_. Cell culture medium and antibiotics were purchased from Gibco Life Technologies (Waltham, MA, USA), while FCS was supplied by PAN-Biotech (Aidenbach, Germany). Cells were mycoplasma negative as routinely demonstrated by PCR using the Venor®GeM Classic kit (Minerva Biolabs, Berlin, Germany). All cell models were re-authenticated on the protein level and by measuring their differential response to cisplatin and UV-C radiation.

### Chemicals and cell treatment

1′-Hydroxymethyleugenol (OH-ME) was synthesized as described [13], and the degree of purity was confirmed using ^1^H-NMR. A stock solution with a concentration of 1 M in dimethylsulfoxide (DMSO) was prepared, which was further diluted in DMSO and then added to the cell culture medium to reach the final test concentrations as indicated. The test concentrations were selected based on previous work [15]. DMSO (VWR International, Darmstadt, Germany) was used as solvent control and was 0.1% for all treatments. Different positive controls were used depending on the endpoint being investigated: etoposide (Hycultec, Beutelsbach, Germany), mitomycin C (MMC) (Sigma-Aldrich, St. Louis, MO, USA), saponin (Serva, Heidelberg, Germany), actinomycin D (Carl Roth, Karlsruhe, Germany), decitabine (Hycultec, Beutelsbach, Germany) and cisplatin (CisPT) (Hycultec, Beutelsbach, Germany). In addition, a Biometra FLX-20M apparatus was used for UV-C irradiation (Analytik Jena, Jena, Germany).

### Gene knockdown with siRNA transfection

Knockdown of *XPA* in HepG2 was performed as described previously [15] and confirmed by western blot analysis. HCT116 CSB-mClover cells were seeded on glass coverslips 1 day before transfection and 3 days before imaging. Transient knockdown was performed using Lipofectamine RNAiMAX (Thermo Fisher Scientific, Darmstadt, Germany) following the manufacturer’s instructions. CTRL (5′-UGGUUUACAUGUUGUGUGA-3′) and *XPC* (5′-GCAAAUGGCUUCUAUCGAA-3′) siRNAs (both from Revvity, Waltham, USA) were used. Knockdown was validated previously [63].

### Isolation of primary mouse hepatocytes

*XPA* knockout mice were generated as reported [64] and provided by Dr. Harry van Steeg (Leiden University Medical Center, The Netherlands). C57BL/6 Wildtype (WT) and *XPA*-deficient mice (typical age: 8–16 weeks) of both sexes were obtained from our in-house animal facility. Following anesthesia by *i.p*. administration of pentobarbital, primary mouse hepatocytes (PMH) were isolated in a two-stage EGTA/collagenase perfusion as previously described [65]. Cell viability was monitored by using trypan blue and was ≥ 90% for all experiments. PMH were seeded in collagen-coated cell culture plates, allowed to adhere for 3 h and then treated with OH-ME or the solvent control DMSO.

### Isolation of genomic DNA, DNA digestion and analysis of OH-ME-triggered DNA adducts by mass spectrometry (MS)

Isolation of genomic DNA, enzymatic digestion and MS measurements were performed as previously described [15]. In brief, DNA was isolated by chloroform/phenol extraction. The concentration and purity of the isolated DNA were determined using the Spark® microplate reader (Tecan, Männedorf, Switzerland). Enzymatic digestion into nucleosides was performed with 30 µg DNA per sample and spiked with ^*15*^*N*-labeled adduct standards (2 nM) as well as ^*15*^*N*_*5*_-dG (20 µM). The DNA adduct standards were synthesized, purified and characterized as reported [12]. The mass spectrometry-based measurements of the *N*^2^-MIE-dG and *N*^6^-MIE-dA adducts were performed on an Agilent 1290 Infinity UHPLC system (Agilent, Santa Clara, CA, USA) coupled with a Sciex QTrap 5500 MS (Sciex, Marlborough, MA, USA) as described [15]. Electrospray ionization (ESI) in positive mode was used for MS measurements, applying the MRM technique. The compound-specific mass spectrometric parameters can be found in the SI section (Table S1). In addition, the dG content of all samples was determined, and the adduct levels were normalized to the overall nucleoside content as reported [15].

### Preparation of cell lysates and Western blot analysis

Cells grown overnight were treated with OH-ME as indicated. The cells were then harvested directly in 1x Laemmli buffer (40 mM Tris-HCl, pH 6.8, 1.6% SDS, 8% glycerol, 0.016% bromophenol blue, and 0.8% β-mercaptoethanol). Protein expression levels were analyzed by SDS-PAGE and western blot detection essentially as described [66]. The proteins were visualized using the Western Lightning Plus-ECL reagent (Perkin Elmer, Waltham, MA, USA) and the c300 chemiluminescence imager (Azure Biosystems, Dublin, CA, USA). Quantitative evaluation was performed by applying the software AzureSpot 2.0.062 (Azure Biosystems, Dublin, CA, USA) as previously described [67]. The signal intensities of all investigated protein bands were quantified, normalized to the loading control and then related to the negative control. The primary and secondary antibodies used are listed in supplementary Table S2.

### Chromatin retention assay

Cell fractionation and chromatin isolation were performed as described elsewhere [67]. To this end, cells were grown overnight and treated with OH-ME for 16 h. Subsequently, the medium was exchanged, and the cells were maintained in fresh medium for up to 24 h. The cells were harvested, resuspended in lysis buffer (150 mM KCl, 2.5 mM MgCl_2_, 50 mM HEPES pH 7.8, 5 mM EDTA pH 8, 3 mM DTT, 10% glycerol, 0.5% Triton X-100 and freshly added protease inhibitor cocktail) and incubated on ice for 15 min. Following centrifugation for 15 min at 16,000 × *g* and 4 °C, the chromatin-bound fraction was collected, while the supernatant (soluble fraction) was transferred to a new reaction tube. The chromatin-bound fraction was washed two times in lysis buffer and sonicated for 3 min. Both fractions were mixed with 5x Laemmli buffer, heated to 95 °C for 10 min and subjected to SDS-PAGE and western blot analysis as described.

### Analysis of cell viability by MTS and resazurin reduction assay

To determine cell viability, cells were seeded in 96-well format and treated for 48 or 72 h with increasing concentrations of OH-ME. Cell viability was assessed using the CellTiter 96® Aqueous OneSolution Cell Proliferation Assay (Promega, Mannheim, Germany) on a microplate reader, as specified by the manufacturer and described previously [68]. Furthermore, cell viability was measured using the resazurin reduction assay as reported recently [59]. To this end, the fluorescence was measured on a Spark® microplate reader (Tecan, Männedorf, Switzerland) with an excitation at 544 nm and emission at 590 nm. DMSO served as the negative control, and saponin as the positive control. The EC_50_ values were determined using GraphPad Prism 9.0 software (GraphPad Software Inc., Boston, MA, USA) by fitting the data with a sigmoidal, non-linear function as described before [11].

### Colony survival

750 cells were seeded in 6-well plates in triplicate and were either non-treated or treated with 5, 10, 20, 40, 60, 80 or 100 μM OH-ME 48 h after seeding. 7–10 days post-treatment, the cells were fixed and stained with 50% ethanol, 10% acetic acid, and 0.2% Brilliant Blue R. After colony counting using GelCount (Oxford Optronix, Adderbury, UK), each cell line was normalized to the non-treated condition, which was set to 100% survival.

### Analysis of cell death induction by Annexin V-FITC/PI staining and flow cytometry

The assessment of cell death induction was performed using the flow cytometric Annexin V-FITC/PI assay as previously described [69]. Cells grown in 3.5 cm dishes were exposed to OH-ME for 48 h as indicated. Attached and detached cells were harvested as cell pellets, washed with PBS and then resuspended in binding buffer with Annexin V-FITC (5% dye in 10 mM HEPES pH 7.4, 140 mM NaCl, 2.5 mM CaCl_2_, 0.1% BSA) (Miltenyi Biotec, Bergisch Gladbach, Germany) and incubated on ice for 15 min. Propidium iodide (PI; 50 µg/ml) (Carl Roth, Karlsruhe, Germany) was then added, and the samples were analyzed by flow cytometry using either a BD Accuri™ C6 (BD Biosciences, Heidelberg, Germany) or a BD FACS Canto II (BD Biosciences, Heidelberg, Germany). Viable (FITC negative, PI negative), early apoptotic (FITC positive, PI negative) and late apoptotic/necrotic (FITC positive, PI positive) cell populations were determined by gating with either BD Accuri^TM^ C6 Software or BD FACSDiva software 6.0 using negative and positive controls.

### Assessment of subG1 population

The subG1 population serves as a marker for cell death induction and was analyzed as previously described [70]. The cells were treated for 48 h, harvested and incubated at −20 °C in 70% ethanol for at least one day. After centrifugation, the cell pellets were resuspended in PBS containing RNase A (1 µg/µl) and incubated for one hour. After staining with PI (50 µg/ml), the samples were measured by flow cytometry on a BD FACS Canto II. The data were gated according to the intensity of the PI signal in the different phases of the cell cycle and subsequently analyzed using the BD FACSDiva software 6.0.

### Assessment of de novo transcription using EU incorporation

The analysis of EU incorporation using click chemistry was carried out as previously described with slight modifications [71]. Briefly, the cells were seeded on coverslips, grown overnight and then treated with OH-ME for 24 h. DMSO and actinomycin D were included as solvent and positive control. 2 h before the end of the incubation, 100 µM EU was added to the cell culture medium. Subsequently, the medium was aspirated, and cells were washed twice with PBS, followed by fixation at −20 °C in 100% methanol for 15 min. After blocking with 5% BSA in PBS/0.3% Triton X-100 at RT for 1 h, the EU reaction cocktail consisting of 4.98 mM aminoguanidine hydrochloride, 5.68 mM ascorbic acid, 1 mM CuSO_4_ and 50 µM FAMazide (Lumiprobe GmbH, Hannover, Germany) in PBS/0.3% Triton X-100 was added and incubated for 1 h at RT in the dark. Finally, cells were washed three times with PBS before embedding in Vectashield® with DAPI (Vector Labs, Burlingame, CA, USA). The samples were analyzed by confocal microscopy using a Zeiss Axio Observer 7 microscope equipped with a 63x oil immersion objective (Plan-Apochromat 63x/1.40 DIC M27) and the LSM 900 confocal laser scanner (Carl Zeiss, Oberkochen, Germany). Images were acquired as optical sections using Zen Software 3.4 (Carl Zeiss, Jena, Germany). Images were processed, and quantitative evaluation was carried out using Fiji software (ImageJ). The integrated density of the EU signals of the individual images was measured, which was then related to the negative control.

### Assessment of CSB protein levels by fluorescent microscopy

The fluorescent signal derived from the mClover tag fused to CSB was used to measure relative CSB levels after exposure to either OH-ME or UV-light. HCT116 WT and CSB-mClover cell lines (WT, *UVSSA*^–/–^, *CSA*^–/–^, or *XPA*^–/–^) were grown on coverslips for 24 h. Cells were then treated with DMSO, 25 μM OH-ME, or with a combination of 25 μM OH-ME and 2 μM MG132, for 16 h. As a control, cells were exposed to 4 J/m² UV-C irradiation and allowed to recover for 16 h. Cells were fixed in 4% formaldehyde in PBS, permeabilized with 0.1% Triton X-100 in PBS, and counterstained with DAPI. Following repeated washes with 0.1% Triton X-100 in PBS and PBS alone, coverslips were mounted using Aqua-Poly/Mount (Polysciences Europe GmbH, Hirschberg, Germany). Images were acquired using an LSM700 confocal microscope equipped with a 40× Plan-Apochromat 1.3 NA oil-immersion objective (Carl Zeiss, Oberkochen, Germany). Fluorescence intensities were quantified in Fiji using a custom ImageJ macro. Nuclear CSB-mClover signals were background-corrected against HCT116 WT cells and normalized to untreated controls (set to 1).

### Live cell imaging

FRAP experiments were performed as previously described [23]. In short, cells were imaged on a Leica SP8 confocal microscope with a 40×/1.25 NA oil lens at 37°C and 5% CO₂. HCT116 CSB-mClover, *CSA*^–/–^, and *XPA*^–/–^ cells were seeded on glass coverslips 2 days before imaging and treated with 25 µM OH-ME for 16 h prior to imaging. OH-ME treatment was maintained during acquisition. CSB-mClover mobility was monitored every 200 ms at 400 Hz with 8x zoom in a strip of 512 × 16 pixels across the nucleus until steady state (pre-bleach), then photobleached at maximum laser power, and recovery was recorded every 200 ms at low intensity (post-bleach). Fluorescence was background-corrected, normalized to the last 30 pre-bleach frames, and set to 100%. At least 30 cells were analyzed per condition. The immobile fraction (F_imm_) was calculated as follows: F_imm_ = 1–(I_final, treat_–I_0, treat_)/(I_final, untr_–I_0, treat_).

### Analysis of (phospho-)RPB1 by confocal microscopy

Cells were grown on coverslips and treated for 24 h with OH-ME or the solvent control DMSO as indicated. The proteasomal inhibitor MG132 (10 µM) was added 6 h prior to the end of the incubation. Subsequently, the cells were fixed with 100% methanol at –20 °C for 15 min and then blocked with 5% BSA in PBS/0.3% Triton X-100 at RT for 1 h. Incubation with the primary antibodies RPB1 and pRPB1, respectively, (1:1000 in PBS/0.3% Triton X-100) was performed overnight at 4 °C. Afterward, the coverslips were washed with PBS with or without 0.4 M NaCl before incubation with the corresponding Alexa Fluor 647-conjugated goat-anti-mouse and Alexa Fluor 488-conjugated goat-anti-rabbit secondary antibody (each 1:400 in PBS/0.3% Triton X-100) for 1 h at RT. After further washing steps, the cells were embedded with Vectashield® containing DAPI. The samples were analyzed by confocal microscopy as described above for the EU measurements. The quantitative image analysis was performed using Fiji software (ImageJ). The integrated density of the pRRB1 and RPB1 signals in the nucleus and the cytoplasm of the individual images was measured, which was then related to the corresponding negative control.

### Determination of R-Loop formation

Cells seeded on coverslips were grown overnight before treatment with OH-ME and decitabine as a positive control for 48 h. R-Loop formation was analyzed by confocal immunofluorescence microscopy as described [28]. To this end, the cells were fixed with 100% methanol for 10 min followed by 100% acetone for 1 min at –20 °C. Subsequently, the cells were permeabilized by incubation with 0.05% Triton X-100 at RT for 15 min. After blocking for 1 h with 10% BSA, the primary antibody S9.6 was incubated overnight at 4 °C (1:200 in blocking solution). The coverslips were then washed with PBS and incubated for 1 h with the corresponding goat-anti-rabbit secondary antibody coupled to AlexaFluor 488 (1:400 in PBS). After another washing step, the cells were embedded with Vectashield® containing DAPI. Z-stack images were acquired by confocal microscopy as described for the EU measurements. All images were processed using Fiji software (ImageJ). Analysis was performed by determining the integrated density of the S9.6 signal, followed by normalization to the negative control.

### Flow cytometry-based in vitro micronucleus assay

The flow cytometry-based micronucleus assay was performed as previously described [59]. Cells were seeded in 3.5 or 6 cm dishes and allowed to attach overnight. After treatment for 24 h with OH-ME, the medium was removed, cells were washed with PBS and cultivated in fresh medium for another 24 h (HeLa) or 72 h (HepG2). DMSO served as the negative control and MMC as the positive control. The cells were then harvested, washed with PBS and resuspended in PBS containing 2% FCS and 125 µg/mL ethidium bromide monoazide (EMA) (Biotium, Fremont, CA, USA). The fluorescent dye was activated by photolysis for 30 min. Unbound dye was removed by the addition of PBS and centrifugation before the cells were incubated for 1 h in lysis buffer consisting of 0.584 mg/mL NaCl, 1 mg/mL sodium citrate, 0.3 μL/mL NP40, 1 mg/mL RNase A, as well as 0.2 μM SYTOX^™^ Green (Thermo Fisher, Waltham, MA, USA). The second lysis buffer (85.6 mg/mL sucrose, 15 mg/mL citric acid and 0.2 μM SYTOX^™^ Green) was added, and the cells were incubated overnight at 4 °C. Cells were analyzed with the BD FACS Canto II using the BD FACSDiva software, and gating was carried out as reported [59].

### Cytochalasin B micronucleus assay

The micronucleus assay with cytokinesis block was essentially performed as described [59]. The cells were seeded on coverslips, allowed to grow overnight and treated with OH-ME for 24 h. MMC served as a positive control, whereas DMSO was included as a solvent control. After the incubation period, the medium was removed, and fresh medium containing 6 µg/mL of the cytokinesis inhibitor cytochalasin B (Hycultec, Beutelsbach, Germany) was added for another 24 h. The cells were then washed with PBS, fixed with ice-cold methanol for 10 min, washed again and embedded in Vectashield® containing DAPI. The samples were analyzed with a Zeiss Axio Observer 7 microscope (Carl Zeiss, Jena, Germany) equipped with a 63x oil immersion objective and an Axiocam 305 mono using ZEN software 3.2. For each sample, at least 500 binucleated cells were analyzed for micronucleus formation.

### Statistical analysis

Experiments were performed at least three times independently, except where otherwise stated. Results from representative experiments are shown. Data values underwent Grubbs’ test to exclude outliers with a significance level of 0.05. Data are displayed as mean, and error bars represent standard error of the mean (SEM) using the GraphPad Prism 10.6.1 Software (GraphPad Software Inc.). Statistical analysis was performed using an unpaired, two-sided Student’s *t* test with Welch correction, and statistical significance was defined as *p* < 0.05. FRAP data were assessed using multiple nested unpaired two-tailed t-tests with a significance threshold of 0.05. For CSB IF data, statistical significance was determined using nested one-way ANOVA with Šídák’s multiple comparisons test.

## Supplementary information

Supplementary Information

Western Blot raw data

## Data Availability

The data generated during this study were included in the article and its supplementary files. They are also available from the corresponding author upon reasonable request.
