# Supplementary material for: Transcription-coupled nucleotide excision repair protects against genomic instability and cell death induced by the liver toxin methyleugenol
Source: Cell Death Dis. 2026 May 19;17(1):483. doi: 10.1038/s41419-026-08853-4 (PMC13186962; doi:10.1038/s41419-026-08853-4)
Supplement: Supplementary file 1 — Supplementary Information [file 41419_2026_8853_MOESM1_ESM.docx]

**Supplementary Information**

**Transcription-coupled nucleotide excision repair protects against genomic instability and cell death induced by the liver toxin methyleugenol**

Caroline Quarz^1^, Riccarda S. Walter^1^, Lydia Hens^1^, Max J. Carlsson^1^, Anastasia S. Vollmer^2,3^, Diana A. Llerena Schiffmacher^4^, Nina Pätzold^1^, Gabriel Ackermann^1^, Daniel Heylmann^2^, Simone Stegmüller^1^, Mohammed Meabed^5^, Alexander T. Cartus^1^, Ivano Amelio^6^, Elke Richling^1^, Wim Vermeulen^4^, Alex Pines^4^, Andriy Khobta^5^ and Jörg Fahrer^1^

^1^Division of Food Chemistry and Toxicology, Department of Chemistry, Rheinland-Pfälzische Technische Universität (RPTU) Kaiserslautern-Landau, Kaiserslautern, Germany

^2^Rudolf-Buchheim-Institute of Pharmacology, Biomedical Research Center, Justus Liebig University Giessen, Germany

^3^Department of Dermatology, University Medical Center, Heidelberg, Germany

^4^Department of Molecular Genetics, Erasmus MC, Rotterdam, Netherlands

^5^Department of Nutritional Toxicology, Friedrich Schiller University Jena, Germany

^6^Chair for Systems Toxicology, University of Konstanz, Konstanz, Germany

**CORRESPONDENCE**:

Jörg Fahrer, PhD, Division of Food Chemistry and Toxicology, Department of Chemistry, Rheinland-Pfälzische Technische Universität (RPTU) Kaiserslautern-Landau, Erwin-Schroedinger-Str. 52, D-67663 Kaiserslautern, Germany. Phone: + 49 631/2052974; email: joerg.fahrer@chem.rptu.de

**Supplementary Figures**

**
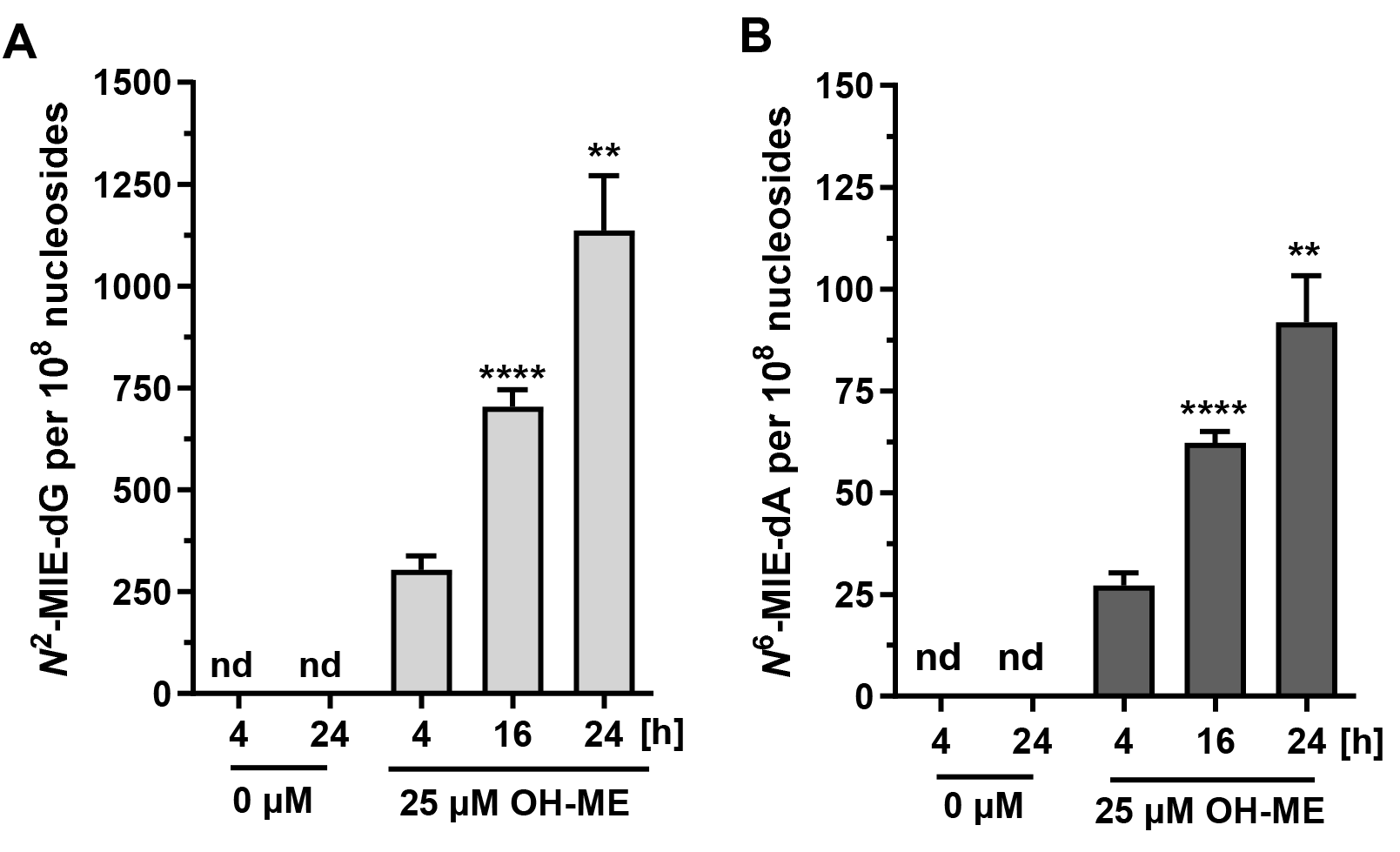
Figure S1**

**Figure S1: Time-dependent DNA adduct formation in HepG2 cells after OH-ME treatment. A** and **B** Formation of N^2^-MIE-dG and N^6^-MIE-dA adducts in human HepG2 cells. The cells were treated with 25 µM OH-ME for up to 24 h or exposed to solvent control (0 µM) and then harvest. Genomic DNA was isolated, digested to nucleosides and adduct levels were measured using stable isotope dilution analysis and UHPLC-mass spectrometry (n=5). All data given as mean + SEM; nd: not detected. Statistical analysis was performed using unpaired, two-sided t-test versus 4 h OH-ME (**p < 0.01, ****p<0.0001).

**Figure S2**

**
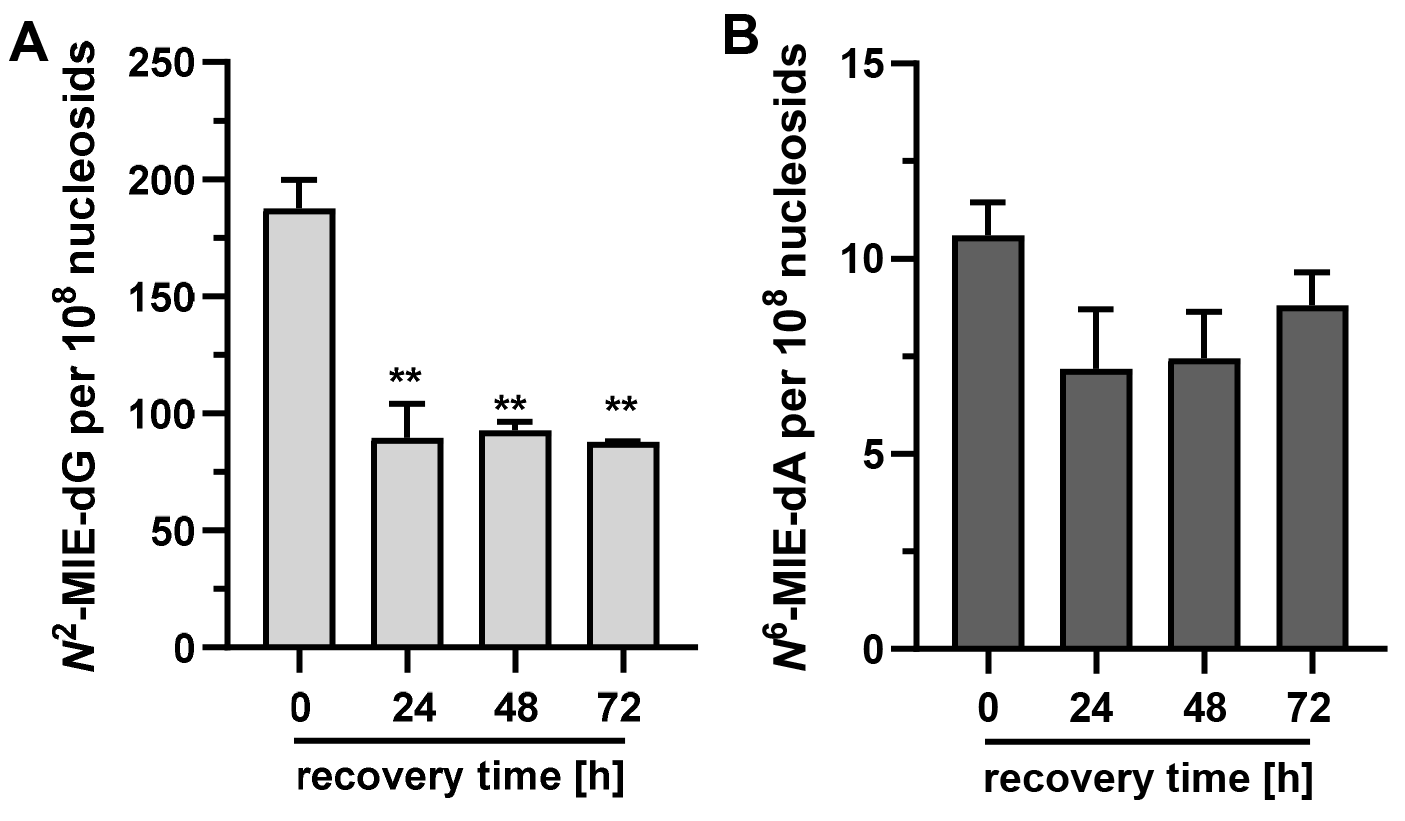
**

**Figure S2: DNA adduct formation and persistence in HepG2 cells after OH-ME treatment and recovery time. A** and **B** Formation of N^2^-MIE-dG and N^6^-MIE-dA adducts in human HepG2 cells. The cells were pulse-treated with 2.5 µM OH-ME for 8 h. After medium exchange, the cells were further cultivated for up to 72 h in fresh medium with reduced FBS content. Genomic DNA was isolated, digested to nucleosides and adduct levels were measured using stable isotope dilution analysis and UHPLC-mass spectrometry (n=4). All data given as mean + SEM. Statistical analysis was performed using unpaired, two-sided t-test versus 0 h (not significant p>0.05, **p < 0.01).

**
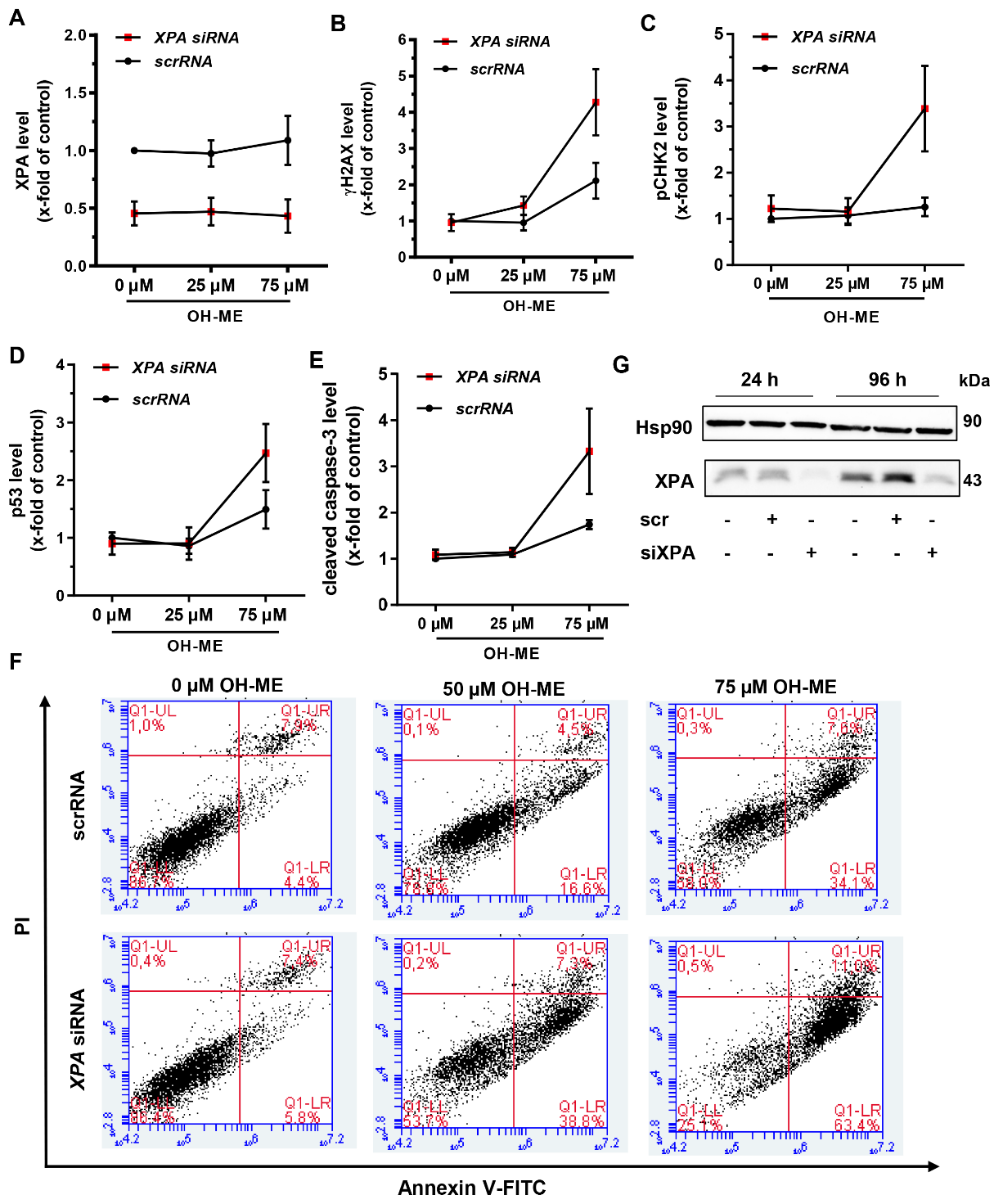
Figure S3:**

**Figure S3: Role of XPA-mediated NER in DNA damage response triggered by OH-ME. A – E** HepG2 cells were transfected with *XPA* siRNA or scrambled (scr) RNA followed by OH-ME treatment (0-75 µM) for 24 h. The samples were analyzed by SDS-PAGE and western blot detection of XPA, γH2AX, pCHK2, p53 and cleaved caspase-3. Hsp90 served as loading control. The densitometric evaluation of XPA **(A)**, γH2AX **(B)**, pCHK2 **(C)**, p53 **(D)** and cleaved caspase-3 **(E)** is shown (n=4). **F** Cell death induction after *XPA* knockdown and OH-ME treatment for 72 h in HepG2 cells. Apoptotic and necrotic cell death was determined by Annexin V-FITC/PI staining and flow cytometry. Representative dot plots and gating are shown. **G** Knockdown efficiency of *XPA* in HepG2 cells after 24 h and 96 h analyzed by SDS-PAGE and western blot detection. Hsp90 served as loading control.

**Figure S4**

**
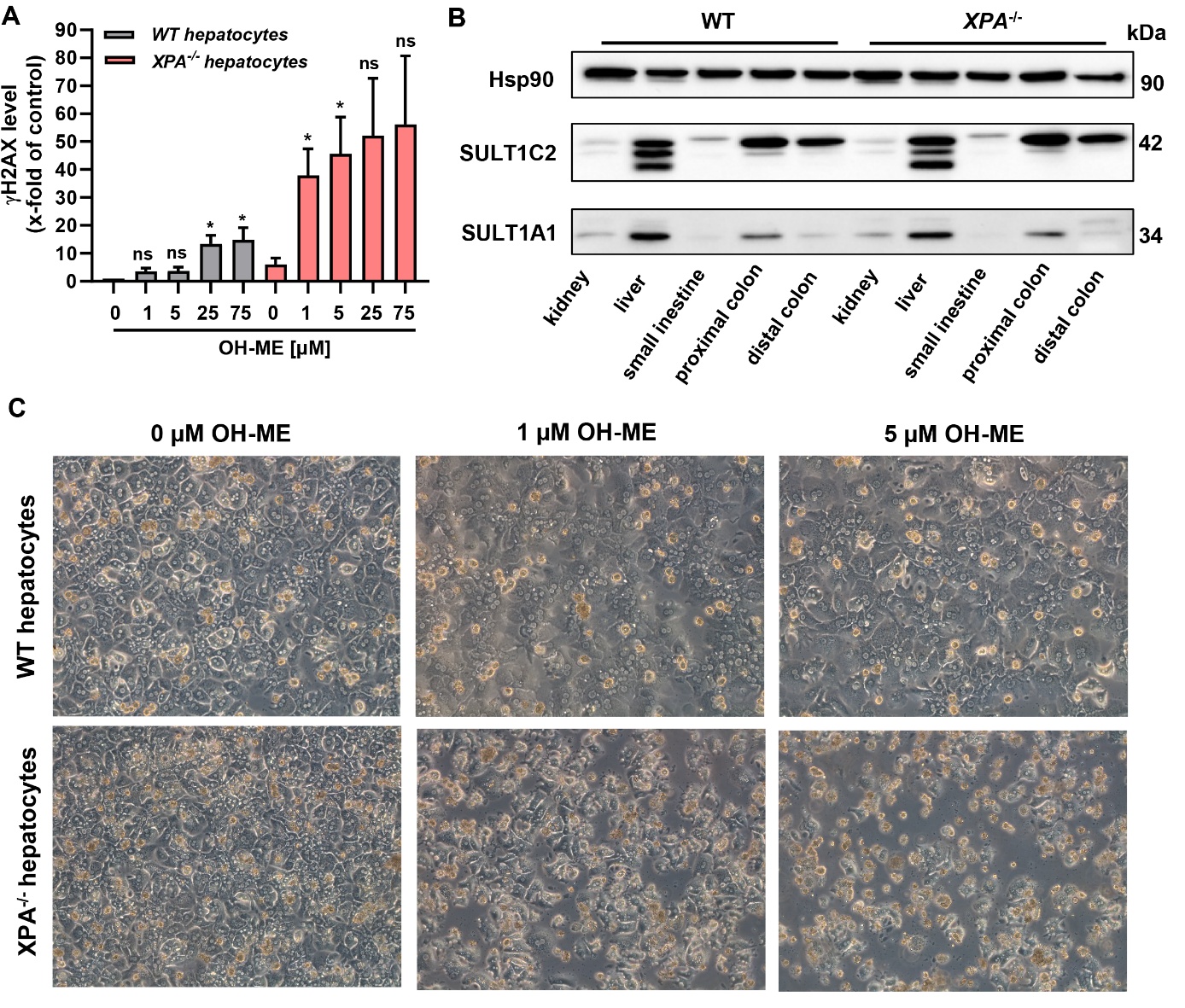
**

**Figure S4: Impact of XPA knockout on OH-ME triggered toxicity and tissue-specific SULT expression. A** OH-ME triggered DNA damage response in primary murine hepatocytes (PMH) proficient or deficient for XPA. PMH were exposed to increasing concentrations of OH-ME (0-75 µM) for 24 h and subjected to western blot analysis of γH2AX. Hsp90 served as loading control. Densitometric evaluation of western blots (n=3). **B** SULT expression in various tissues of WT and XPA^-/-^ mice. SULT1A1 and SULT1C2 expression were analyzed in tissue homogenates by SDS-PAGE and western blot detection. Hsp90 served as loading control. A representative Western blot is shown. **C** Representative light microscopy images of WT and XPA^-/-^ hepatocytes treated with up to 5 µM OH-ME for 24 h (100x magnification). Data presented as mean + SEM. Statistical analysis was performed using unpaired, two-sided t-test versus solvent control (ns p > 0.05, *p < 0.05).

**Figure S5**

**
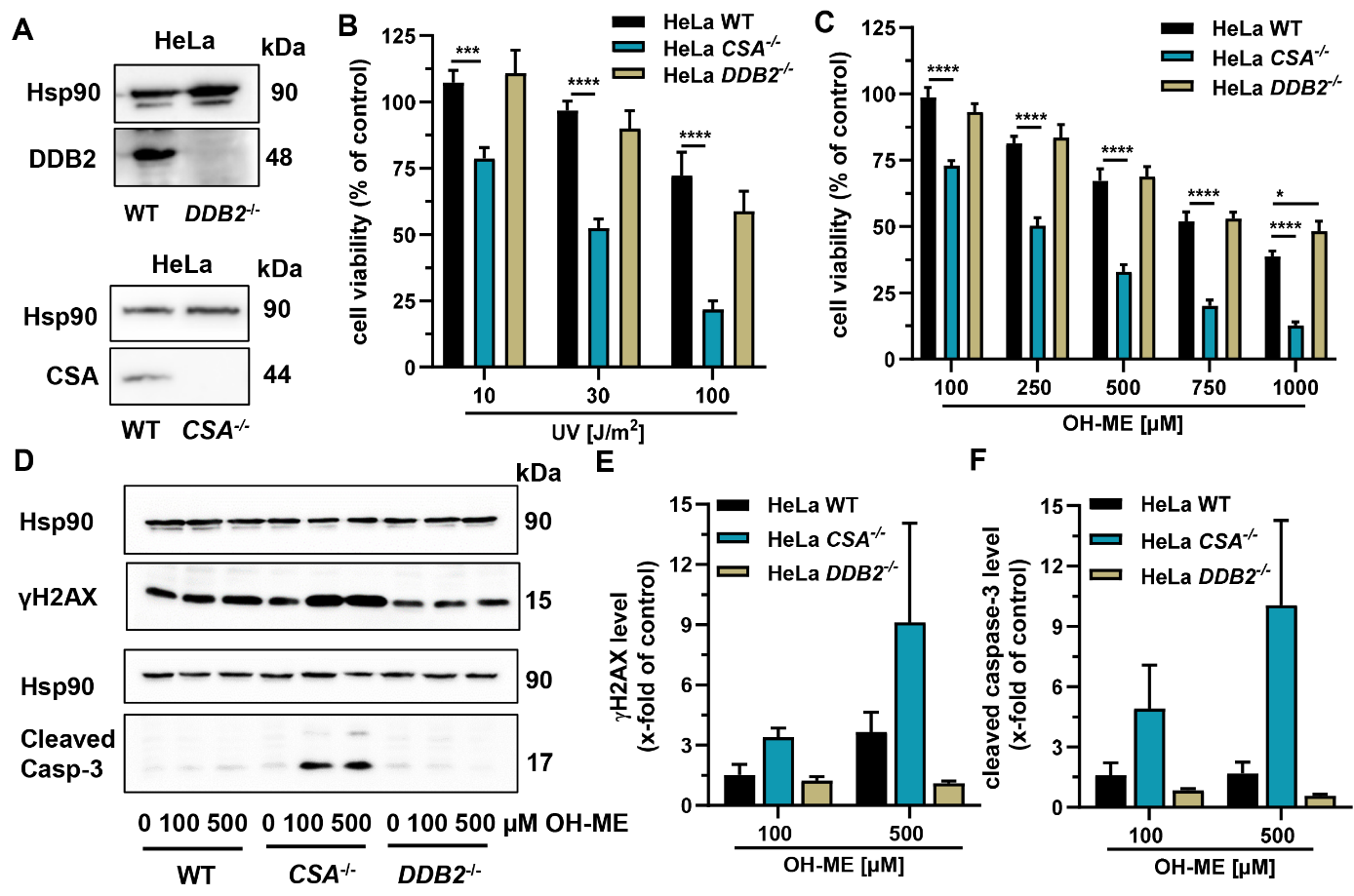
**

**Figure S5: Impact of DDB2 and CSA on cytotoxicity and DNA damage response upon OH-ME treatment. A** Re-validation of DDB2 and CSA knockout in Hela cells analyzed via SDS-PAGE and western blot detection. Hsp90 was detected as loading control. **B** Viability of HeLa WT, CSA^-/-^ and DDB2^-/-^ after UV-irradiation (10 – 100 J/m^2^). Viability was measured after 48 h by resazurin reduction assay (n=4). **C** Viability of Hela WT, CSA^-/-^ and DDB2^-/-^ cells after OH-ME treatment. Cells were incubated with increasing concentrations of OH-ME (0-1000 µM) for 48 h and viability was determined using resazurin reduction assay (n=4). **D** - **F** Analysis of the DNA damage response and apoptosis markers triggered by OH-ME in Hela WT, CSA^-/-^ and DDB2^-/-^. Cells were exposed to OH-ME (0-500 µM) for 48 h and then subjected to SDS-Page and western blot analysis of γH2AX and cleaved caspase-3. Hsp90 was detected as loading control. A representative western blot is shown in (**D**). Densitometric evaluation of γH2AX (**E**) and cleaved caspase-3 (**F**) (n=3). Data depicted as mean + SEM. Statistical analysis was performed using unpaired, two-sided t-test as indicated (*p < 0.05, ***p < 0.001, ****p < 0.0001)**.**

**Figure S6**

**
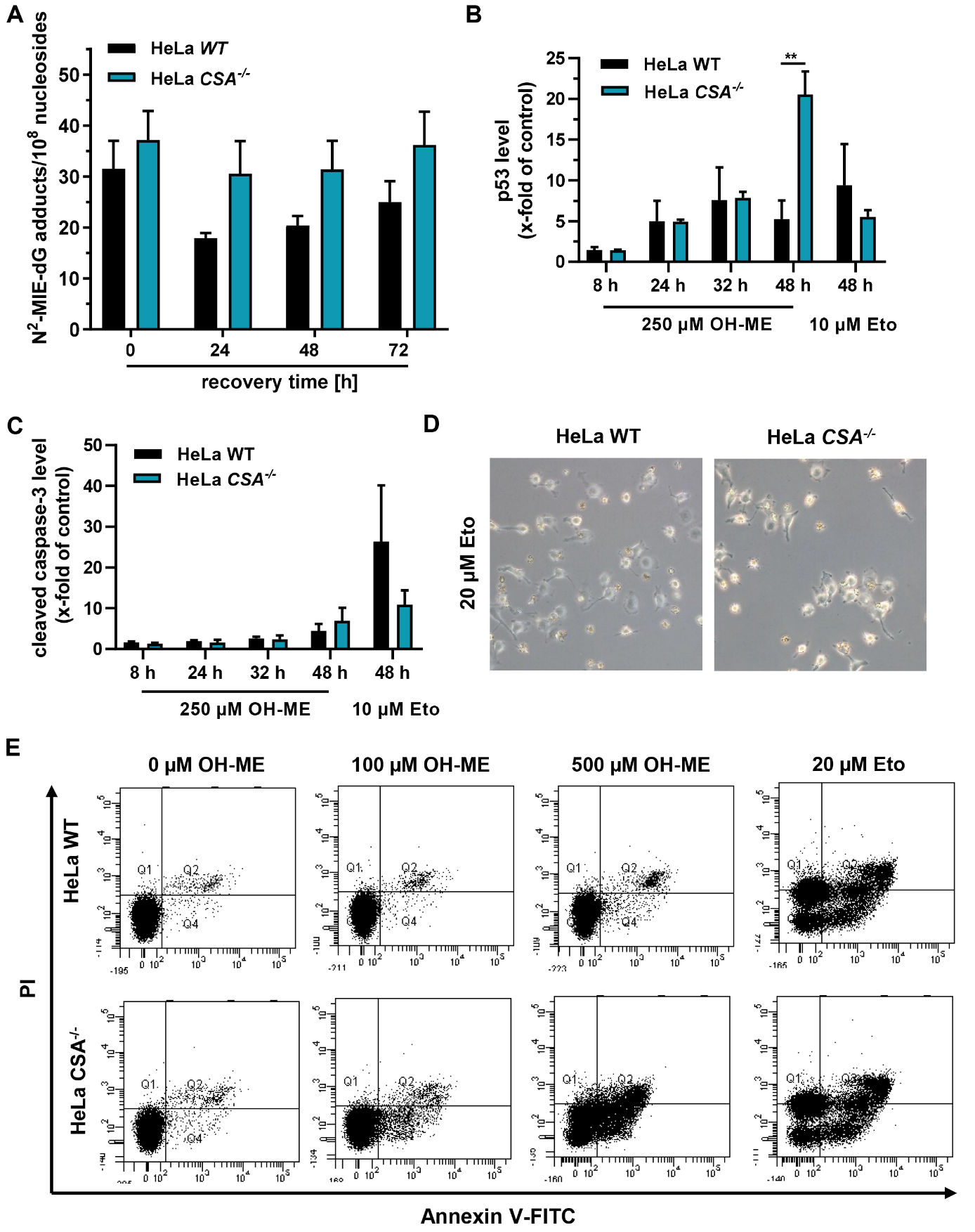
Figure S6: Influence of CSA-dependent TC-NER on DNA adduct formation and cytotoxicity triggered by OH-ME. A** Formation and persistence of N^2^-MIE-dG adducts in HeLa WT and CSA^-/-^ cells. The cells were treated with 100 µM OH-ME for 8 h. After medium exchange, cells were further cultivated for up to 72 h in fresh medium with reduced FCS content. Genomic DNA was isolated, digested to nucleosides and adducts levels were measured using stable isotope dilution analysis and UHPLC-mass spectrometry (n=4). **B** and **C** Time-dependent western blot analysis of cell death markers (cleaved caspase-3, p53) in HeLa WT and CSA^-/-^ incubated with 250 µM OH-ME for up to 48 h. 10 µM Eto was included as positive control. Densitometric evaluation of p53 (**B**) and cleaved-caspase-3 (**C**) levels (n=4). All data given as mean + SEM (**p < 0.01). **D** Representative light microscopy images of HeLa WT and CSA^-/-^ cells treated with 20 µM etoposide (Eto) or control for 48 h (100x magnification). **E** Cell death induction in HeLa WT and CSA^-/-^ upon OH-ME treatment. Cells were exposed to OH-ME (0 - 500 µM) or Eto (10 µM) for 48 h followed by Annexin V-FITC/PI staining and flow cytometry (n=4). Representative dot plots and gating are shown.

**Figure S7**

**
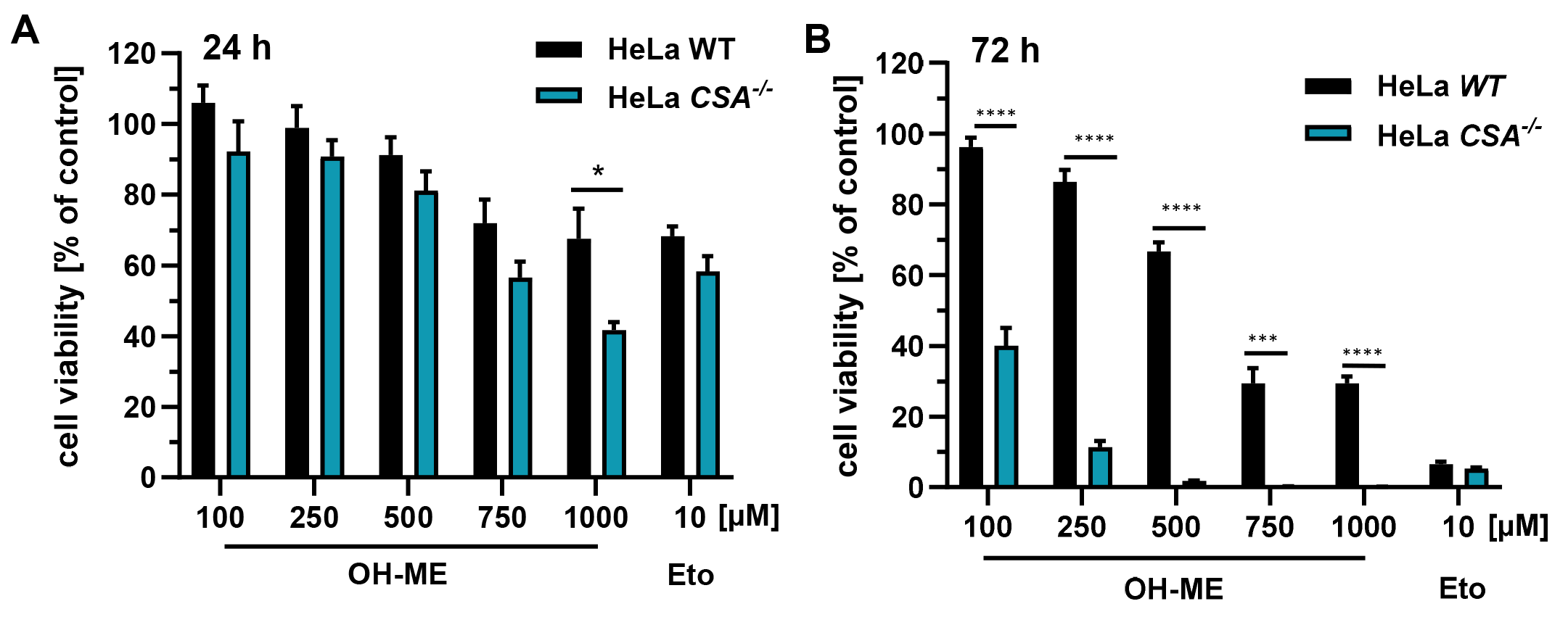
**

**Figure S7: Time-dependent cytotoxicity in HeLa WT and CSA^-/-^ cells upon OH-ME treatment.** **A and B** Viability of HeLa WT and HeLa CSA^-/-^ cells after treatment with increasing concentrations of OH-ME (0-1000 µM) for 24 h (**A**) or 72 h (**B**). Viability was assessed using resazurin reduction assay. All data are given as mean + SEM (n=4). Statistical analysis was performed using unpaired, two-sided t-test as indicated (*p < 0.05, ***p < 0.001, ***p < 0.0001)

**
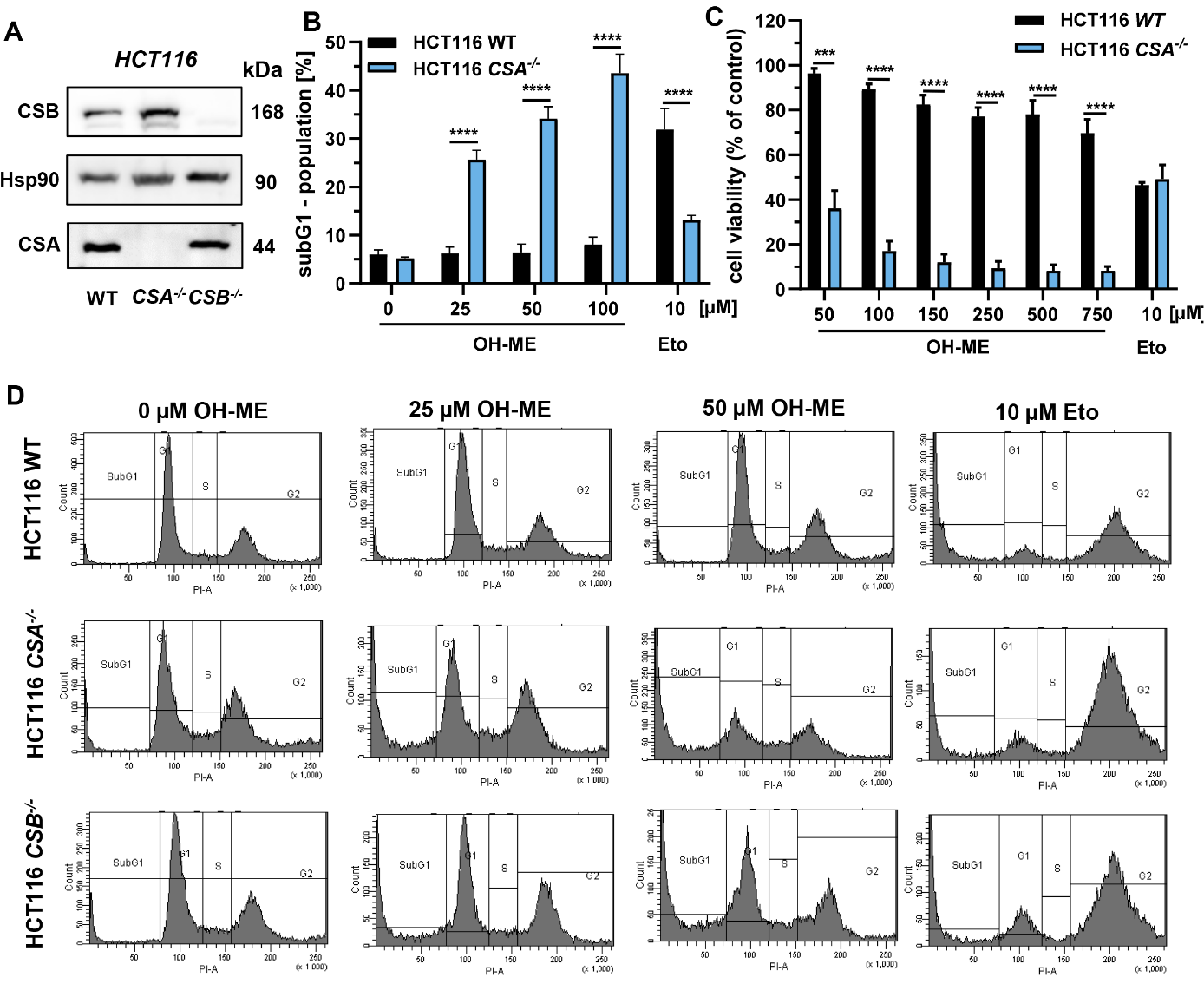
Figure S8**

**Figure S8: Impact of CSA and CSB knockout in HCT116 cells treated with OH-ME** **A** Re-validation of HCT116 WT, CSA^-/-^ and CSB^-/-^ cells using SDS-PAGE and western blot detection of CSA, CSB and XPA. Hsp90 served as loading control. **B** Analysis of subG1 population in HCT116 WT and CSA^-/-^ cells challenged with OH-ME (0-100 µM) for 48 h. SubG1 population indicative of cell death was assessed by PI staining and flow cytometry (n=4). **C** Viability of HCT116 WT and CSA^-/-^ cells upon treatment with increasing concentrations of OH-ME (0 - 1000 µM) for 48 h. Cell viability was determined by the resazurin reduction assay (n=4). **D** Representative histograms and gating of subG1 measurements in HCT116 WT, CSA^-/-^ and CSB^-/-^ cells treated for 48 h with OH-ME or etoposide (Eto). All data given as mean + SEM. Statistical analysis was performed using unpaired, two-sided t-test as indicated (****p < 0.0001).

**Figure S9**


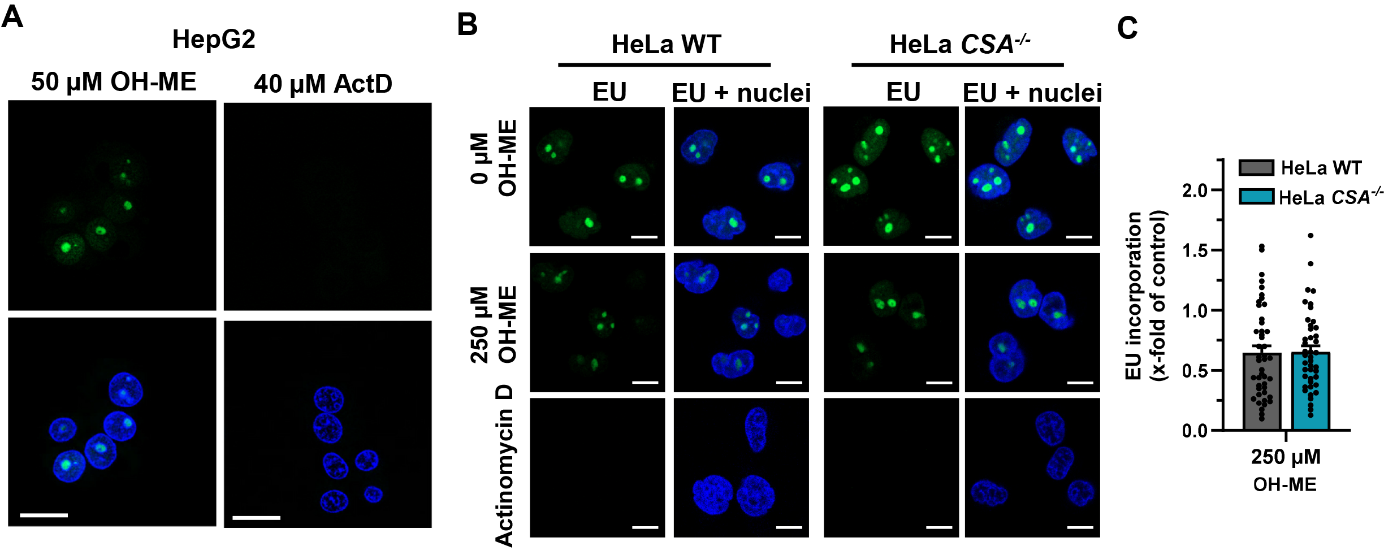


**Figure S9: Assessment of de novo transcription in HepG2 and HeLa cells upon OH-ME treatment. A** and **B** Assessment of de novo RNA synthesis upon OH-ME treatment for 24 h in HepG2 (**A**) and HeLa WT versus HeLa CSA^-/-^ cells (**B**). EU incorporation was analyzed by click chemistry with FAM-Azide (green), while nuclei were visualized by DAPI. Images were acquired by confocal microscopy and processed by Zen software. Representative images (scale bar: 10 µm) are shown. **C** Quantitative evaluation in HeLa WT and CSA^-/-^ cells treated with 250 µM OH-ME (n=4).

**Figure S10**

**Figure S10: Impact of OH-ME on RPB1 and phospho-RPB1 levels in HepG2 cells. A** Western blot detection of phospho-Ser2/Ser5 RPB1 (pRPB1) in HepG2 cells upon OH-ME treatment for 24 h in the absence or presence of the proteasome inhibitor MG132 (10 µM, 6 h before cell harvest). In addition, HepG2 cells were exposed to UV-C (20 J/m2) followed by 2 h incubation with or without MG132. Densitometric evaluation of pRPB1 levels are shown (n=3). **B - D** Subcellular localization of RPB1 and pRPB1 in HepG2 cells 24 h after OH-ME treatment with or without proteasome inhibition by MG132 (10 µM, 6 h before cell harvest). Cells were stained for RPB1 (red) and pRPB1 (green), while the nuclei were visualized by DAPI (blue). Images were acquired by confocal microscopy and processed by Zen software. Representative images (**B**, scale bar: 10 µm) and the quantitative evaluation of nuclear pRPB1 (**C**) and RPB1 (**D**) levels are shown (n=4). All data are given as mean + SEM. Statistical analysis was performed using unpaired, two-sided t-test. *p < 0.05, **p < 0.01, ***p<0.001, ****p < 0.0001.

**
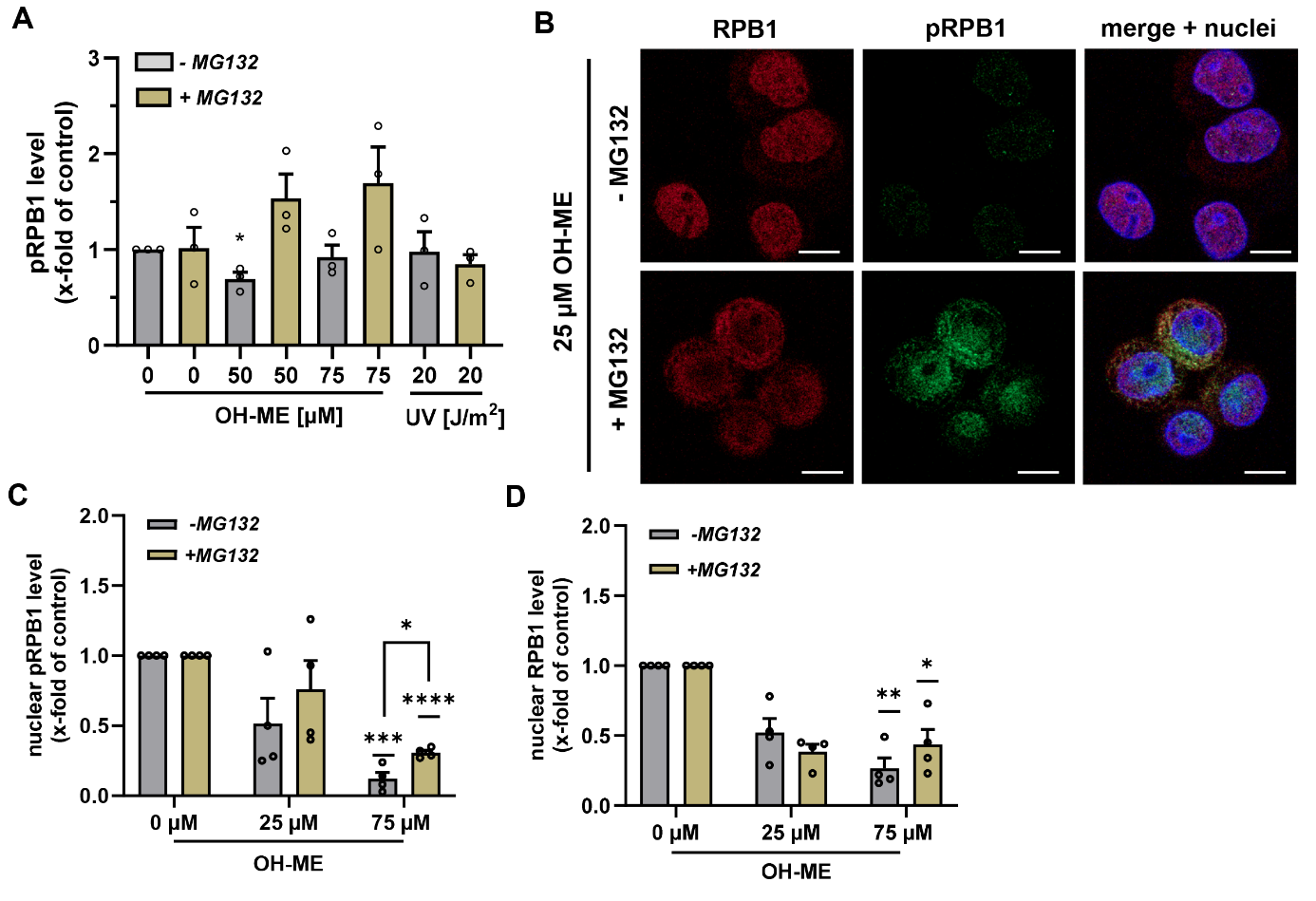
**

**Figure S11**

**
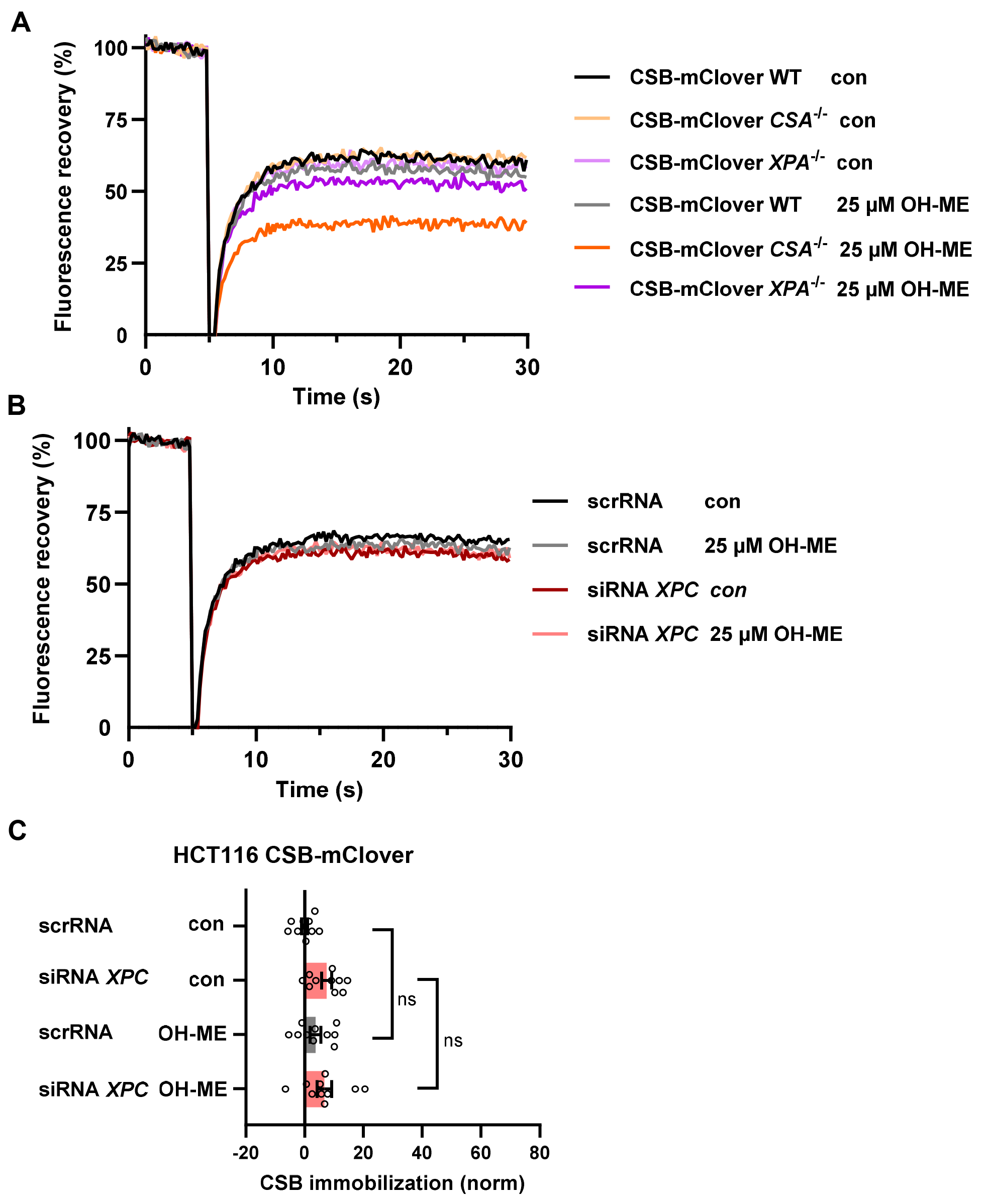
Figure S11: Impact of NER factors on CSB immobilization upon OH-ME treatment. A** Fluorescence recovery curves of HCT116 CSB-mClover WT, CSA^-/-^ or XPA^-/-^ cells, mock- or treated with 25 μM OH-ME for 16 h prior to imaging. Fluorescence recovery was measured during 30 s after bleaching and normalized to average pre-bleach intensities (100 %). **B** Fluorescence recovery curves of HCT116 CSB-mClover WT cells transfected with scrambled RNA (scrRNA) as control or with XPC siRNA for 48 h, followed by mock treatment or exposure to 25 μM OH-ME for 16 h prior to FRAP measurements as mentioned in A. **C** Percentage of CSB immobile fraction in HCT116 CSB-mClover WT cells, derived from FRAP analyses shown in panel (B). Data represent the mean ± SEM of 30 cells from at least three independent experiments. Statistical analysis performed by two-tailed, nested t-test. Ns, not significant, p>0.05.

**Fig. S12**

**Figure S12: DNA damage response in HCT116 CSB-mClover WT, CSA^-/-^ or XPA^-/-^ cells after OH-ME treatment versus UV-C irradiation.** Cells were treated with solvent control (Con) or 100 μM OH-ME for up to 24 h or irradiated with UV-C (10 J/m^2^) followed by 24 h incubation. **A** Western blot detection of p53 and γH2AX. Hsp90 was visualized as loading control. A representative western blot is shown (n=4). **B** and **C** Densitometric evaluation of p53 (**B**) and γH2AX (**C**) levels. Data are given as mean + SEM. Statistical analysis was performed using unpaired, two-sided t-test. *p < 0.05, **p < 0.01.

**
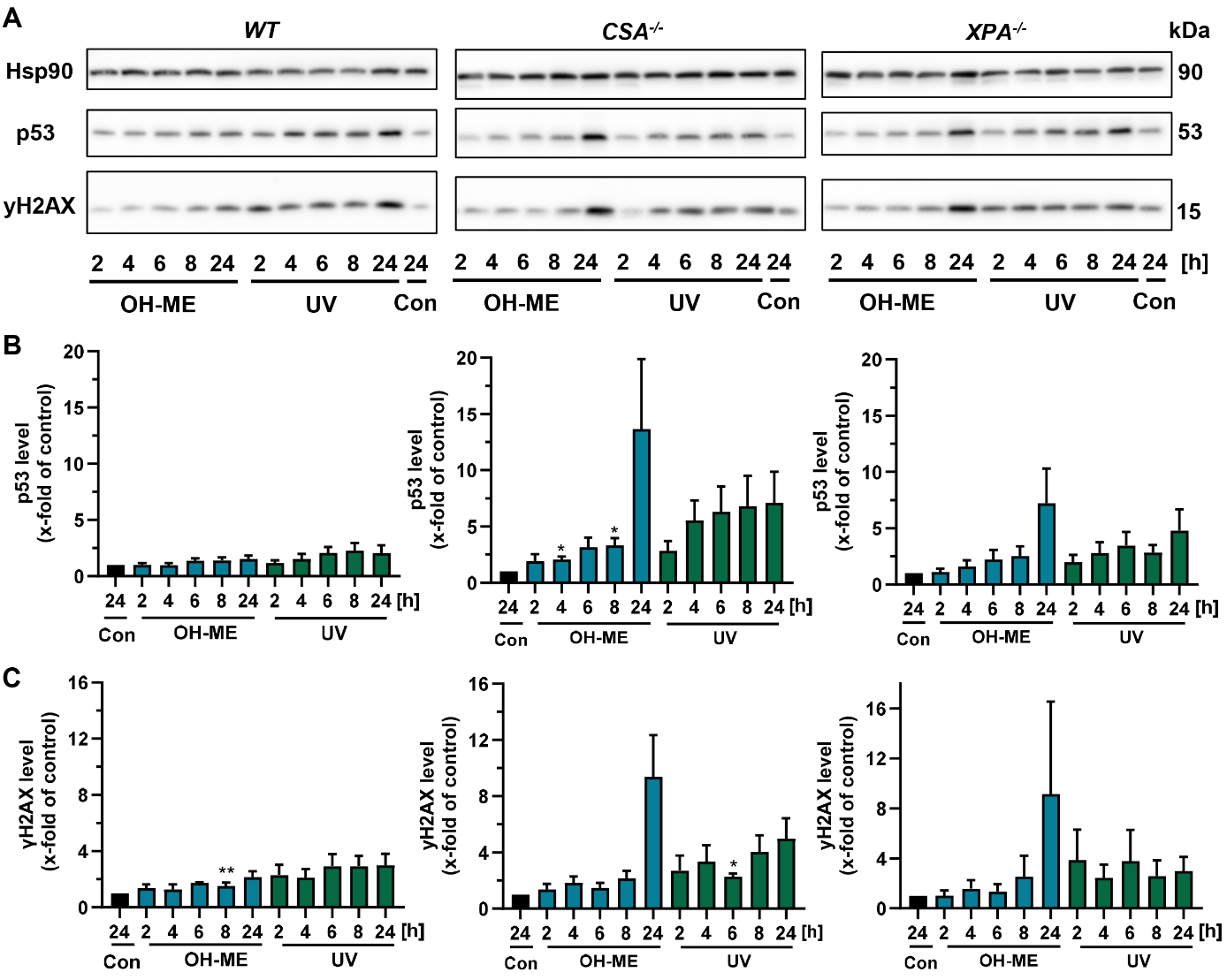
**

**Figure S13**

**
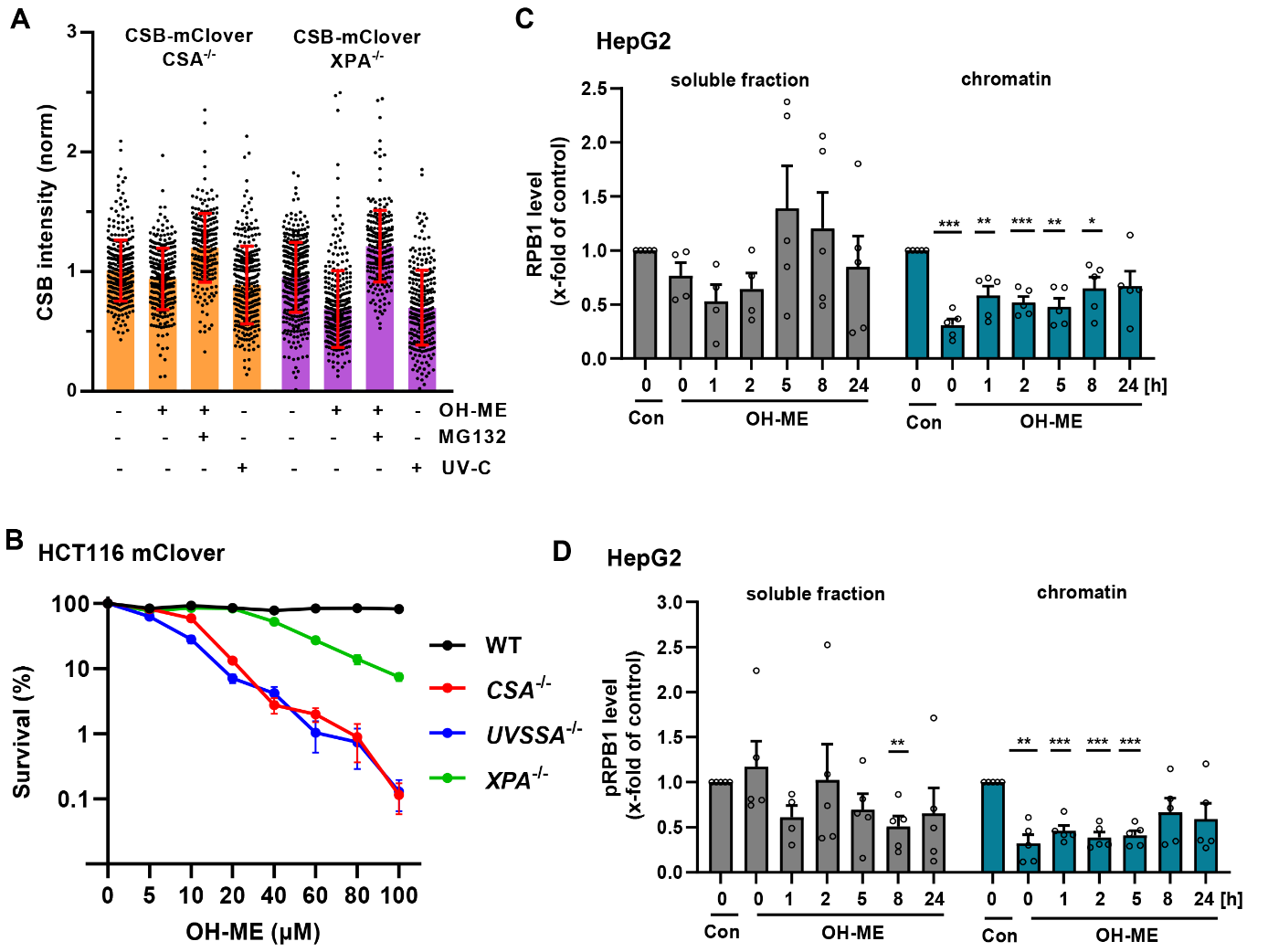
**

**Figure S13: OH-ME triggered transcription stress in HCT116 CSB-mClover and transcription recovery in HepG2 cells. A** Endogenous CSB-mClover fluorescence in HCT116 CSB-mClover CSA^-/-^ or XPA^-/-^. Cells were mock-treated or exposed to 25 μM OH-ME with or without 2 μM MG132 for 16 h, or UV-C irradiated (4 J/m^2^) followed by 16 h recovery prior to fixation and DAPI staining. Fluorescence was background-corrected using non-fluorescent HCT116 WT cells and normalized to non-irradiated condition. Bars represent mean fluorescence intensities ± SD pooled from three independent experiments. **B** Colony survival assay in HCT116 CSB-mClover WT, CSA^-/-^, UVSSA^-/-^ and XPA^-/-^ cells challenged with increasing concentrations of OH-ME. Data are shown as mean + SD (n=3). **C** and **D** Analysis of the RNA polymerase II subunit RPB1 and phospho-RPB1 (Ser-2/5) in HepG2. Cells were treated with 75 µM OH-ME for 16 h and then harvested after 0-24 h of recovery time followed by cell fractionation. The cytosolic marker Hsp90 and the chromatin marker Histone H3 served as loading controls. Densitometric evaluation of RPB1 (**C**) and pRPB1 (**D**) levels are shown (n=5). Data are given as mean + SEM. Statistical analysis was performed using unpaired, two-sided t-test versus control (*p < 0.05, **p < 0.01, ***p < 0.001).

**Fig. S14**

**
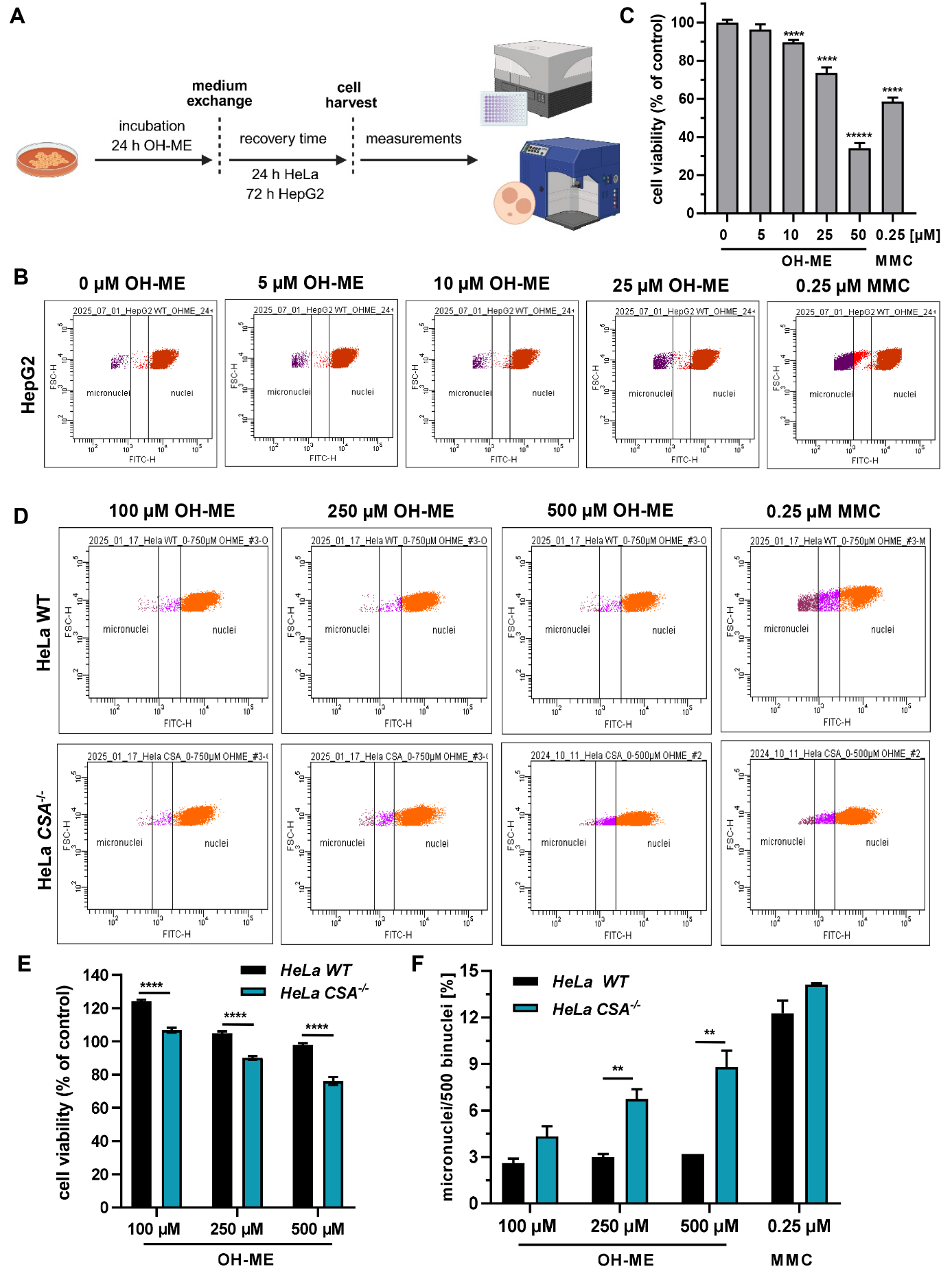
Figure S14: OH-ME triggered micronucleus formation in HepG2 and HeLa cells. A** Experimental setup of flow cytometry-based micronucleus assay. Created in BioRender. Fahrer, J. (2026) <https://BioRender.com/52ruwfv> **B** Viability of HepG2 cells after OH-ME exposure as depicted in (**A)**. Mitomycin C (MMC) was used as positive control. Data are given as mean + SEM (n=3). **C** Representative dot plots and gating of flow cytometry-based micronucleus assay performed in HepG2 cells as shown in (**A)**. **D** Representative dot plots and gating of flow cytometry-based micronucleus assay performed in HeLa WT and CSA^-/-^ cells as indicated in (**A)**. **E** Viability of HeLa WT and CSA^-/-^ cells after OH-ME exposure as depicted in (**A**). **F** Cytokinesis block micronucleus assay in HeLa WT and CSA^-/-^ cells after exposure to OH-ME (100 – 500 µM) or 0.25 µM MMC as positive control. Data shown as mean + SEM (n=3). Statistical analysis was performed using two tailed, unpaired t-test as indicated (**p < 0.01, ****p < 0.0001).

**Supplementary Tables**

**Table S1: Compound specific mass spectrometric parameters for** **dG, [*^15^N_5_*]-dG, *N^2^*-MIE-dG, *N^6^*-MIE-dA, [*^15^N_5_*]- *N^2^*-MIE-dG and [*^15^N_5_*]- *N^6^*-MIE-dA.** Q1, quadrupole 1 (*m/z*); Q3, quadrupole 3 (*m/z*); DP, declustering potential (V); FP, focusing potential (V); EP, entrance potential (V); CEP, collision cell entrance potential (V); CE, collision energy (V); CXP, cell exit potential (V); *Transitions used as quantifier.

|  | **Q1** | **Q3** | **DP** | **FP** | **EP** | **CEP** | **CE** | **CXP** |
| --- | --- | --- | --- | --- | --- | --- | --- | --- |
| dG1 | 268.175 | 151.9* | 6 | 350 | 4.5 | 12 | 15 | 18 |
| dG2 | 268.175 | 134.9 | 6 | 350 | 4.5 | 12 | 47 | 20 |
| [*^15^N_5_*]-dG1 | 273.083 | 157.3* | 26 | 370 | 4 | 20 | 17 | 4 |
| [*^15^N_5_*]-dG2 | 273.083 | 138.9 | 26 | 370 | 4 | 20 | 49 | 4 |
| *N*^2^-MIE-dG1 | 444.166 | 328.1* | 46 |  | 10 |  | 17 | 34 |
| *N*^2^-MIE-dG2 | 444.166 | 177.1 | 46 |  | 10 |  | 33 | 12 |
| *N*^6^-MIE-dA1 | 428.170 | 177.1* | 71 |  | 10 |  | 35 | 14 |
| *N*^6^-MIE-dA2 | 428.170 | 312.1 | 71 |  | 10 |  | 21 | 22 |
| [*^15^N_5_*]-*N*^2^-dG1 | 449.078 | 333.1* | 61 |  | 10 |  | 15 | 24 |
| [*^15^N_5_*]-*N*^2^-dG2 | 449.078 | 177.1 | 61 |  | 10 |  | 41 | 12 |
| [*^15^N_5_*]-*N*^6^-dA1 | 433.094 | 177.1* | 56 |  | 10 |  | 33 | 12 |
| [*^15^N_5_*]-*N*^6^-dA2 | 433.094 | 317.1 | 56 |  | 10 |  | 21 | 18 |

**Table S2:** **Antibodies used in this study.**

| **Antibody** | **Catalog No.** | **Provider** |
| --- | --- | --- |
| Anti-p53 (DO1), mouse monoclonal | sc-126 | Santa Cruz Biotechnology,  Heidelberg, Germany |
| Anti-γH2AX, rabbit monoclonal | ab81299 | Abcam, Cambridge, UK |
| Anti-Hsp90α/β (F-8), mouse monoclonal | sc-13119 | Santa Cruz Biotechnology,  Heidelberg, Germany |
| Anti-XPA, mouse monoclonal | sc-28353 | Santa Cruz Biotechnology,  Heidelberg, Germany |
| Anti-pCHK1 (Ser345), rabbit monoclonal | #2348T | Cell Signaling Technology,  Danvers, Massachusetts, USA |
| Anti-pCHK2 (Thr68), rabbit monoclonal | #2197 | Cell Signaling Technology,  Danvers, Massachusetts, USA |
| Anti-Cleaved Caspase-3 (Asp175), rabbit monoclonal | #9664 | Cell Signaling Technology,  Danvers, Massachusetts, USA |
| Anti-CSA (ERCC8), rabbit monoclonal | ab137033 | Abcam, Cambridge, UK |
| Anti-CSB (ERCC6), rabbit monoclonal | ab316744 | Abcam, Cambridge, UK |
| Anti-Histon H3, rabbit polyclonal | GTX122148 | Genetex, Irvine, California, USA |
| Anti-DDB2 (XPE), rabbit monoclonal | ab181136 | Abcam, Cambridge, UK |
| Anti-RPB1, mouse monoclonal | #2629 | Cell Signaling Technology,  Danvers, Massachusetts, USA |
| Anti-phospho-RPB1 (Ser2/Ser5), rabbit monoclonal | #13546 | Cell Signaling Technology, Danvers, Massachusetts, USA |
| Anti-S9.6, mouse monoclonal | MABE1095 | Merck, Darmstadt, Germany |
| mouse IgGκ binding protein-HRP | sc-516102 | Santa Cruz Biotechnology,  Heidelberg, Germany |
| Goat-anti-Rabbit-HRP | #7074 | Cell Signaling Technology,  Danvers, Massachusetts, USA |
| F(ab’)2-Goat-anti-Mouse IgG (H-L) Alexa Fluor 488, cross-adsorbed | A-11017 | Life Technologies, Darmstadt, Germany |
| Goat anti-Rabbit IgG (H+L) Alexa Fluor 488, cross-adsorbed | A-11008 | Life Technologies, Darmstadt, Germany |
| F(ab')2-Goat anti-Mouse IgG (H+L) Alexa Fluor 647, cross-adsorbed | A-21237 | Life Technologies, Darmstadt, Germany |

**Table S3: Determination of EC_50_ values based on viability data in HeLa and HCT116 cells.** Concentrations were transformed into the log scale and plotted against viability. The data were then fitted by nonlinear regression with variable slope using GraphPad Prism 8. *n.d. not determinable, n.a. not analyzed.

|  | **EC_50_ (24 h)** | **EC_50_ (48 h)** | **EC_50_ (72 h)** |
| --- | --- | --- | --- |
| HeLa WT | n.d. | 868.7 µM | 610.7 µM |
| HeLa *CSA^-/-^* | 853.5 µM | 245.5 µM | 81.8 µM |
| HCT116 WT | n.d. | n.d. | n.a. |
| HCT116 *CSA^-/-^* | 210.9 µM | 24.6 µM | n.a. |
| HCT116 *CSB^-/-^* | 185.8 µM | 54.7 µM | n.a. |
