# Supplementary material for: Transcription-coupled nucleotide excision repair protects against genomic instability and cell death induced by the liver toxin methyleugenol
Source: Cell Death Dis. 2026 May 19;17(1):483. doi: 10.1038/s41419-026-08853-4 (PMC13186962; doi:10.1038/s41419-026-08853-4)

Fig. 2A and Fig. S2A-C

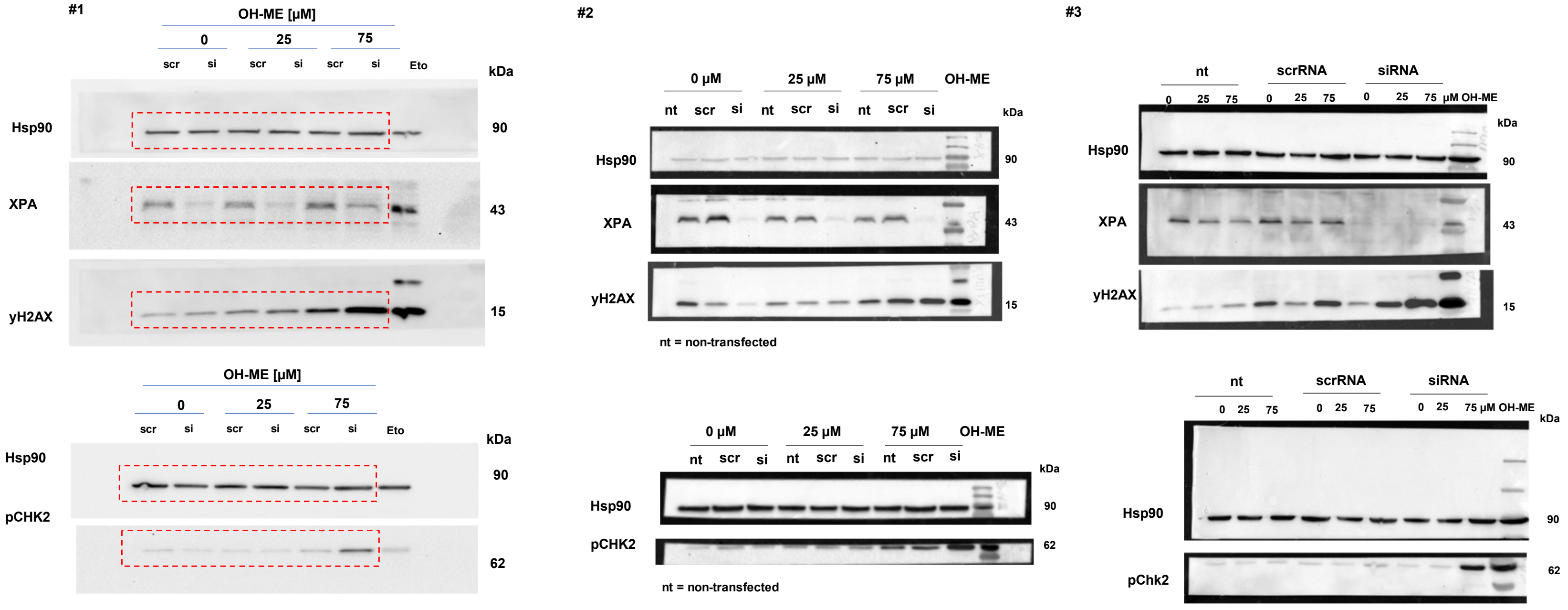

Fig. 2B and Fig. S2D-E

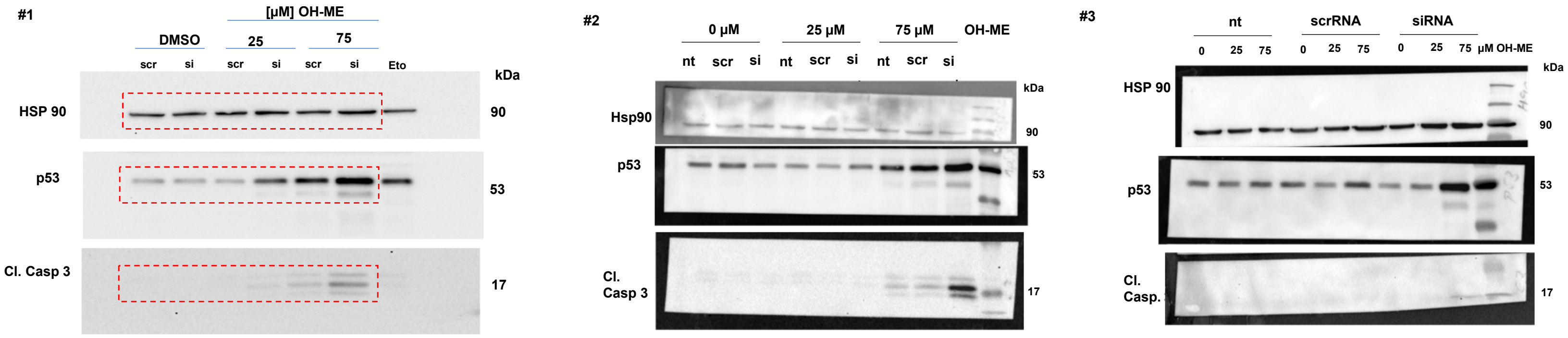

Fig. 2E and Fig. S3AC

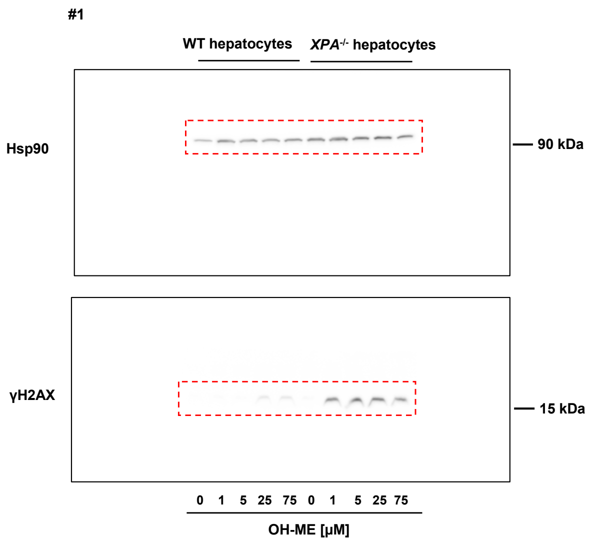

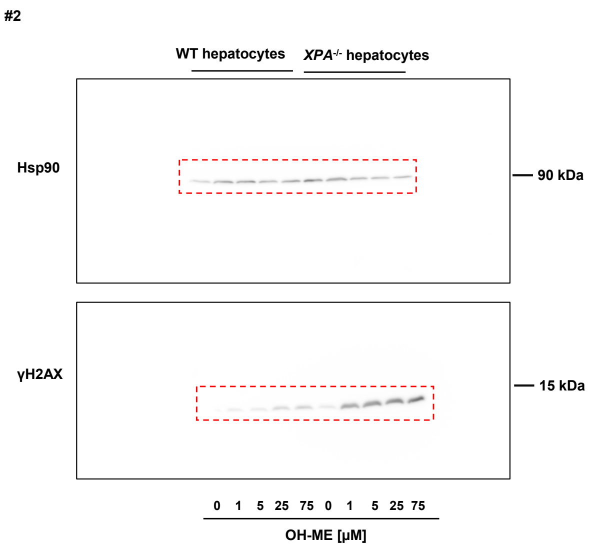

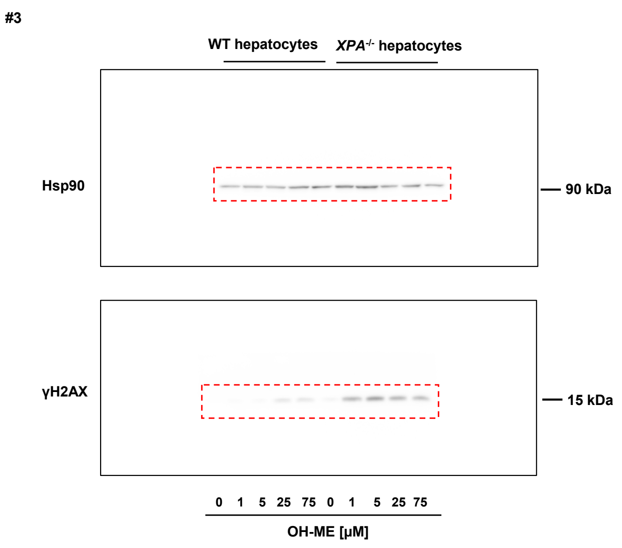

Fig. 3A and B & D

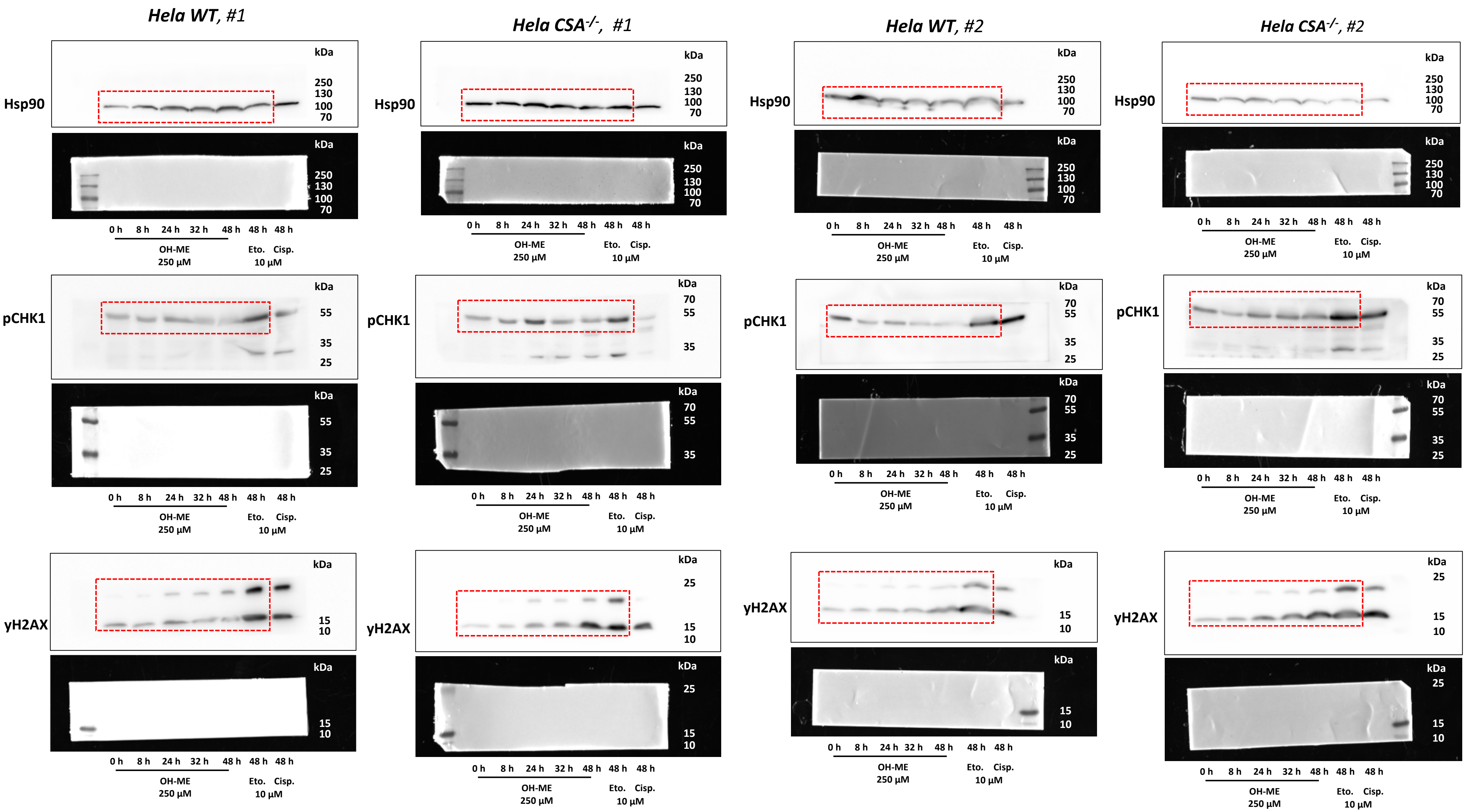
Hela WT, #1
Hela CSA-/-, #1
Hela WT, #2
Hela CSA-/-, #2
Hsp90
pCHK1
yH2AX
kDa
250
130
100
70
55
35
25
15
10
0 h
8 h
24 h
32 h
48 h
OH-ME
250 µM
Eto.
Cisp.
10 µM

Fig. 3A and B & D

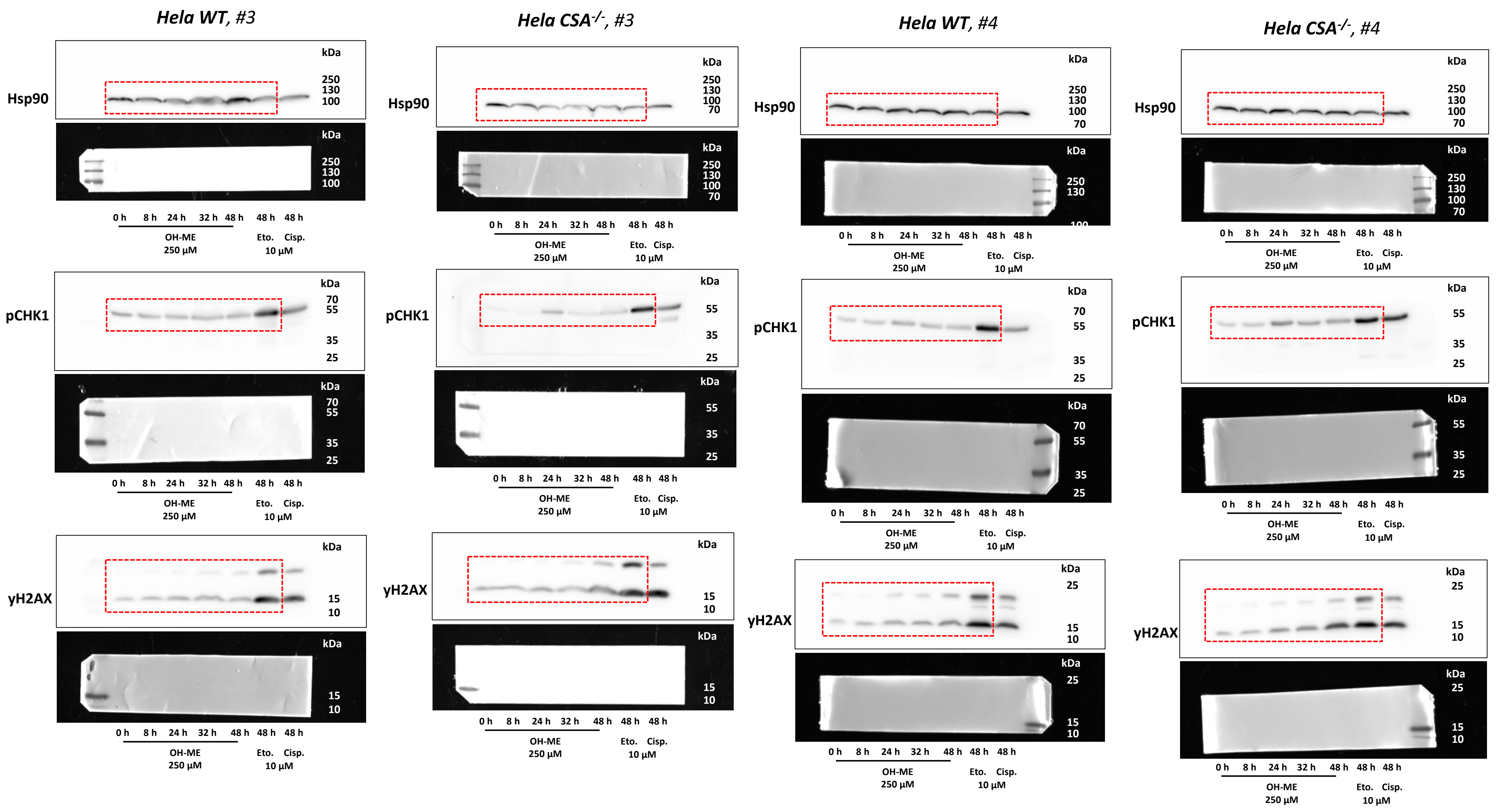
Hela WT, #3
Hela CSA-/-, #3
Hela WT, #4
Hela CSA-/-, #4
Hsp90
pCHK1
yH2AX
kDa
0 h
8 h
24 h
32 h
48 h
OH-ME
250 µM
Eto.
Cisp.
10 µM

Fig. 3A and C

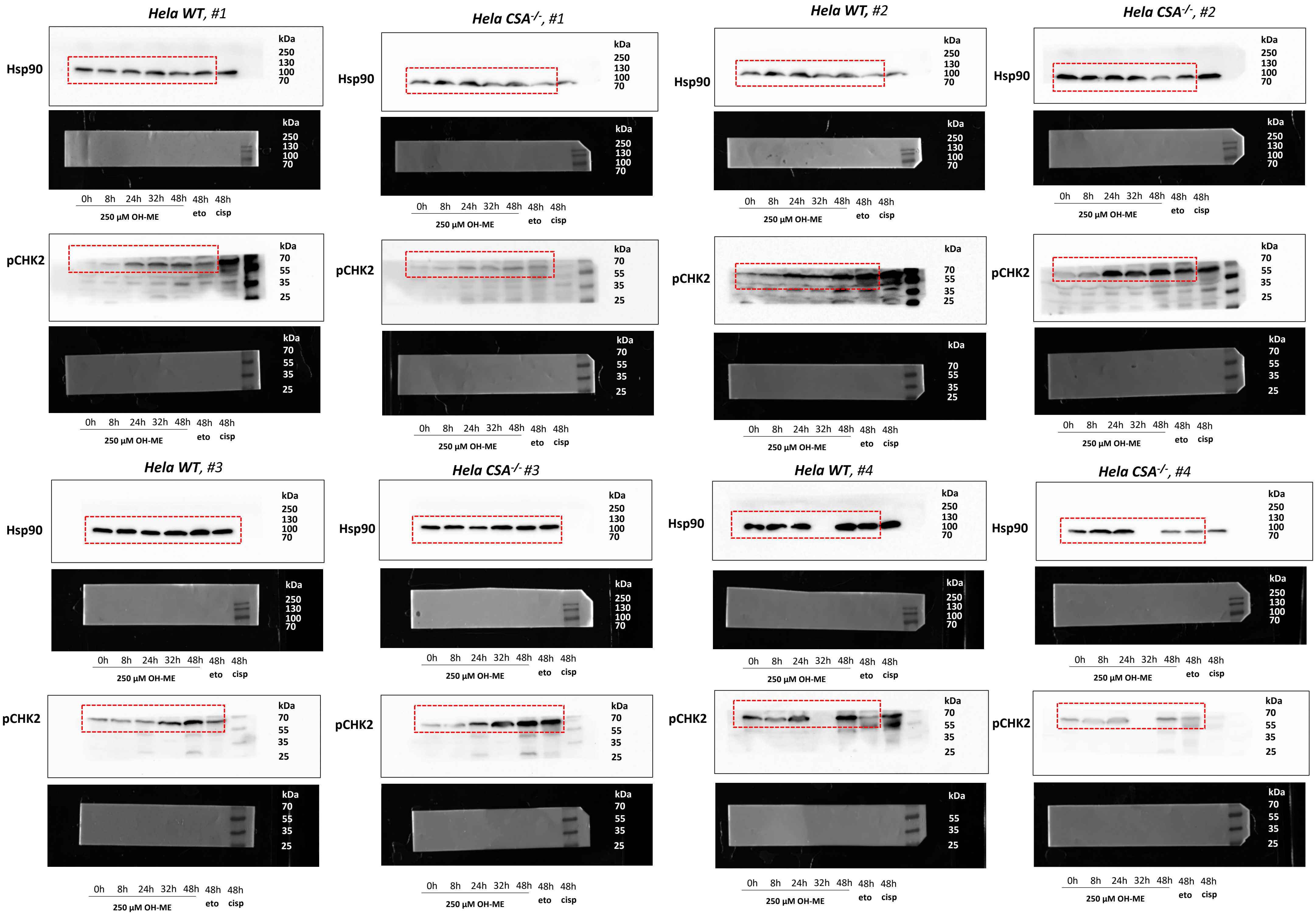
Hela WT, #1
Hela CSA-/-, #1
Hela WT, #2
Hela CSA-/-, #2
Hela WT, #3
Hela CSA-/- #3
Hela WT, #4
Hela CSA-/-, #4
Hsp90
pCHK2
kDa
250
130
100
70
55
35
25
0h 8h 24h 32h 48h 48h 48h
250 µM OH-ME
eto
cisp

Fig. 4A and Fig. S5C & D

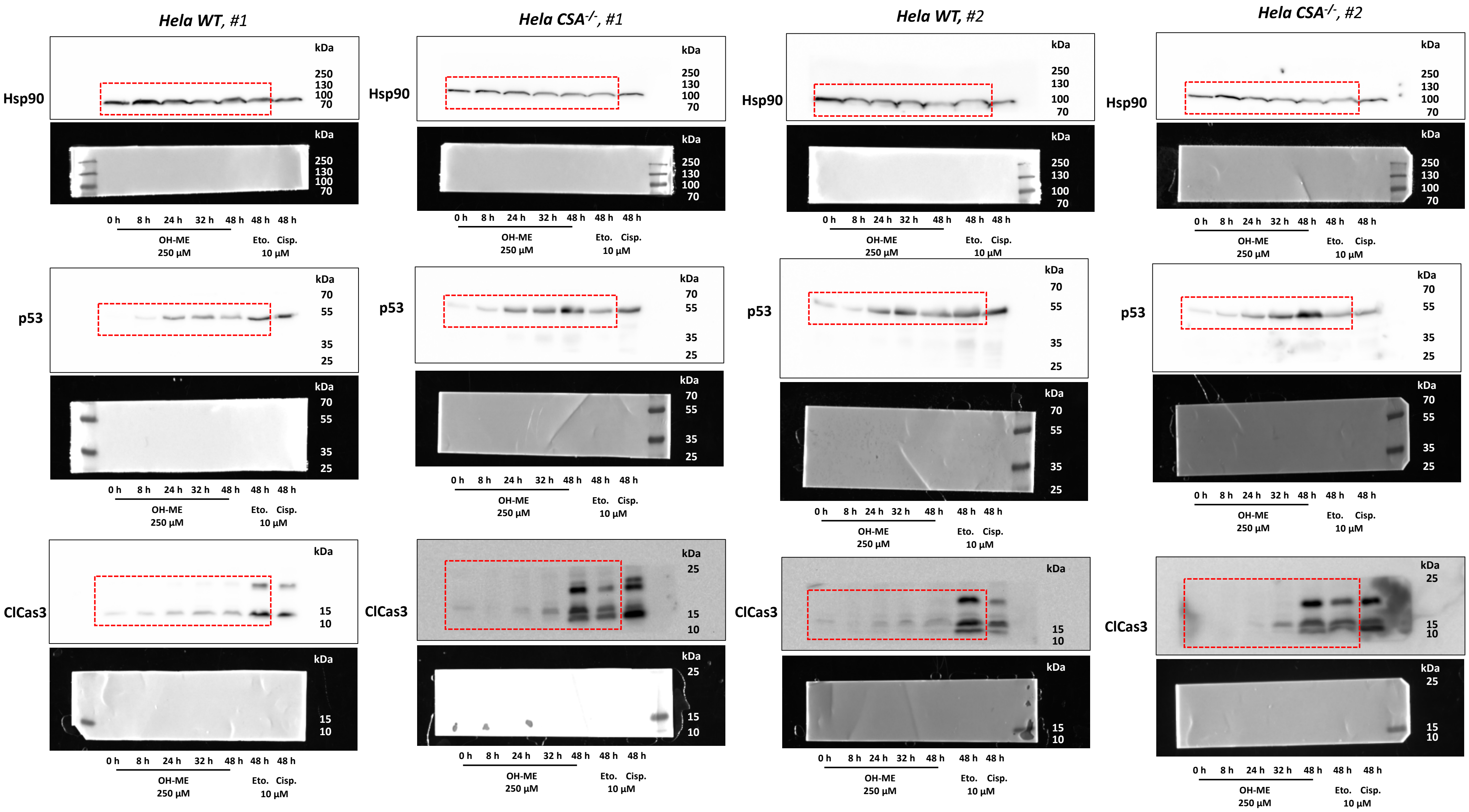

Fig. 4A and Fig. S5C & D

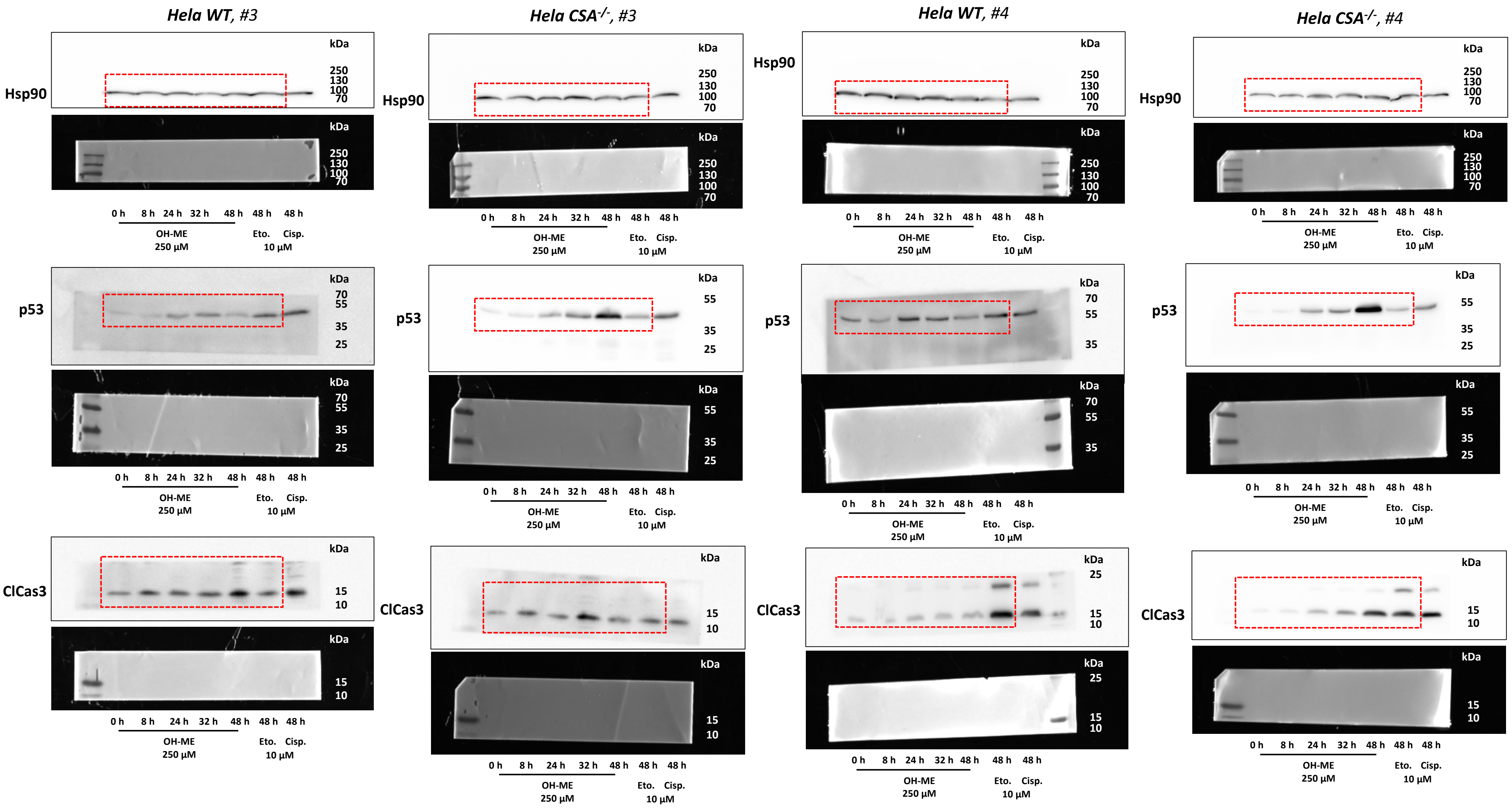

Fig. 5E and F and Fig. S10A

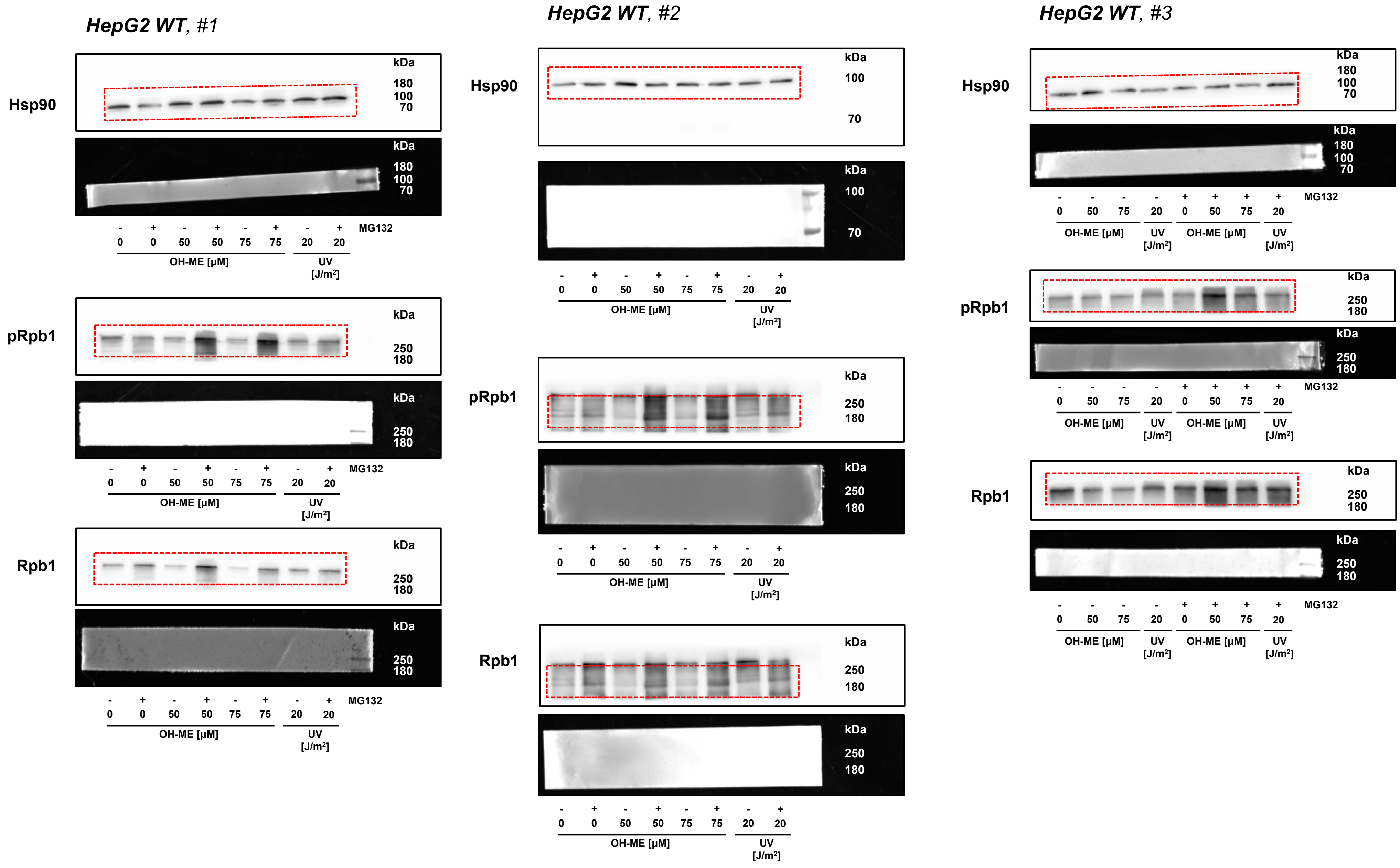

Fig. 6E and Fig. S13C and D

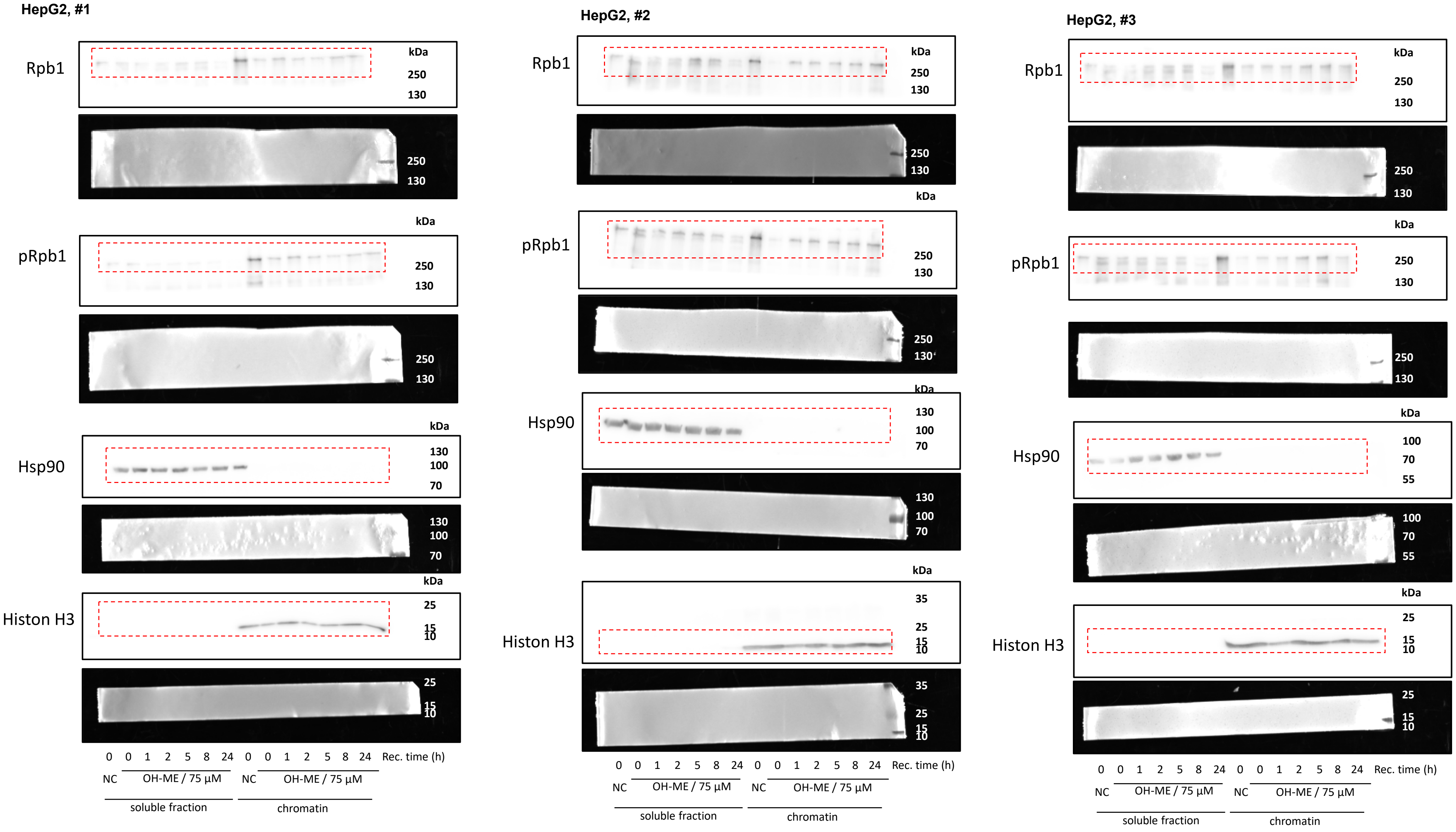

Fig. 6E and Fig. S13C and D (continued)

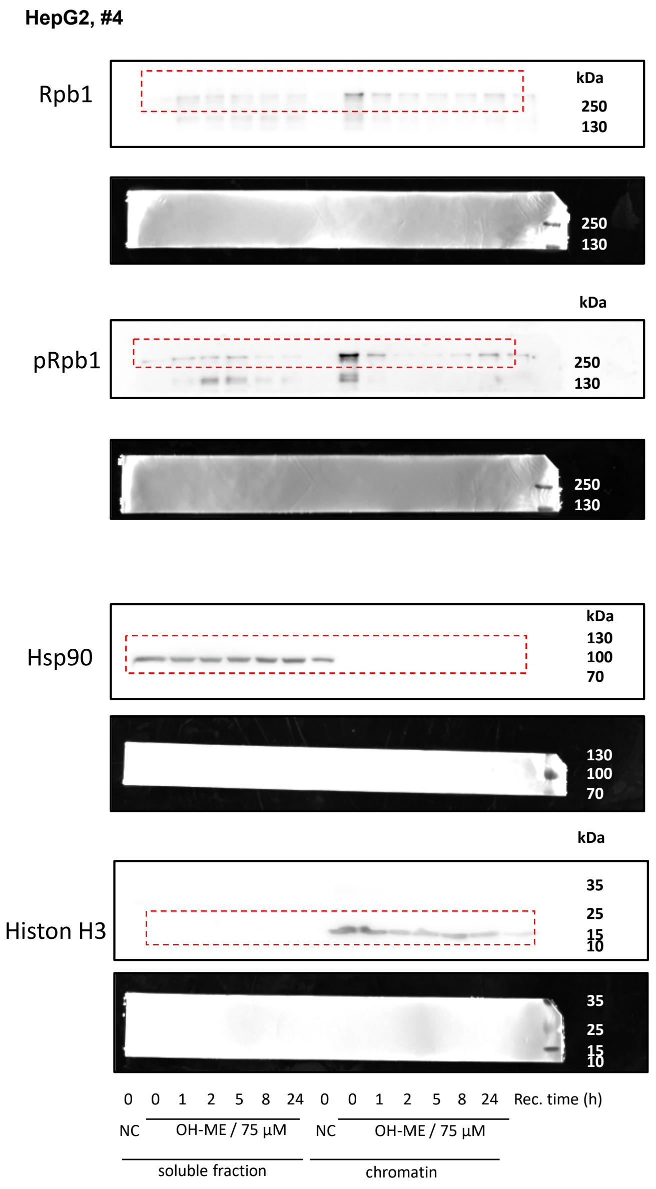

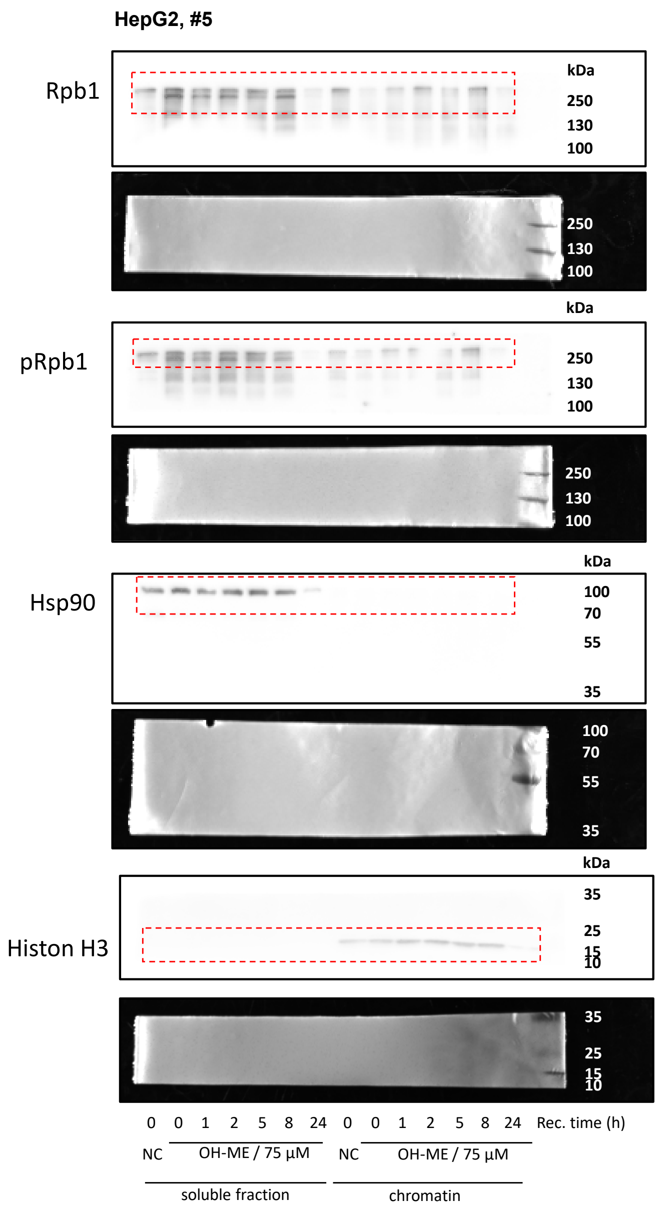

Fig. S3G

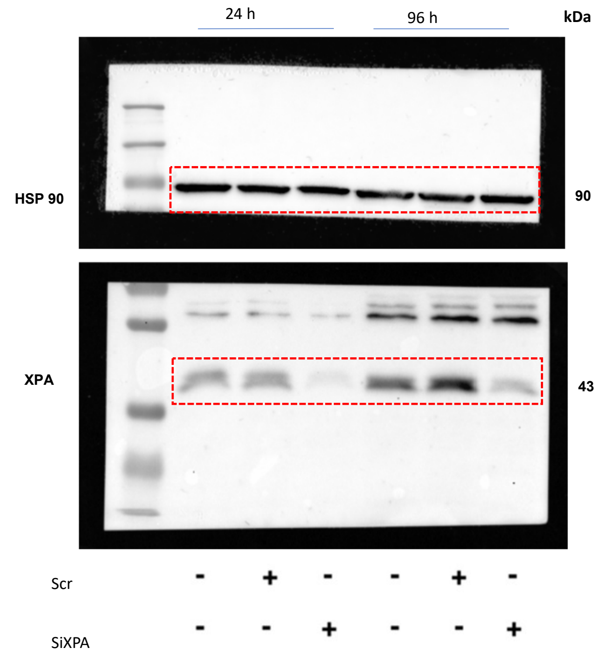

Fig. S4B

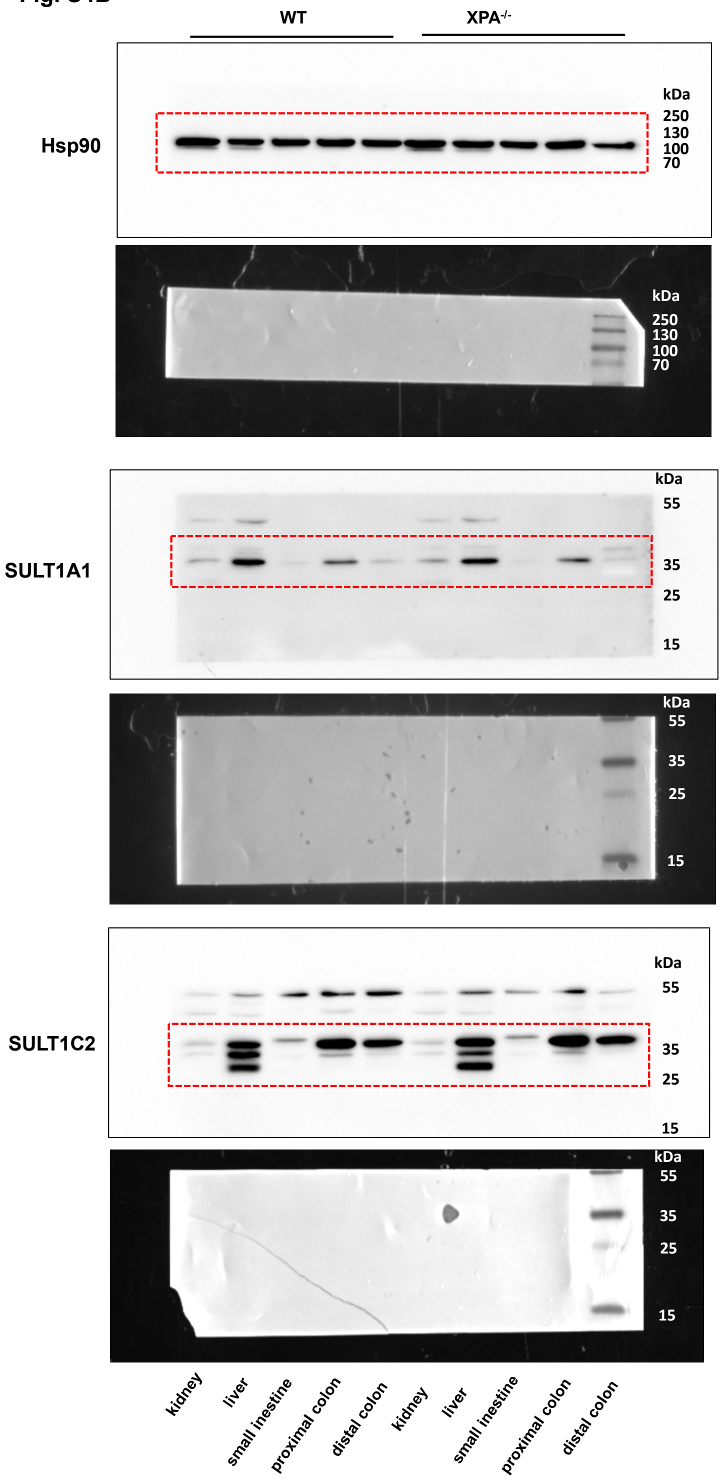

Fig. S5A

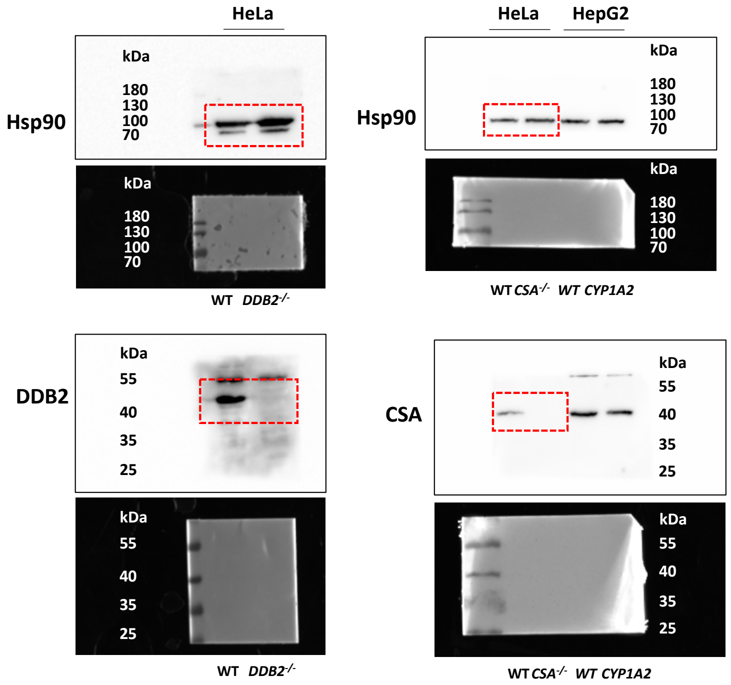

Fig. S5D &E

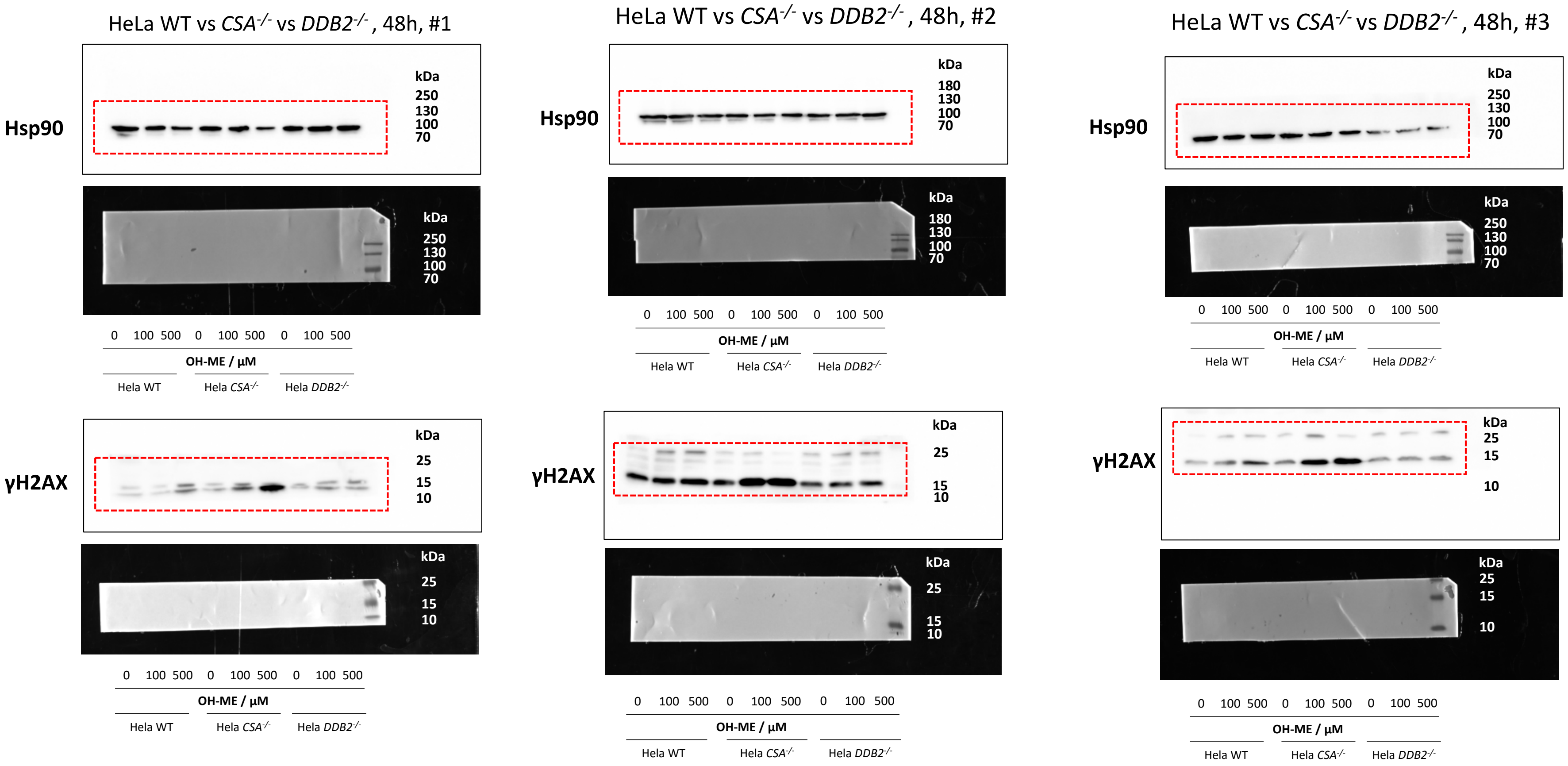

HeLa WT vs CSA-/- vs DDB2-/- , 48h, #1
HeLa WT vs CSA-/- vs DDB2-/- , 48h, #2
HeLa WT vs CSA-/- vs DDB2-/- , 48h, #3
Hsp90
γH2AX
kDa
250
180
130
100
70
25
15
10
0 100 500 0 100 500 0 100 500
OH-ME / µM
Hela WT
Hela CSA-/-
Hela DDB2-/-

Fig. S5D & F

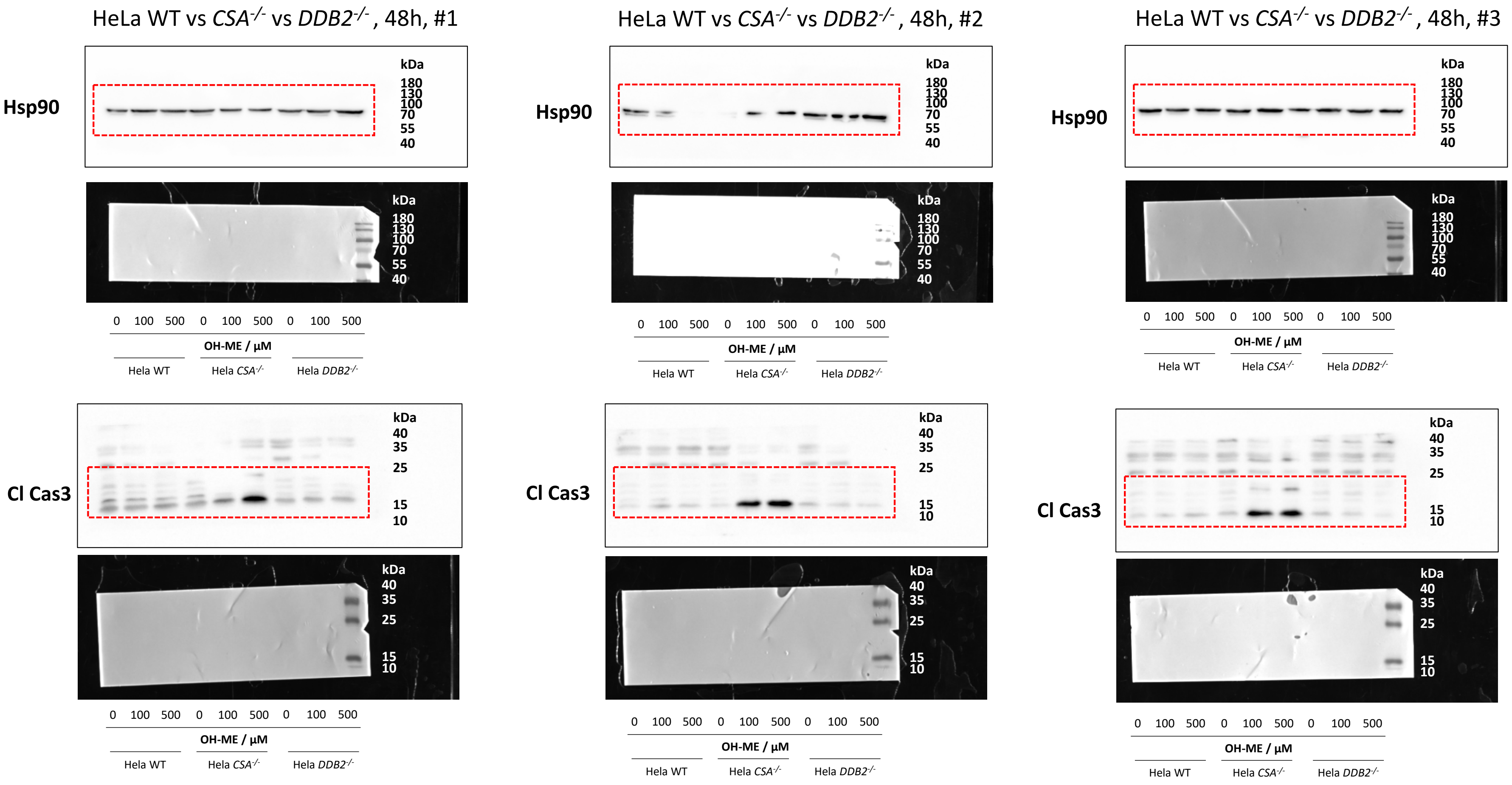

HeLa WT vs CSA-/- vs DDB2-/- , 48h, #1
HeLa WT vs CSA-/- vs DDB2-/- , 48h, #2
HeLa WT vs CSA-/- vs DDB2-/- , 48h, #3
Hsp90
Cl Cas3
kDa
180
130
100
70
55
40
35
25
15
10
0 100 500 0 100 500 0 100 500
OH-ME / µM
Hela WT
Hela CSA-/-
Hela DDB2-/-

Fig. S8A

HCT116

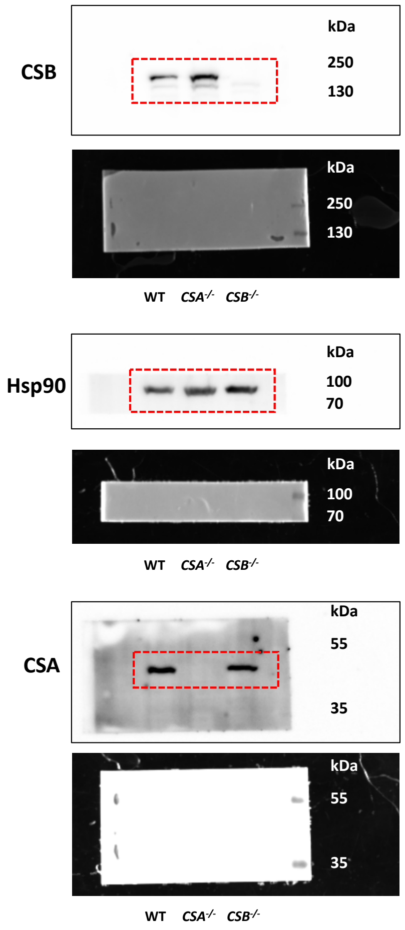

Fig. S12A and B and C

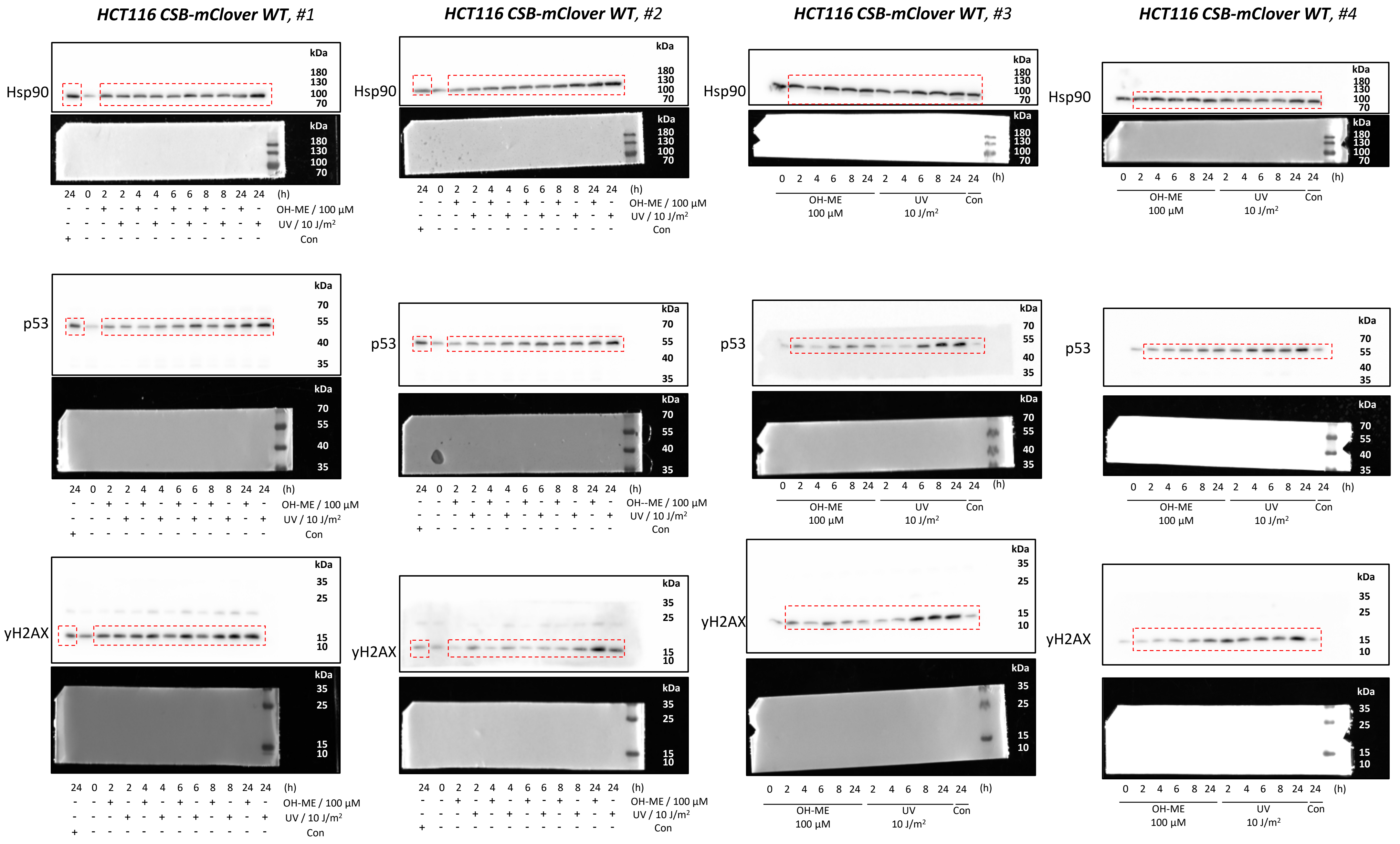

Fig. S12A and B and C

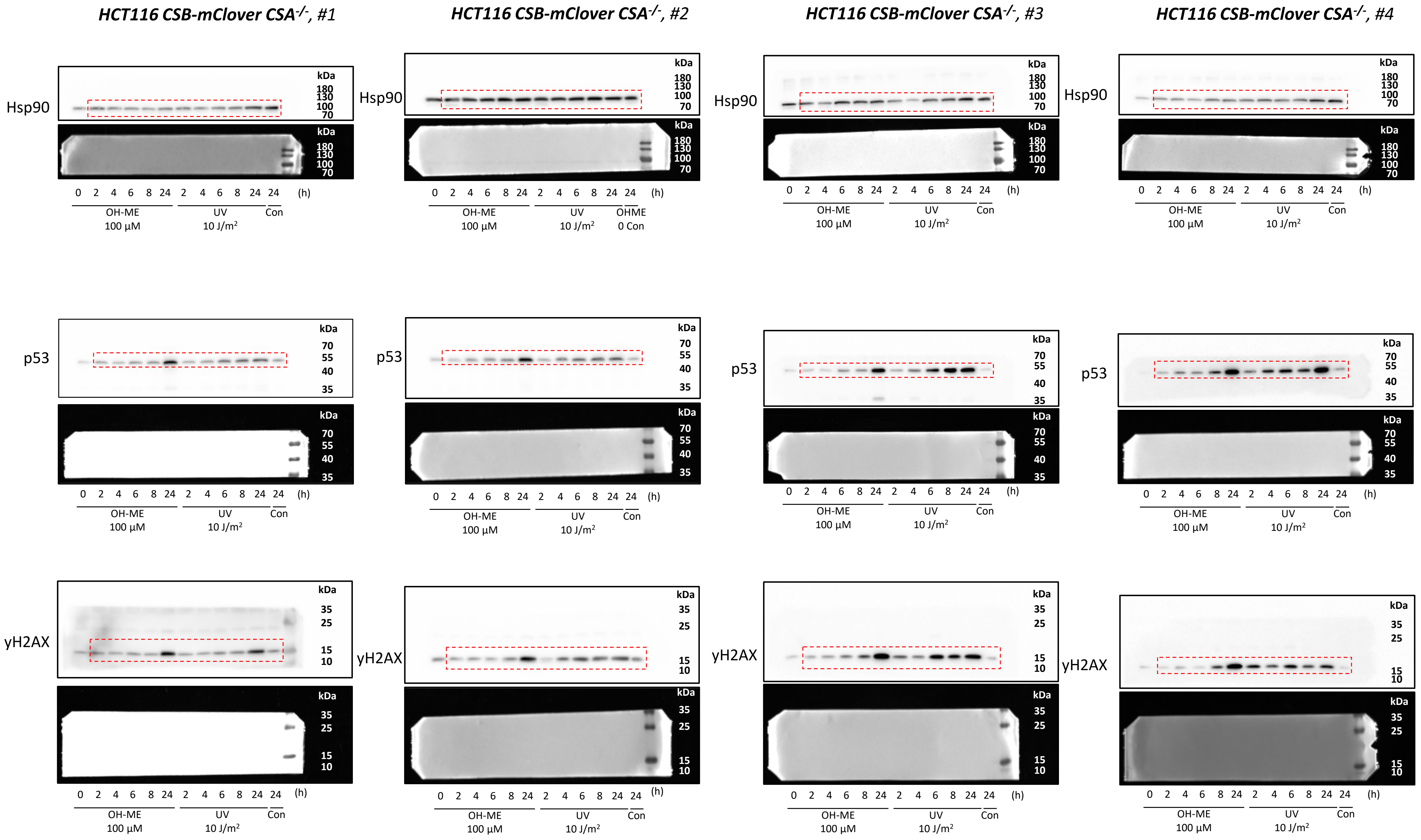

Fig. S12A and B and C

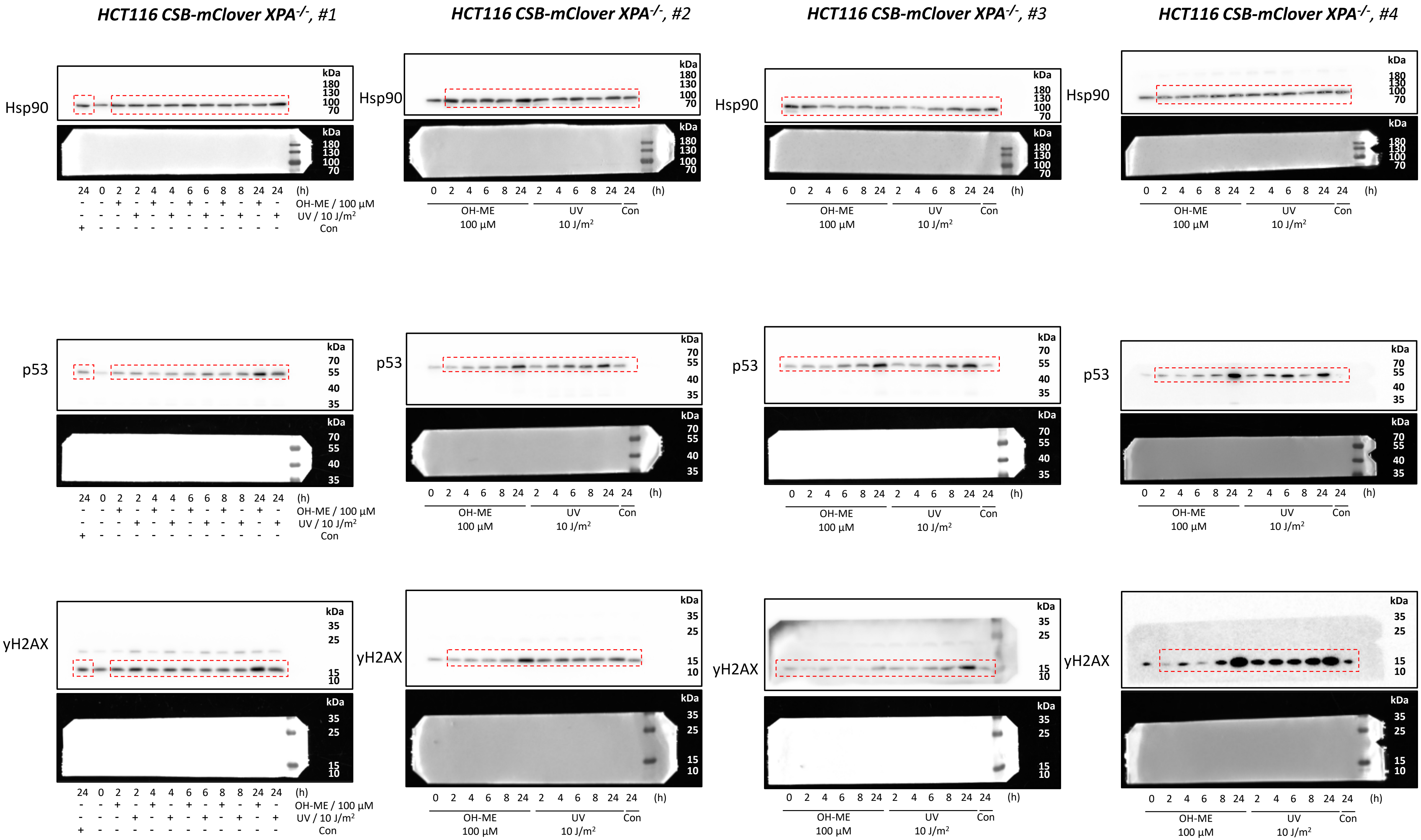

Supplement: Supplementary file 2 — Western Blot raw data [file 41419_2026_8853_MOESM2_ESM.pdf]
